# Large‐scale across species transcriptomic analysis identifies genetic selection signatures associated with longevity in mammals

**DOI:** 10.15252/embj.2022112740

**Published:** 2023-07-10

**Authors:** Weiqiang Liu, Pingfen Zhu, Meng Li, Zihao Li, Yang Yu, Gaoming Liu, Juan Du, Xiao Wang, Jing Yang, Ran Tian, Inge Seim, Alaattin Kaya, Mingzhou Li, Ming Li, Vadim N Gladyshev, Xuming Zhou

**Affiliations:** ^1^ Key Laboratory of Animal Ecology and Conservation Biology, Institute of Zoology Chinese Academy of Sciences Beijing China; ^2^ University of Chinese Academy of Sciences Beijing China; ^3^ School of Life Sciences University of Science and Technology of China Anhui China; ^4^ Integrative Biology Laboratory, College of Life Sciences Nanjing Normal University Nanjing China; ^5^ School of Biology and Environmental Science Queensland University of Technology Brisbane QLD Australia; ^6^ Department of Biology Virginia Commonwealth University Richmond VA USA; ^7^ Institute of Animal Genetics and Breeding, College of Animal Science and Technology, Sichuan Agricultural University Chengdu China; ^8^ Division of Genetics, Department of Medicine, Brigham and Women's Hospital Harvard Medical School Boston MA USA

**Keywords:** comparative transcriptomics, longevity, mammals, natural selection, Chromatin, Transcription & Genomics, Genetics, Gene Therapy & Genetic Disease, Methods & Resources

## Abstract

Lifespan varies significantly among mammals, with more than 100‐fold difference between the shortest and longest living species. This natural difference may uncover the evolutionary forces and molecular features that define longevity. To understand the relationship between gene expression variation and longevity, we conducted a comparative transcriptomics analysis of liver, kidney, and brain tissues of 103 mammalian species. We found that few genes exhibit common expression patterns with longevity in the three organs analyzed. However, pathways related to translation fidelity, such as nonsense‐mediated decay and eukaryotic translation elongation, correlated with longevity across mammals. Analyses of selection pressure found that selection intensity related to the direction of longevity‐correlated genes is inconsistent across organs. Furthermore, expression of methionine restriction‐related genes correlated with longevity and was under strong selection in long‐lived mammals, suggesting that a common strategy is utilized by natural selection and artificial intervention to control lifespan. Our results indicate that lifespan regulation via gene expression is driven through polygenic and indirect natural selection.

## Graphical abstract

Organ‐specific expression patterns of methionine restriction and translation fidelity genes correlate with longevity in 103 mammalian species.

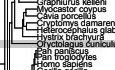

## Introduction

Over ~150 million years of evolution, mammals have diversified dramatically (over 100‐fold) in terms of longevity. This natural experiment has attracted much interest from biologists (Tacutu *et al*, 2013). Identifying lifespan‐related genetic variation, focusing primarily on exceptionally long‐lived species, has been a key approach to resolving this question. For example, a survey of the genome of the bowhead whale (the longest‐lived mammal known with a lifespan exceeding 200 years) revealed specific sequence changes in genes associated with DNA repair, cell cycle, and aging (Seim *et al*, 2014b; Keane *et al*, 2015a). Naked mole‐rats, which are the longest‐lived rodents (lifespan > 30 years), harbor unique variations in genes related to macromolecular degradation, mitochondrial function, and telomere maintenance, as well as tumor suppression (Kim *et al*, 2011a). Similarly, substitutions in genes related to the GH/IGF‐1 axis are found in the Brandt's bat, the longest‐lived flying mammal known (Seim *et al*, 2013a). Recent studies have also shown that elephants could be an attractive model organism for studying aging because they exhibit a long lifespan (> 50 years), a low cancer rate, and present an unexpected expansion of potentially functional TRP53 pseudogenes (Perez & Komiya, 2016; Sulak *et al*, 2016). These studies suggest a diversity in the genetic factors underlying mammalian longevity.

In addition to genetic variation, the lifespan of mammals is also likely to be modulated by the expression level of genes (Stern & Orgogozo, 2008). For example, *IGF1R* knockout leads to a 33 and 15.9% lifespan increase in female and male mice, respectively (Friedman & Johnson, 1988; Tatar *et al*, 2001; Holzenberger *et al*, 2003). Similarly, mTOR inhibition in mice increased the median lifespan of female and male mice by ~25% (Harrison *et al*, 2009; Kenyon, 2010; Miller *et al*, 2014). *SIRT6* overexpression increased the median lifespan of male mice by 14.5% (Kanfi *et al*, 2012). Comparing gene expression across species is challenging because variables such as developmental stages and environmental factors can mask or distort genuine expression differences. By assuming that gene expression is shaped primarily by selection, comparative transcriptomics studies have investigated gene expression patterns across species from an evolutionary perspective (Brawand *et al*, 2011b; Ma *et al*, 2015, 2016; Fushan *et al*, 2015a; Guschanski *et al*, 2017; Cardoso‐Moreira *et al*, 2019; Sarropoulos *et al*, 2019; Wang *et al*, 2020). For example, previous studies compared gene expression revealed many genes and pathways showing association with maximum longevity by comparing the liver, kidney, and brain tissues of 34 mammals (Fushan *et al*, 2015a) and cultured fibroblast cells of 16 mammals (13 rodents, two bats, and a shrew) (Ma *et al*, 2016). These studies found that the expression of genes related to central energy metabolism, DNA damage repair, sugar metabolism, and DNA repair was positively associated with longevity, whereas the expression of genes related to mitochondrial metabolism, transcriptional regulation, calcium‐mediated signaling pathways, protein ubiquitination, and protein localization was negatively associated (Fushan *et al*, 2015a; Ma *et al*, 2016). A comparison of age‐related transcriptomic changes in a long‐lived *Myotis* bat species, human, mouse, and wolf revealed that *Myotis* bats exhibit unique molecular mechanisms for lifespan extension in functions related to DNA repair, autophagy, immunity, and tumor suppression (Huang *et al*, 2019). These studies provide many insights into the relationship between gene expression variation and longevity traits across species. Nevertheless, the number of species analyzed in previous studies is relatively small and may not fully represent the diversity of gene expression by mammals.

To better characterize the expression profile of protein‐coding genes across the mammalian phylogeny, we generated transcriptome data from the brain, kidney, and liver tissues of 103 mammals, covering 16 orders and 45 families. First, gene expression in different organs and species‐specific expression patterns were assessed. Next, genes whose expression levels significantly correlated with longevity traits were identified within a phylogenetic framework. Pathways that showed gene expression signatures associated with longevity were identified using a modified summary approach. Finally, an integrated analysis of gene expression and selection pressure was carried out to measure selection intensity of the associated genes. The data and analyses presented in this study represent the most comprehensive cross‐species characterization of gene expression in mammalian organs to date and contribute to our understanding of how lifespan is regulated at the gene expression level.

## Results and Discussion

### Data generation and species‐specific gene expression

**Figure 1:**
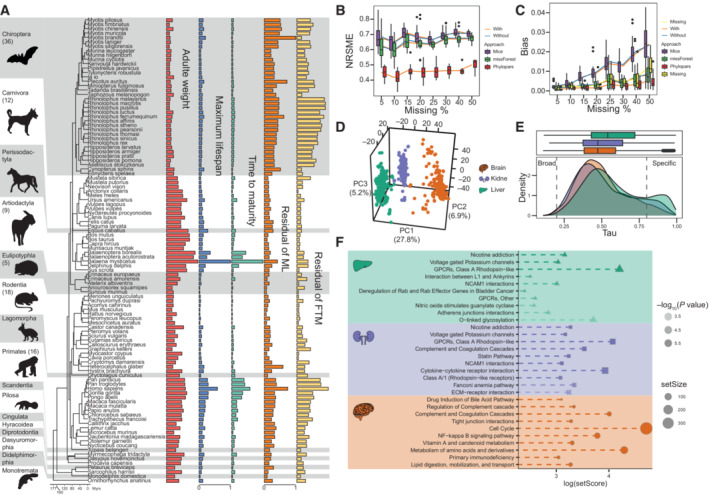
Life‐history traits and gene expression profile of mammals Mammalian phylogenetic tree with corresponding life‐history traits. From left to right: Adult weight (log_10_‐transform), maximum lifespan, female sexual maturity time, and residual of the maximum lifespan and female sexual maturity time relative to the adult weight. Each bar denotes a value of life‐history variable for a particular organism in standard scale. The animals image retrieved from PhyloPic (https://www.phylopic.org/).Estimation accuracy of missing values of life history traits: the *x*‐axis is the proportion of missing values, and the *y*‐axis is the standard root mean square error (NRSME).Estimation bias of missing values: the *x*‐axis is the proportion of missing values, and the *y*‐axis is the bias of biological significance.Principal component analysis of gene expression across tissues. The first three principal components (PCs) and their variance explanation percentages are shown. Each repetition is treated as a point.Distribution of species‐specific expression index (Tau) in the three tissues. Different organs are shown by different colors. The *x*‐axis is the species‐specific expression index; the *y*‐axis represents the frequency (below). The dotted line represents the threshold of the species‐specific expression index.Enrichment analysis of species‐specifically expressed genes for each tissue (Liver: green; Kidney: blue; Brain: orange). The *x*‐axis is the log‐transformed gene set score. The depth of the color represents the degree of significance of pathway enrichment, and dot size represents the size of the gene set. Data information: The boxes of the boxplot show the upper and lower quartile, the central band represents the median, and the whiskers represent the maximum and minimum values of the data. Outlier are represented by black points.

To capture the diversity of gene expression across mammals, we integrated transcriptomes from 103 species. We generated RNA‐seq data (~5.2 billion Illumina NovaSeq 6000 reads) from liver and kidney tissues of 56 species (Dataset EV1, Fig 1A, Appendix Figs S1 and S2, Materials and Methods). Brain data from the same 56 species were generated in a recent study (Data ref: Zhu *et al*, 2023a). Previously published RNA‐seq data from liver, kidney, and brain tissue of 47 additional species were also collected (Data ref: Brawand *et al*, 2011a; Data ref: Kim *et al,* 2011b; Data ref: Yan *et al*, 2011b; Data ref: NCBI Sequence Read Archive PRJNA163137, 2012; Data ref: Qiu *et al*, 2012; Data ref: Fan *et al*, 2013; Data ref: Seim *et al,* 2013b; Data ref: Seim *et al*, 2014a; Data ref: Fang *et al*, 2014; Data ref: Peng *et al*, 2015; Data ref: Fushan *et al*, 2015b; Data ref: NCBI Sequence Read Archive PRJNA323834, 2016; Data ref: NCBI Sequence Read Archive PRJNA349047, 2016; Data ref: NCBI Sequence Read Archive PRJEB13074, 2017; Data ref: Tang *et al*, 2017; Data ref: Carelli *et al*, 2018; Data ref: NCBI Sequence Read Archive PRJEB32966, 2019; Data ref: Chen *et al*, 2019; Data ref: Westbury *et al*, 2019; Data ref: Martínez‐Pacheco *et al*, 2020b) (Fig 1A, Appendix Figs S1A–C and S2A–C, Dataset EV1, Materials and Methods). After filtering and orthologs calling, a comprehensive expression dataset was obtained for 13,452 protein‐coding genes in three organs of 103 species (Materials and Methods). The dataset included the following orders: Artiodactyla (*n* = 9), Carnivora (*n* = 12), Chiroptera (*n* = 36), Cingulata (*n* = 1), Eulipotyphla (*n* = 5), Hyracoidea (*n* = 1), Lagomorpha (*n* = 1), Perissodactyla (*n* = 1), Pilosa (*n* = 1), Primates (*n* = 14), Rodentia (*n* = 17), and Scandentia (*n* = 1). In addition to 99 placental mammals, our dataset included the platypus (Monotremata), the Tasmanian devil (Dasyuromorphia), an opossum (Didelphimorphia), and the sugar glider (Diprotodontia) (Fig 1A, Dataset EV2). Information on adult weight (AW) and longevity‐related traits—including maximum lifespan (ML), female time to maturity (FTM), adult‐weight‐adjusted residuals (i.e., MLres and FTMres), and other life‐history traits (e.g., habitats and diet)—were also collected and analyzed (see Materials and Methods, Fig 1A, Dataset EV2). Among these traits, ML and FTM reflect changes in absolute longevity, and their residuals changes in relative longevity (Fig 1A). Three algorithms (i.e., mice, missForest, and Phylopars) were used to impute and estimate missing life‐history data for the species analyzed (Fig 1B and C, Appendix Fig S3A–H, Dataset EV2, Materials and Methods).

Principal component analysis was performed to assess gene expression patterns across species and tissues. Gene expression was tissue‐specific rather than lineage‐specific (Fig 1D), consistent with previous reports (Brawand *et al*, 2011b; Ma *et al*, 2015; Fushan *et al*, 2015a; Cardoso‐Moreira *et al*, 2019). The specificity index (Tau or τ) for gene expression was calculated to characterize genes with species‐specific expression patterns in a tissue (Dataset EV3, Materials and Methods). Tau ranges from 0 to 1 and indicates how broadly (< 0.2) or specific (> 0.8) a gene is expressed (Yanai *et al*, 2005). Compared to the liver and kidney, the brain presented the lowest number of genes showing species‐specific expression (792, 1,410, and 2,401 genes for the brain, kidney, and liver) (Fig 1E). In parallel, the brain had the highest number of broadly expressed genes (123, 85, and 37 genes for the brain, kidney, and liver) (Fig 1E). Broadly expressed genes likely reflect that they contribute to core biological processes of each organ. For example, amyloid precursor protein (*APP*) (τ = 0.11) was widely expressed in the brain. This gene participates in many important brain functions, including synaptogenesis, neuron cell proliferation, and differentiation (Czeczor & McGee, 2017). The most commonly expressed gene in the kidney was electron‐transferring flavoprotein α‐subunit (*ETFA*) (τ = 0.15). Inactivation of *ETFA* leads to multiple acyl‐CoA dehydrogenase deficiency, a serious mitochondrial disease (Kim *et al*, 2013). Complement protein 3 (*C3*) (τ = 0.11), a gene widely expressed in the liver, plays a central role in all three complement activation pathways (Reis *et al*, 2006).

In parallel, species‐specific genes mainly reflect the unique phenotypes or adaptations of particular groups. The gene with the highest species‐specific index in the brain was *CXCR3* (τ = 0.98). The primary role of this gene is to participate in inflammation as a chemokine receptor (Long & Jaiswal, 2000), and it may contribute to mouse cognition and behavior (Blank *et al*, 2016). The top species‐specific gene in the kidney was glutamate metabotropic receptor 1 (*GRM1*) (τ = 0.98), a gene that regulates cell proliferation (Martino *et al*, 2013). Deficiency of *GRM1* led to the inability to maintain the morphology and intracellular signal transduction in kidney podocytes (Puliti *et al*, 2011). *GRM1* was also highly expressed in long‐lived animals, such as bats, humans, and naked mole‐rats. This may be related to the supreme cell vitality of long‐lived animals (Link, 2019). In the liver, the most species‐specific genes, such as *SKOR1* (τ = 0.99), *NWD1* (τ = 0.99), and *KIAA1549L* (τ = 0.99), are associated with cancers (Correa *et al*, 2014; Heiss & Brenner, 2017; Sluimer *et al*, 2023) (Dataset EV3). SKI family transcriptional corepressor 1 (*SKOR1*) is also associated with type 2 diabetes. NK‐derived exocrine miR‐1249‐3p directly acts on *SKOR1* to regulate glucose homeostasis and remission of insulin resistance (Wang *et al*, 2021).

Pathway enrichment analysis was performed under a polygenic model to characterize pathways showing expression specificity across species in each organ (Daub *et al*, 2013, 2017) (Fig 1F, Dataset EV4, Materials and Methods). Pathways related to Alzheimer's disease, such as the NF‐κB signaling pathway (score = 43.80, *P* = 2.49 × 10^−6^), primary immunodeficiency (score = 20.94, *P* = 2.49 × 10^−6^), and tight junction interactions (score = 26.59, *P* = 2.49 × 10^−6^) were significantly enriched by genes showing expression specificity in the brain. In liver and kidney, genes with species‐specific expression enriched for cell growth and communication pathways such as nicotine addiction (liver: score = 66.84, *P* = 2.49 × 10^−6^; kidney: score = 26.30, *P* = 2.49 × 10^−6^), GPCRs class A Rhodopsin‐like (liver: score = 23.56, *P* = 2.49 × 10^−6^; kidney: score = 58.67, *P* = 2.49 × 10^−6^), NCAM1 interactions (liver: score = 20.34, *P* = 3.99 × 10^−5^; kidney: score = 22.30, *P* = 2.49 × 10^−6^), and voltage‐gated potassium channels (liver: score = 20.79, *P* = 2.49 × 10^−6^; kidney: score = 23.86, *P* = 2.49 × 10^−6^).

### Genes expression associated with maximum lifespan

Phylogenetic generalized least squares (PGLS) regression analysis was performed to identify genes showing a correlation between their expression and four longevity traits (ML, FTM, MLres, and FTMres) within a phylogenetic framework. Ordinary least squares (OLS), Brownian, and Ornstein–Uhlenbeck models were considered for each gene to determine the best correlation (Lavin *et al*, 2008; Ma *et al*, 2015, 2016). Based on resampling, the robustness of correlations was further evaluated through a two‐step verification process (see Materials and Methods for details) to minimize effects from outliers (*P*_*robust*_) or single species (*P*_*max*_) (Westfall & Young, 1993; Ma *et al*, 2015, 2016). Genes that met a *P*_*robust.adj*_ < 0.005 and *P*_*max.adj*_ < 0.05 threshold were considered significant (Table 1, Dataset EV5). Significant genes for more than two longevity‐related traits were defined as longevity‐correlated genes (*n* = 669, Table 1, Materials and Methods). Approximately 1.8% of the life‐history traits variation of the 103 mammals in our dataset could be explained by gene expression (Table 1). This is likely due to the higher number (*n* = 103) of species analyzed here, compared to a previous report that showed 11–18% of the inter‐species differences explained based on transcriptomes from 34 mammal species (Fushan *et al*, 2015a).

**Table 1:** Statistics on genes whose expression variation is correlated with life‐history variation.

Variable^a^	Liver (*n* = 13, 452)^b^		Kidney (*n* = 13, 136)		Brain (*n* = 13, 095)		
	No. of genes^c^	% from total	No. of genes	% from total	No. of genes	% from total	Combined^d^
Maximum lifespan	187 (73)	1.39 (0.54)	152 (80)	1.16 (0.61)	40 (29)	0.31 (0.22)	347 (26)
Female time to maturity	224 (114)	1.67 (0.85)	145 (77)	1.10 (0.59)	55 (41)	0.42 (0.31)	394 (30)
Maximum lifespan residual	303 (150)	2.25 (1.12)	295 (154)	2.25 (1.17)	336 (123)	2.57 (0.94)	860 (66)
Female time to maturity residual	325 (165)	2.42 (1.23)	285 (139)	2.17 (1.06)	505 (287)	3.86 (2.19)	1,022 (77)
Combined^e^	755 (268)	5.61 (1.99)	641 (213)	4.88 (1.62)	692 (230)	5.28 (1.77)	1,823 (669)^f^

^a^ The adjusted PGLS *P*‐value cutoff is *P*_*robust.adj*_ < 0.005 and *P*_*max.adj*_ < 0.05.

^b^ *n* denotes total number of orthologous groups assayed in the analysis.

^c^ Number of unique genes correlated with trait variation and number of genes specific for a trait (in brackets).

^d^ Number of unique genes identified in three organs for a specific trait and overlap in at least two organs (in brackets).

^e^ Number of unique genes identified in the organ and number of core genes in the organ (in brackets).

^f^ Number of unique genes identified in three organs for all traits and number of longevity‐correlated gene in all organs (in brackets).

**Figure 2:**
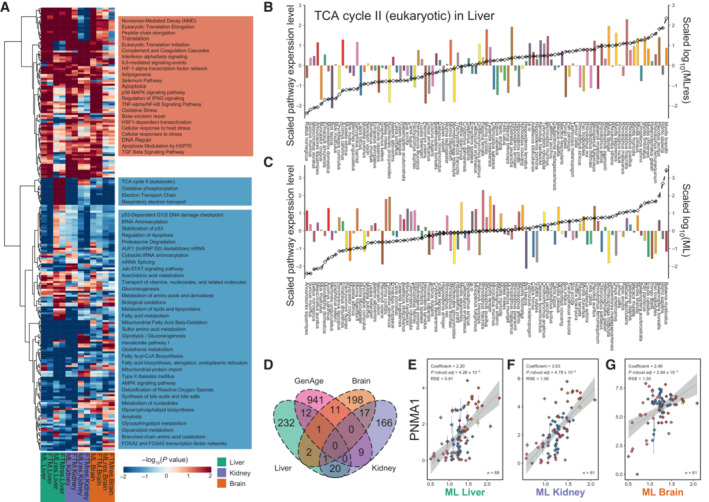
Relationship between gene expression variation and longevity AHeat map of the overlap pathways enriched by longevity‐related genes. The color intensity indicates the degree of significance, and the *P*‐value is transformed (−log_10_). Each row represents a different pathway, and each column represents traits related to longevity (marked at the bottom). Among them, red and blue colors show positive and negative correlations, respectively.B, CThe bar graph is the total expression of TCA cycle II (eukaryotic) longevity‐related genes in the liver of (B) MLres and (C) ML (*y*‐axis on the left). Black line is the relative value of life‐history variable (*y*‐axis on the right). Species are shown at the bottom. All values are in standard scale.DVenn diagrams of the three tissues for longevity‐correlated genes and aging genes of model organisms, obtained from the GenAge database.E–G
*PNMA1* is positively correlated with longevity traits in three tissues. In each figure, the *y*‐axis is the expression level and the *x*‐axis represents the longevity traits (ML: maximum lifespan; FTM: female time to maturity; MLres and FTMres: ML and FTM residuals adjusted for adult weight). Color point represents order. Error bars represents SE. Potential outliers have been removed. The coefficient of phylogenetic regression, *P.robust.adj* and residual standard error (RSE) is included in the figure.

We used the sum of the regression coefficient of each gene to identify pathways showing an enrichment with longevity (Daub *et al*, 2013, 2017) (Fig 2A, Appendix Fig S4, Datasets EV6 and EV7). Significantly enriched pathways related to the immune system and inflammation were detected in the liver (Appendix Fig S5). These included the complement cascades (ML: score = 13.66, *P* = 2.81 × 10^−4^; FTM: score = 10.81, *P* = 2.00 × 10^−5^), TNF‐α/NF‐κB signaling pathway (ML: score = 13.66, *P* = 2.75 × 10^−4^; FTM: score = 10.81, *P* = 2.00 × 10^−5^), regulation of IFN‐γ signaling (ML: score = 10.71, *P* = 3.34 × 10^−2^; FTM: score = 7.67, *P* = 1.36 × 10^−2^) and IL12‐mediated signaling events (ML: score = 19.25, *P* = 6.97 × 10^−4^; FTM: score = 11.58, *P* = 5.37 × 10^−4^). This enrichment is consistent with previous studies reporting that individuals with longer lifespans show an improved ability to resist inflammation (Youngman *et al*, 2011; Chen *et al*, 2014; Cheynel *et al*, 2017; Hilton *et al*, 2019). Interestingly, some well‐known aging processes were enriched by genes whose expression positively correlated with longevity. These included base excision repair (ML: score = 17.13, *P* = 8.00 × 10^−5^; FTM: score = 10.25, *P* = 5.90 × 10^−4^), TP53 network (ML: score = 10.32, *P* = 7.00 × 10^−5^; MLres: score = 10.81, *P* = 4.00 × 10^−5^; FTMres: score = 5.37, *P* = 1.26 × 10^−3^), and selenium pathway (FTM: score = 10.90, *P* = 9.71 × 10^−3^; MLres: score = 23.66, *P* = 1.37 × 10^−4^; FTMres: score = 18.24, *P* = 2.75 × 10^−5^). Pathways related to mitochondrial function (Appendix Fig S5), such as fatty acid metabolism (MLres: score = −28.70, *P* = 2.50 × 10^−6^; FTMres: score = −24.59, *P* = 2.50 × 10^−6^), the citric acid (TCA) cycle and respiratory electron transport (ML: score = −39.59, *P* = 2.50 × 10^−6^; FTM: score = −7.53, *P* = 3.55 × 10^−4^) (Fig 2B) and mitochondrial protein import (ML: score = −7.31, *P* = 1.77 × 10^−2^; FTM: score = −6.36, *P* = 2.85 × 10^−3^; FTMres: score = −12.42, *P* = 1.41 × 10^−3^), were enriched by genes that showed a negative correlation with longevity in the liver and also in kidney and brain. Previous study interrogated transcriptomes of 34 mammals and found that expressions of central energy metabolism genes negatively correlated with longevity traits (Fushan *et al*, 2015a). However, contrary to their results, the expression of TCA cycle II (MLres: score = 11.09, *P* = 5.00 × 10^−5^; FTMres: score = 8.10, *P* = 4.75 × 10^−5^) (Fig 2C), transport of glucose and other sugars, bile salts and organic acids, metal ions, and amine compounds (MLres: score = 33.01, *P* = 1.00 × 10^−5^; FTMres: score = 23.76, *P* = 2.25 × 10^−5^) and electron transport chain (MLres: score = 36.48, *P* = 2.11 × 10^−11^; FTMres: score = 28.63, *P* = 2.50 × 10^−6^) in liver increased significantly with MLres and FTMres gradients in our dataset (Fig 2A, Datasets EV6 and EV7). These genes showed the highest expression in bats, consistent with the considerable energy requirements of these flying mammals.

The top genes that with expressions positively correlated with longevity in the liver were *BCL7B*, *GATM*, *OSBPL9*, *PGP*, and *SLC9B2* (Appendix Fig S6). *BCL7B* inhibits carcinogenesis by regulating the Wnt signaling pathway (Uehara *et al*, 2015). *GATM* is associated with creatine synthesis (Humm *et al*, 1994), and knockout of *GATM* increases the production of ROS (Choi *et al*, 2021). *OSBPL9* overexpression in yeast inhibits the mTOR signal pathway and prolongs lifespan (Gebre *et al*, 2012). Among the top genes that with expressions negatively correlated with longevity in the liver (Appendix Fig S6), *ZNF710* (MLres: *P*_*robust.adj*_ = 4.37 × 10^−7^; FTMres: *P*_*robust.adj*_ = 1.07 × 10^−3^) ranked the first. While a role for this gene in aging and lifespan has yet to be established, a negative correlation between *ZNF710* expression and longevity was also reported in a study of 17 mammalian fibroblasts (Ma *et al*, 2016).

Pathways associated with cellular stress‐related functions showed enrichment for genes correlated with longevity in the kidney. These included HSF1‐dependent transactivation (FTM: score = 4.36, *P* = 1.11 × 10^−3^), HIF‐1α transcription factor network (FTM: score = 6.25, *P* = 2.68 × 10^−2^; MLres: score = 7.06, *P* = 3.53 × 10^−2^; FTMres: score = 13.26, *P* = 2.22 × 10^−4^), metabolism of xenobiotics by cytochrome P450 (ML: score = 12.07, *P* = 8.95 × 10^−4^; FTM: score = 7.00, *P* = 1.47 × 10^−3^; MLres: score = 10.85, *P* = 1.74 × 10^−3^), and regulation of IGF transport and uptake by IGFBPs (FTM: score = 4.26, *P* = 1.14 × 10^−3^). A role for *HIF‐1α*, the master regulator of hypoxia, is aging is well‐established (Yeo, 2019). *HSF1* (heat shock transcription factor 1) also plays a role in aging, for example, overexpression of *HSF1* prolongs the lifespan of *C. elegans* (Westerheide *et al*, 2009). Pathways, such as metabolism of lipids and lipoproteins (ML: score = −99.73, *P* = 2.50 × 10^−6^; FTM: score = −74.62, *P* = 2.50 × 10^−6^), metabolism of proteins (MLres: score = −82.40, *P* = 7.50 × 10^−6^; FTMres: score = −109.64, *P* = 7.50 × 10^−6^), metabolism of amino acids and derivatives (ML: score = −35.18, *P* = 2.50 × 10^−6^; FTM: score = −24.65, *P* = 2.50 × 10^−6^; MLres: score = −46.56, *P* = 2.50 × 10^−6^; FTMres: score = −35.81, *P* = 3.25 × 10^−5^) were enriched by genes whose expressions are negatively correlated with longevity (Appendix Fig S7).

The top genes whose expression positively correlated with longevity in the kidney were *PNMA1* (ML: *P*_*robust.adj*_ = 4.78 × 10^−8^; FTM: *P*_*robust.adj*_ = 3.21 × 10^−4^) and *OGDHL* (MLres: *P*_*robust.adj*_ = 1.75 × 10^−9^; FTMres: *P*_*robust.adj*_ = 1.43 × 10^−8^), both of which were also among top‐ranked genes in liver (Appendix Fig S8). *PNMA1* encodes a proapoptotic protein, and suppressing endogenous *PNMA1* expression decreases cell viability and promotes cell apoptosis (Chen & D'Mello, 2010; Jiang *et al*, 2014; Liu *et al,* 2018). Overexpression of *OGDHL* increases reactive oxygen species production and mediates *CASP3*, leading to cell apoptosis (Sen *et al*, 2012).

Pathways associated with oxidative damage repair showed enrichment for genes whose expression are positively correlated with longevity in the brain (Appendix Fig S9). These included oxidative stress (ML: score = 7.93, *P* = 6.67 × 10^−4^; FTMres: score = 5.77, *P* = 5.40 × 10^−3^), detoxification of reactive oxygen species (FTMres: score = 4.93, *P* = 6.90 × 10^−3^), base excision repair (ML: score = 13.74, *P* = 3.00 × 10^−5^; FTM: score = 5.89, *P* = 6.85 × 10^−3^), and FoxO signaling pathway (ML: score = 23.34, *P* = 2.04 × 10^−3^; FTM: score = 14.97, *P* = 2.45 × 10^−3^). Pathways related to central energy metabolism, the source of reactive oxygen species, were enriched by genes with expressions showing a negative correlation with longevity in the brain (Appendix Fig S9). They included mitochondrial protein import (FTM: score = −5.91, *P* = 1.42 × 10^−2^; MLres: score = −10.59, *P* = 1.51 × 10^−2^; FTMres: score = −10.04, *P* = 4.33 × 10^−3^), glycolysis and gluconeogenesis (FTMres: score = −11.04, *P* = 3.18 × 10^−3^), the citric acid (TCA) cycle and respiratory electric transport (FTM: score = −5.36, *P* = 1.73 × 10^−2^; MLres: score = −38.06, *P* = 2.50 × 10^−6^), and fatty acid biosynthesis, elongation, endoplasmic reticulum (MLres: score = −7.58, *P* = 4.00 × 10^−5^; FTMres: score = −4.89, *P* = 8.17 × 10^−4^). Other pathways enriched by negatively correlated genes are associated with Alzheimer's disease: amyloids (FTMres: score = −4.57, *P* = 1.45 × 10^−4^) and glycerophospholipid metabolism (ML: score = −14.60, *P* = 1.33 × 10^−3^; FTM: score = −12.55, *P* = 6.92 × 10^−4^). While the accumulation of amyloid protein lead to Alzheimer's disease (O'Brien & Wong, 2011), glycerol phospholipids can slow down the process of neurodegenerative diseases (Moré *et al*, 2014). Among the genes with expression positively correlated with longevity in the brain, many genes are associated with learning and memory (Appendix Fig S10). For example, *ADCY8* (MLres: *P*_*robust.adj*_ = 3.31× 10^−5^; FTMres: *P*_*robust.adj*_ = 2.01 × 10^−5^) and *KIF17* (MLres: *P*_*robust.adj*_ = 3.86 × 10^−6^; FTMres: *P*_*robust.adj*_ = 8.25 × 10^−4^) have roles in animal memory (de Quervain & Papassotiropoulos, 2006; Yin *et al*, 2011). In addition, expressions of genes associated with DNA repair and cancer suppression are positively associated with longevity. For example, *PMS2* (ML: *P*_*robust.adj*_ = 4.19 × 10^−3^; MLres: *P*_*robust.adj*_ = 3.86 × 10^−6^; FTMres: *P*_*robust.adj*_ = 4.14 × 10^−4^) is essential for DNA replication error repair (Shimodaira *et al*, 2003).

### Comparison of longevity‐correlated genes among tissues and gene sets

Unexpectedly, only one gene, *PNMA1* (PNMA family member 1), positively correlated with longevity in all three tissues examined (liver: ML‐*P*_*robust.adj*_ = 4.28 × 10^−6^, FTM‐*P*_*robust.adj*_ = 1.68 × 10^−3^; kidney: ML‐*P*_*robust.adj*_ = 4.78 × 10^−8^, FTM‐*P*_*robust.adj*_ = 3.21 × 10^−4^; brain: ML‐*P*_*robust.adj*_ = 2.84 × 10^−3^, MLres‐*P*_*robust.adj*_ = 1.63 × 10^−3^, FTMres‐*P*_*robust.adj*_ = 1.28 × 10^−4^) (Fig 2D–G). *PNMA1* (paraneoplastic Ma antigen‐like 1) encodes a protein that determines cell fate, with the outcome (e.g., apoptosis) possibly depending on cellular context or genetic background. Though previous studies have linked a causal relationship between cell division potential and maximum lifespan (The Hayflick limit) (Juckett, 1987), the role of *PNMA1* in lifespan control across species is worth exploring in future studies.

Next, we explored the intersection between longevity‐correlated genes and genes with known effects on aging in model organisms documented in GenAge (*n* = 974 genes) (Tacutu *et al*, 2013). Our longevity‐correlated genes showed enrichment for GenAge genes (*n* = 33; e.g., *CEBPB*, *MYC*, and *TERT*) (Fisher's exact test: *P* = 0.03, greater, odds ratio = 1.42). Nevertheless, most longevity‐correlated genes in our dataset are not known as aging‐related genes, indicating that most aging‐related genes do not serve as a basis for the evolution of longevity across species, although they have been shown to directly contribute to lifespan control in one or more model species.

**Figure 3:**
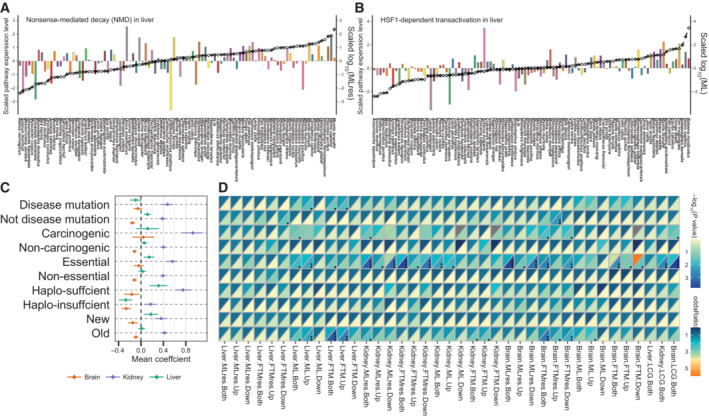
Longevity essential pathway and correlation among different gene categories and longevity‐related genes A, BThe bar graph is the total expression of (A) Nonsense‐Mediated Decay (NMD) and (B) HSF1‐dependent transactivation longevity‐related genes in the liver and brain, respectively (*y*‐axis on the left). Black line is the relative value of life‐history variable (*y*‐axis on the right). Species are shown at the bottom. All values are in standard scale.COn the *x*‐axis, the coefficient represents the rate of gene expression variation with life‐history gradient. Data are presented as means with error bars.DHeatmap of Fisher's exact test. The upper triangle heatmap represents the odds ratio and the lower the −log_10_(*P* value). The black point represents significance levels (*P* < 0.05: ·; *P* < 0.01: ··; *P* < 0.001: ···). LCG represents the longevity‐correlated gene.

Several common pathways are found to show correlation with longevity traits in the three tissues examined (Fig 2A, Dataset EV7). Positively correlated genes showed enrichment for transcription and translation fidelity pathways such as eukaryotic translation Initiation, eukaryotic translation elongation, nonsense‐mediated decay (NMD), peptide chain elongation and translation (Fig 3A and B), while the tRNA aminoacylation pathway was enriched for negatively correlated genes. The regulation of translation fidelity affects the lifespan of many organisms (von der Haar *et al*, 2017; Martinez‐Miguel *et al*, 2021). For example, the naked mole‐rat has higher translational fidelity than the mouse (Azpurua *et al*, 2013). Transfer RNA aminoacylation is inhibited in senescent cells to limit protein synthesis errors (Anisimova *et al*, 2020). In addition, seryl‐tRNA can directly bind to telomere repeat sequences, leading to telomere shortening and cell senescence (Li *et al*, 2019).

To understand the variation of longevity‐correlated genes derived from different genomic sources, we considered enrichment of longevity‐correlated genes within metrics of mutation tolerance (essential vs. non‐essential genes, disease‐harboring vs. non‐disease genes, and carcinogenic vs. non‐carcinogenic genes) and fitness (haplo‐sufficient vs. haplo‐insufficient genes, and new vs. old genes) (Materials and Methods). Compared with the liver and brain, the direction of longevity‐related genes in the kidney was mainly characterized by a positive correlation (i.e., between expression level and longevity), whereas the direction of correlation was more balanced in the liver and brain (Fig 3C). Longevity‐related genes enriched for essential genes (Liver‐ML‐Both *P* = 0.01, odds ratio = 1.58; Kidney‐FTMres‐Both *P* = 0.02 × 10^−2^, odds ratio = 1.80; Brain‐MLres‐Both *P* = 0.02 × 10^−2^, odds ratio = 1.72) and old genes (i.e., genes that originated before the emergence of mammals) (Liver‐FTM‐Both *P* = 0.03 × 10^−2^, odds ratio = 1.64; Kidney‐MLres‐Both *P* = 0.02, odds ratio = 1.31; Brain‐FTMres‐Both *P* = 0.01 × 10^−1^, odds ratio = 1.37) in all tissues (Fig 3D, Dataset EV8, Materials and Methods). Most essential genes were also old genes, indicating that expression changes of genes with essential functions in cells are important for lifespan control across species. In addition, we found that longevity‐related genes in the three tissues were enriched for carcinogenic genes (Liver‐ML‐Both *P* = 0.04, odds ratio = 2.69; Kidney‐MLres‐Both *P* = 0.03, odds ratio = 2.13; Brain‐FTMres‐Both *P* = 0.02, odds ratio = 1.83) and disease‐harboring genes were enriched only in the liver (*P* = 0.04, odds ratio = 1.36), hinting that carcinogenesis is a restrictive factor for organ aging and function.

### Relationship between selection pressure and gene expression

**Figure 4:**
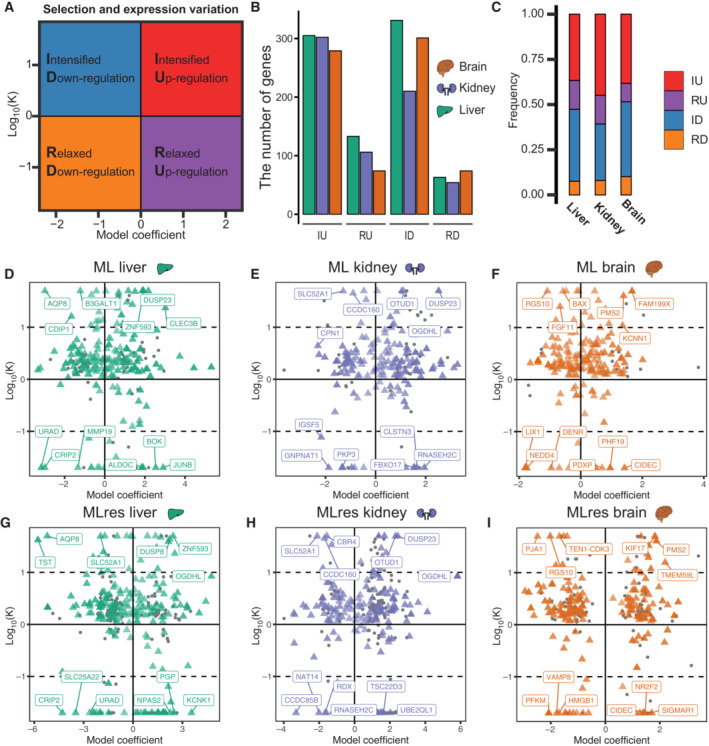
Pattern of association between selection index (*k*) and coefficients, and longevity AColored picture illustrating the annotation of gene classification.BNumber of genes in the three organs assigned to different gene classifications. IU stands for genes that are positively related to longevity and are under intensified selection; RU stands for genes that are positively related to longevity and are under relaxed choice; ID represents a gene that is negatively related to longevity and is under intensified selection; RD stands for genes that are negatively related to longevity and are under relaxed selection.CCumulative frequency histogram showing the distribution of gene types in different organs.D–IScatter plot showing the log_10_‐transformed relaxation parameter (*k*) on the *y*‐axis, and the variation rate of gene expression along the longevity trait gradient on the *x*‐axis. Dotted lines indicate quadrants with strongly intensified (*k* > 10) or relaxed (*k* < 10) selection pressure. The colored points represent longevity‐related genes (the color corresponds to the tissue type shown in panel B) and the gray points represent genes that are only significant related to this trait. The depth of the colored points represents −log_10_(*P*_*robust.adj*_).

We used RELAX to uncover the relationship between the intensity of selection and gene expression (Wertheim *et al*, 2015) (Dataset EV9). RELAX infers a selection intensity parameter, *k*, where *k* > 1 indicates intensified selection and *k* < 1 relaxed selection. Such genes can be under positive or purifying selection (estimated by ω). Long‐lived mammals were set as the foreground branch (ω_background branch_^*k*^ = ω_foreground branch_). Genes whose expression was correlated with longevity (i.e., longevity‐correlated genes) were divided into four categories (Fig 4A): positively correlated genes under intensified selection (IU), positively correlated genes under relaxed selection (RU), negatively correlated genes under intensified selection (ID), and negatively correlated genes under relaxed selection (RD).

Approximately 62% of genes were found to be under intensified selection in long‐lived mammals (defined as having an ML > 30 years) (Dataset EV9, Table EV1). Previous study has found genes tend to be under relaxed selection in shorter‐lived, annual killifish species (Cui *et al*, 2019, 2021). We also tested what type of selection was present at different maximum‐lifespan intervals (ML < 12; 12 ≤ ML ≤ 26; 26 < ML; 50 < ML) and found that species with longer lifespans tend to have more genes under intensified selection (Appendix Fig S11A–D, Datasets [Link], [Link], Table EV1). The pattern of selection intensity related to the direction of longevity‐correlated genes is different across organs (Fig 4B and C, Appendix Fig S12). A similar proportion of positively and negatively longevity‐correlated genes was under intensified selection in the liver and brain, however, a comparatively higher proportion of positively longevity correlated genes was under intensified selection in the kidney. It was also observed that several longevity‐correlated genes are under strongly intensified selection (*k* of 10 or above) in long‐lived mammals (Fig 4D–I, Appendix Fig S12). For example, *PMS2*, *OGDHL*, and *FAM199X* belong to the IU category, while *TST* belongs to the ID category. Moreover, the majority of longevity‐correlated genes are evolving under intensified or relaxed positive selection rather than purify selection (Table EV2).

We next performed pathway enrichment analysis using the relaxation parameter (*k*) of each gene in our dataset as a statistic to gauge the cumulative effect of relaxed and intensified selection on long‐lived mammals (Appendix Fig S13, Table EV3). Genes under intensified selection showed enrichment for the methionine salvage pathway (score = 8.41, *P* = 7.27 × 10^−4^) and glycerolipid metabolism (score = 21.31, *P* = 2.82 × 10^−3^). Methionine is one of the essential amino acids, and similarly to calorie restriction, methionine restriction has been reported to extend lifespan (Lam *et al*, 2017; Bárcena *et al*, 2018; Pradas *et al*, 2019), reverse inflammation, and reduce DNA damage (Sanchez‐Roman & Barja, 2013; Bárcena *et al*, 2018). Interestingly, our analysis revealed that several pathways correlated with accelerated aging are under relaxed selection in long‐lived mammals. These pathways included CD28 dependent PI3K/Akt signaling (score = −8.31, *P* = 8.69 × 10^−3^), WNT ligand biogenesis and trafficking (score = −10.42, *P* = 9.69 × 10^−3^), and the VEGF signaling pathway (score = −7.24, *P* = 4.43 × 10^−2^). Inhibition of these pathways extends lifespan (Boucher *et al*, 1998; Laurent *et al*, 2008; Rafalski & Brunet, 2011; Lezzerini & Budovskaya, 2014).

We further stratified our data set and considered two subgroups (i.e., Chiroptera and Rodentia). Pathways such as Wnt signaling, pluripotency, and HIV Infection were under relaxed selection in both subgroups (Table EV4). Specially, pathways related to transcription and translation fidelity and DNA replications were also significantly enriched by longevity‐correlated genes in both subgroups, as found in the analyses of the complete 103‐species dataset (Dataset EV13), further evidencing those pathways are important for lifespan evolution.

### Conclusions

This study generated a liver, brain, and kidney gene expression dataset of 103 species (56 sequenced for the first time by our laboratory) representative of diverse mammalian families. The dataset revealed species‐specific gene expression and genes showing expression correlation with longevity traits. While very few previously reported aging‐related genes correlated with longevity across the mammalian phylogeny, we identified many new candidate genes who may have roles in lifespan evolution (Appendix Figs S14A–I, S15 and S16). Of note, our results suggest that transcription and translation fidelity‐associated genes are essential to ensure mammal longevity. While other studies suggest no correlation between selection pressure and longevity (e.g., via gene expression) (Cui *et al*, 2019; Omotoso *et al*, 2021), our data show that selection shapes genes whose expression correlates with longevity traits as well as genes associated with longevity pathways (e.g., methionine restriction). Overall, the evolutionary correlation between gene expression and longevity is organ‐specific and characterized by polygenic selection.

A limitation of our study is the lack of samples from mature females. Therefore, sex differences in gene expression could not be accounted in our analysis (see Lopes‐Ramos *et al*, 2020). In addition, although we have attempted to control for batch effects (e.g., NCBI BioProject and sequencing platform), additional sources of unwanted variation exist (e.g., sample status, library preparation, genome quality, and ortholog calling). While it is difficult to estimate the actual age of wild samples accurately, these traits and factors should be explored in future studies. However, the data and results presented in this study are expected to aid future investigations. Especially, the identified longevity‐correlated genes could serve as candidate targets for further exploration of healthy aging.

## Materials and Methods

### Tissue collection

The 331 liver and kidney samples analyzed in this study were newly obtained from 56 species, while brain samples from 54 species retrieved from our previous studies (Zhu *et al*, 2023b) (Datasets EV1 and EV2). Moreover, the three tissues were processed at the same time, rather than separately processing the brain first. Liver, kidney and brain tissues were mostly sampled from adult and male individuals, if possible, and were freshly frozen in liquid N_2_ and stored at −80°C. To maximize sample compatibility, each major part of each organ was dissected and homogenized. To objectively detect the biological variation of gene expression, three biological replicates were obtained when possible. All the experimental protocols were approved by the Animal Care and Use Committee of the Institute of Zoology, Chinese Academy of Sciences (No. IOZ‐IACUC‐2021‐129).

### Transcriptome library preparation and sequencing

Total RNA was isolated from frozen tissue using TRIzol® Reagent (Invitrogen). To protect RNA as much as possible during homogenization, we first added 0.2 ml TRIzol® Reagent directly to a tube containing 100 mg of frozen tissue and homogenized using a motorized homogenizer. After homogenization, we added another 0.8 ml of TRIzol® to the tube. The resulting lysate was phase separated with 0.2 ml chloroform and total RNA precipitated with 0.5 ml isopropanol. The RNA was washed twice with 1 ml 75% ethanol and resuspended in DEPC treated ddH2O. The resuspended RNA was assessed for quality (260/280 nm absorbance ratio) and integrity (formaldehyde agarose gel electrophoresis). The sequencing libraries were prepared using the NEBNext Ultra RNA Library Prep Kit for Illumina (NEB, USA), and the transcriptome libraries were sequenced on an Illumina NovaSeq 6000 system (Novogene Co. Ltd) with paired‐end reads of 150 bp. NGS QC Toolkit v2.3.3 (Patel & Jain, 2012) was used to remove reads containing adapters and filter low‐quality reads (< Q20).

### Orthologous gene sets

Genome annotations (GTF) for 39 mammals with sequenced genomes were obtained from Ensembl, release 99. For the minke whale (*Balaenoptera acutorostrata*), Indian muntjac (*Muntiacus muntjac*), great roundleaf bat (*Hipposideros armiger*), Chinese rufous horseshoe bat (*Rhinolophus sinicus*), Brandt's bat (*Myotis brandtii*), François's leaf Monkey (*Trachypithecus francoisi*), and white‐footed mouse (*Peromyscus leucopus*) we used GTF annotations and genomes downloaded from NCBI database. For the bowhead whale (*Balaena mysticetus*) we used genomes downloaded from “The Bowhead Whale Genome Resource” (Keane *et al*, 2015b) (Dataset EV2). The GTF of bowhead whale was generated using augustus v 2.5.5 (Stanke *et al*, 2008). Draft transcriptome of 59 mammals were *de novo* assembled using trinity v2.11.0 (Grabherr *et al*, 2011). First, the RNA‐seq reads from same species and tissues were assembled together. Because the Trinity assembler filters low‐coverage *k*‐mers, we did not perform quality trim of the reads before assembly. Trinity was run on 150 bp paired‐end sequences with default parameters *k*‐mer size of 25 (fixed), minimum contig length of 200, maximum paired fragment length of 500, and adjusted butterfly maximum heap space setting to 30G. To remove redundancy, we then used cd‐hit (Li & Godzik, 2006; Fu *et al*, 2012) to process the assembled transcripts from different tissues of the same species, cluster the sequences with 90% similarity, and leave the longest transcript in each cluster. We used augustus to perform gene prediction on the de‐redundant transcripts and obtain GTF annotation files. We used gffread in the cufflink package v2.2.1 (Trapnell *et al*, 2010) to extract the CDS sequence, filtered out incomplete ORF transcripts and pseudogene transcripts, and extracted the longest transcript of each gene. Given the genome assembly for most of species are scarce or not well‐annotated, multi‐copy genes and transcripts were not considered in the analyses. To reduce the effects of paralogs on the ortholog identification, we constructed the human reference sequence using BLAST v2.9.0+ (Boratyn *et al*, 2013) to remove highly repetitive and highly similar genes, with *e*‐value < 10^−6^ and Identity > 90% as the filtering threshold. In the end, 18,553 unique protein coding genes were obtained as reference sequences. For other mammals, the longest transcript of each gene was extracted and reciprocal BLAST was performed with the protein sequences from human. The filtering threshold was 10^−6^ for *e*‐value and 30% for identity. Two genes that were best aligned with each other were defined as orthologous genes. When a gene exists in fewer species, it indicates that the gene is not highly conserved and cannot be representative of mammals. However, as the number of species increases, the number of orthologous genes that coexist in all species decreases (only 989 genes are present in all species). To balance the number of species and genes, we filtered out genes that exist in less than 68 species. The final dataset of orthologous gene accounted for 13,746 individual groups of sequences. In downstream analysis, each gene is analyzed individually, and only the species in which the gene is present were considered.

### RNA‐seq reads mapping and normalization

Because the complete genome and the *de novo* genome are quite different when compared, we used the CDS sequence of orthologous genes as the reference genome, and generate annotation files in GTF format for RNA‐seq data mapping. STAR v2.7.1a (Dobin *et al*, 2013) was used to construct an index. Because of the specificity of the orthologous genomes, the parameters “—genomeSAindexNbases” and “—genomeChrBinNbits” were calculated from the sequence size of the homologous gene set and the read length of different samples. And we used the default parameters to align the RNA‐seq data with the orthologous genomes. We used featureCounts v2.0.0 (Liao *et al*, 2014) to count reads, and eliminate multiple‐matched reads (Dataset EV14, Appendix Fig S1A–C). Generating gene expression profiles for all species based on pairwise orthologous relationships. Finally for 18,553 genes, abnormally low‐expressed genes that is, genes whose expression levels were less than 10 in four or more samples were filtered before normalization (2,564 genes were removed). And, abnormally high expression genes, that is, genes whose total expression of all samples accounted for 5% of the expression of the entire data set was also removed (1 gene). The function comBat_seq in the R package sva (Leek *et al*, 2012) was applied to correct for batch effect, including two factors that likely affect the data: data source (i.e., NCBI BioProject and sequencing batches of our data) and sequencing platform (Fukushima & Pollock, 2020). Tissue and species were set as fixed effects. We calculated the paired distance of samples (1‐Pearson's correlation coefficient) and excluded outlier samples and old/mature individuals (*n* = 24) (Dataset EV1, Appendix Fig S2A–C). To verify the effectiveness of batch correction, we performed PCA on these three datasets and conducted a Kruskal–Wallis rank sum test (K‐W test) on PC1 before and after batch correction (Table EV5). There was no significant difference between different BioProjects after correction (Appendix Supplementary Results). Moreover, the read type (short‐ vs. paired‐end reads) and sequencing platform had little effect on gene expression variation after batch correction (Appendix Supplementary Results). Genes with orthologous in more than 68 species were used for downstream analysis to reduce the false positive rate in the analysis (13,452 genes in total). We calculated the library size of each sample as a normalization factor. The R software package edgeR (Robinson *et al*, 2010) was used to normalize the library size and gene length (based on humans) by log_2_(TMM‐RPKM + 1). Log_2_‐transformation reduced the effect of higher expression value on the analysis and reduced the variation among replicates. For paired‐end data, featureCounts counts fragments, so calculating RPKM for paired‐end data is equivalent to FPKM.

### PCA analysis and species specificity of gene expression

We calculated the variance of each gene on the normalized expression matrix, and selected the top 5,000 genes with the largest variance to perform principal component analysis (PCA) using the R package “FactoMineR” (Lê *et al*, 2008). In order to define gene sets that are widely expressed by species and species‐specifically expressed genes. We calculated the mean value of log_2_(RPKM‐TMM + 1) in each organ for each species. We calculated the species‐specific expression index Tau, $\tau =\frac{\sum_{i=0}^{N}\left(1-x_{i}\right)}{N-1}$, which is used to quantify the tissue specificity of gene expression (Yanai *et al*, 2005). Among them, *N* is the number of species, $x_{i}$ is the expression level of the *i* ^th^ species. τ < 0.2 is defined as a broadly expressed gene. τ > 0.8 is defined as a species‐specific gene. For each ortholog, only species corresponding orthologous and expression values were considered in analyses.

### Life‐history data collection and imputation

To accurately estimate the species for which life history data were missing in this study. We collected data on highly correlated life‐history traits (AW: adult weight, ML: maximum lifespan, and FTM: female mature time) for a total of 1,250 species from the online databases AnAge (Tacutu *et al*, 2013), Animal Diversity Web (https://animaldiversity.org/) and PanTHERIA (Jones *et al*, 2009), and from the literature. And the phylogenetic tree was retrieved from TimeTree (http://www.timetree.org/) (Kumar *et al*, 2017). Three life‐history traits from 816 species were complete and used as a training set and, we employed three imputation methods to estimate the missing data: (i) Based on the Markov chain Monte Carlo method, *mice* introduces the random process into the interpolation process, uses other variables as predictors, and specifies a conditional model for each variable (van Buuren & Groothuis‐Oudshoorn, 2011). We used the predictive mean matching (pmm) as the conditional model in the multiple regression model, or used mean matching (mean) instead if the first run did not converge. We selected the case where the predicted regression score was closest to the missing value. (ii) missForest first uses the mean to interpolate a column of data (Stekhoven & Bühlmann, 2012) and then uses the remaining variables of the data set to fit a random forest model to estimate missing values by applying trained random forest predictions. This process was looped for all variables that need to be interpolated, and the whole process was repeated until the stop criterion was reached. (iii) Phylopars estimates missing values based on restricted maximum likelihood (Bruggeman *et al*, 2009). This method calculates the covariance matrix based on phylogenetic and phenotypic components (when multiple trait measures are given). It builds a multivariate normal model that combines the best phylogenetic and phenotypic covariance with the tree to calculate the covariance between the observed and missing values. Therefore, the estimated value was determined by phylogenetic distance (correlation between species) and ectopic relationship (correlation between features).

The percentages of missing values in the missing set were 6% (ML) and 30% (FTM) (Appendix Fig S3A). We tested three types of missing data: (i) Completely missing at random (MCAR); (ii) Missing at random based on weight (MAR.AW), and it was divided into two types of species according to the median weight. Because low‐weight species may have more missing values; (iii) Missing at random based on the genetic distance between human (MAR.HD), and it was divided into two types of species according to the half of the farthest genetic distance (Appendix Fig S3B). Because species with a greater genetic distance from humans may receive less attention from scientists, the life‐history is also opted to be missed.

We performed chi‐square tests on the two types of MARs in the missing set (Appendix Fig S3C–F). In addition, missing values in large‐weight species accounted for 17.57% of the total missing values in maximum lifespan (ML), and 82.43% in small‐weight species. In FTM, the missing values of the large‐weight species accounted for 37.66% of the total missing values, and the small‐weight species 62.34%. We introduced multiple missing value ratios (5, 10, 15, 20, 25, 30, 40 and 50%) to the training set to simulate the distribution and pattern of missing values. We used the above three methods for 10 interpolations and imputation. In order to account phylogenetic relationships in the imputation process (Phylopars are only applicable to imputations that include phylogeny), R package PVR was used (Santos, 2018) to perform principal coordinate analysis (PCoA) on the genetic distance matrix of 816 species to obtain phylogenetic feature vectors. The phylogenetic relationship after dimensionality reduction is used as other predictor variables in the imputation process. At the same time, we obtained the optimal number for interpolation by adding phylogenetic vectors in the interpolation process incrementally.

We evaluated the accuracy of the interpolation based on the normalized root mean square error (NMRSE) as $\text{NRMSE}=\frac{\sqrt{\text{mean}\left({\left(\text{Ximp}-\text{Xtrue}\right)}^{2}\right)}}{\max\left(\text{Ytrue}\right)-\min\left(\text{Ytrue}\right)}$.

And, in order to ensure that the estimated value retains biological significance. We also calculated the bias of the slope $\left(\text{Bias}=|{\text{Slope}}^{\text{original}}-{\text{Slope}}^{\text{imputed}\;\left(\text{or missing}\right)}|\right)$ between adult weight and maximum lifespan in the data set after interpolation (Fig 1B and C).

Finally, we selected the best Phylopars based on the evaluation results for the imputation of the complete life history data set (Dataset EV2). To identify confounding factors of maximum lifespan, we collected multiple complex effects such as society, diet, habitat, activity, body mass, basal metabolism rate, and offspring per year. We have used MCMCglmm to examine the correlation between longevity and other factors. The result showed that body mass and offspring per year were significantly associated with maximum lifespan, and a weak association between diet and maximum lifespan (Table EV6). And we also detected a strong correlation between body mass and offspring per year. Previous studies also have shown that the longevity (or female time to maturity) was mainly correlated with body mass (Fushan *et al*, 2015a). Nevertheless, many species show with small weight and long maximum lifespan (or female time to maturity). Therefore, we calculated the residuals to correct the confounding effects caused by weight (i.e., MLres and FTMres). Both residual equations are obtained based on linear regression model using the data from the AnAge database (Tacutu *et al*, 2013).

### Phylogenetic regression analysis

To identify genes with the expression related to longevity, three evolution models were tested for gene expression in each tissue (mean value of log_2_‐scaled TMM‐RPKM) and each log_2_‐scaled longevity‐related trait (ML, FTM, MLres, FTMres), including regression models that do not consider phylogenetic relationships (OLS), and regression models that consider phylogenetic relationships (BM and OU). And the optimal model was selected according to the maximum likelihood methodology. The phylogenetic tree was retrieved from TimeTree (Kumar *et al*, 2017). The unit of branches length of the phylogenetic tree is million years. To avoid randomness, we took a resampling approach (Westfall & Young, 1993) instead of using conventional *P*‐value corrections (e.g., BH). A two‐step method is used to correct the *P*‐value (Ma *et al*, 2015, 2016). In the first step, the species that has the greatest impact on the slope (i.e., potential outliers) is removed by the residuals (the largest absolute value of the residual is removed), and then the regression is performed again. At this time, the *P*‐value obtained is defined as *P*_*robust*_ to remove the influence of the outliers on the regression. The second step is to repeat the regression process for the remaining species and remove one of remaining species each time until all remaining species are removed once, and take the largest (least significant) *P*‐value in the process as *P*_*max*_ to remove the impact of species on regression. The cutoff for identifying longevity‐related genes was *P*_*max.adj*_ < 0.05, *P*_*robust.adj*_ < 0.005. To reduce the noise caused by missing data, for each gene, we only consider the species in which the gene exists, and did not add all species to the model, that is, set the expression value of a gene that does not exist in a species to the missing value instead 0. We denote genes associated with two or more longevity traits as longevity‐correlated genes.

### Multiple sequence alignment and selection pressure analysis

For each group of orthologous genes, the Perl script “translatorX.pl” (Abascal *et al*, 2010) is used for multiple sequence processing and comparison. This pipeline selects the default parameters of the “MAFFT” (Katoh *et al*, 2002) to first translate the nucleic acid sequence into a protein sequence for multiple sequence alignment and then translate it back into a nucleic acid sequence. We then used “GBlock” (Castresana, 2000; Talavera & Castresana, 2007) to select the conservative blocks, with the number of conservative sites in the gene sequence after alignment is at least 75% of the total length of the gene, and the shortest flanking sequence is greater than 85% of the length of the gene after the alignment.

In order to test whether genes are under relaxed selection, we used the minimal model of RELAX (Wertheim *et al*, 2015) in “Hyphy,” with long‐lived animals (ML > 30 years) were set as foreground branches. To define a cutoff for long‐lived species, we used the Partitioning Around Medoids (PAM) algorithm to cluster longevity data of 974 species (Zhu *et al*, 2023b) into two groups (long‐lived group and non‐long‐lived group) by setting the parameter *k* (the number of clusters) as two (Kaufman & Rousseeuw, 1990). The range of maximum lifespan was 1.00–17.30 years (428 species, mean = 8.88 years) for the non‐long‐lived group and 17.40–211 years (536 species, mean = 30.49 years) for the long‐lived group. Therefore, we set the ML cut‐off of long‐lived mammals at 30 years, which is also close to the 26 years cut‐off suggested by Jobson *et al* (2010). Moreover, none of the estimated maximum lifespans are larger than 30 years (among the 103 species in this study), which could alleviate the potential errors resulting from imputation. Because the number of non‐long‐lived mammals (*n* = 82) is far more than that of long‐lived mammals (*n* = 24). We selected 24 representative species with good genome quality from non‐long‐lived mammals as background branches to eliminate the noise caused by the excessive number of background branch species (Table EV1). This model uses the likelihood ratio test to compare the two models with the same evolution rate (*k* = 1) and different rates (*k* ≠ 1) between the foreground branch and the background branch. The parameters are set to estimate three types of ω (ω1: purification selection; ω2: neutral selection; ω3: positive selection). The relaxation parameter *k* is an index of the selection strength (ω_background branch_^*k*^ = ω_foreground branch_), with *k* > 1 indicates that the genes in the foreground branch are under intensified selection and *k* < 1 indicates a relaxed selection. And we also test relaxed selection at different interval (ML < 12; 12 ≤ ML ≤ 26; 26 < ML; 50 < ML) of ML by use 57 mammals which have real life‐history traits data (Datasets [Link], [Link], Table EV1). For Chiroptera and Rodents, the cut‐off for longevity of were set at 23 and 14 years old (base on upper quartile), respectively.

### Gene set enrichment analysis

We use “Polysel” (Daub *et al*, 2013, 2017) for gene set enrichment analysis which is possible to detect pathways containing pleiotropic signals. In addition, other variables (such as gene length, number of species, and genetic distance) can also be used to adjust statistical variables. For the species‐specific expression, we used the species‐specific expression index (Tau) as the gene score (SUMSTAT) to detect species‐specific expression pathways and ubiquitous expression (1 − τ) pathways. For gene expression variation, we used the coefficients in the PGLS regression as SUMSTAT to enrich genes that are positively related to longevity (SUMSTAT of negatively related genes is set to 0) and negatively related genes (SUMSTAT of positively related genes is set to 0 and converted to Absolute value) to detect longevity‐related pathways with genetic minor effects. Since gene expression is mostly related to gene length or species number, we used the function “RescaleBins” to adjust SUMSTAT. We used “ks.test” in R to check whether the gene score (SUMSTAT) is normally distributed or not. If not, 400,000 random data set was generated to construct an empirical distribution.

### Gene category collection

We collected different types of gene sets from various sources for comparison. The essential genes were constructed based on the probability of intolerance to loss of function, which is the pLI score (Lek *et al*, 2016). The score data comes from ExAC version 0.3.1 (https://gnomad.broadinstitute.org/). Genes with pLI > 0.9 are defined as essential genes. The list of genes associated with human inherited disease was obtained from the manually curated HGMD (PRO 17.1) (Stenson *et al*, 2017). Cancer‐related genes were obtained from The Cancer Gene Census (CGC) (https://cancer.sanger.ac.uk/census) (Tate *et al*, 2018). Aging genes were obtained from the GenAge database (Tacutu *et al*, 2013) and determined based on experimental evidence from humans and model organisms. They included genes related to the basic human aging process as well as genes related to lifespan. According to the homology relationship, the respective gene ID numbers were converted into human gene ID numbers. The Haploid Insufficiency (HI) score from previous studies (Shihab *et al*, 2017) was used to quantify the degree of haploid deficiency in human genes. After sorting in descending order, we defined genes greater than the first quartile as haplo‐insufficient genes, and genes less than the fourth quartile as haplo‐sufficient genes. Finally, the phylogenetic age of mammalian genes was retrieved from the GenTree database (http://gentree.ioz.ac.cn/) (Shao *et al*, 2019). We divided genes into two groups based on genetic age: (i) those genes that appear after therian, mammalian, vertebrate, or quadrupedal ancestors (genes are defined as relatively young) and (ii) those that appear earlier than bone vertebrates Genes (defined as relatively old genes). We conducted gene enrichment analysis of positively correlated genes (Up), negatively correlated genes (Down) and all genes (Both) using Fisher's exact test.

## Author contributions

**Weiqiang Liu:** Data curation; formal analysis; validation; visualization; methodology; writing – original draft. **Pingfen Zhu:** Data curation; writing – original draft. **Meng Li:** Validation; investigation. **Zihao Li:** Resources; validation. **Yang Yu:** Resources; visualization. **Gaoming Liu:** Resources; data curation; formal analysis. **Juan Du:** Resources. **Xiao Wang:** Resources; project administration. **Jing Yang:** Resources; data curation. **Ran Tian:** Writing – original draft. **Inge Seim:** Visualization; writing – original draft; writing – review and editing. **Alaattin Kaya:** Writing – original draft; writing – review and editing. **Mingzhou Li:** Resources; writing – original draft; writing – review and editing. **Ming Li:** Resources; data curation; supervision. **Vadim N Gladyshev:** Conceptualization; writing – review and editing. **Xuming Zhou:** Conceptualization; resources; data curation; supervision; funding acquisition; writing – original draft; project administration; writing – review and editing.

## Disclosure and competing interests statement

The authors declare that they have no conflict of interest.

## Supporting information



Appendix

Table EV1

Table EV2

Table EV3

Table EV4

Table EV5

Table EV6

Dataset EV1

Dataset EV2

Dataset EV3

Dataset EV4

Dataset EV5

Dataset EV6

Dataset EV7

Dataset EV8

Dataset EV9

Dataset EV10

Dataset EV11

Dataset EV12

Dataset EV13

Dataset EV14

## Data Availability

The RNA sequencing data generated in this study have been deposited in the Genome Sequence Archive (Chen *et al*, 2021) in National Genomics Data Center (CNCB‐NGDC Members and Partners, 2022), China National Center for Bioinformation/Beijing Institute of Genomics, Chinese Academy of Sciences under accession code GSA: CRA009075 (https://ngdc.cncb.ac.cn/gsa/browse/CRA009075). The gene expression matrix and corresponding meta information are available at GitHub (https://github.com/liu‐wq/expressionML.git).
