# Supplementary material for: Large‐scale across species transcriptomic analysis identifies genetic selection signatures associated with longevity in mammals
Source: EMBO J. 2023 Jul 10;42(17):e112740. doi: 10.15252/embj.2022112740 (PMC10476176; doi:10.15252/embj.2022112740)
Supplement: Supplementary file 1 — Appendix [file EMBJ-42-e112740-s010.pdf]

Appendix Materials for

Transcriptomic and selection signatures of longevity in mammals

Weiqiang Liu, Pingfen Zhu, Meng Li, Zihao Li, Yang Yu, Gaoming Liu, Juan Du, Xiao Wang, Jing Yang, Ran Tian, Inge Seim, Alaattin Kaya, Mingzhou Li, Ming Li, Vadim N. Gladyshev, Xuming Zhou

Corresponding author: Xuming Zhou, zhouxuming@ioz.ac.cn

The PDF file includes:

Appendix results -------- Page 2- Page 3

Appendix Fig S1 to S19-------- Page 4- Page 36

Appendix Results

Batch effect assessment

To verify the effectiveness of batch correction, we selected several subsets to compare the batch correction of BioProject, reads type (short- vs. paired-end reads), and sequencing platform. The data were mainly from three sources: new sequencing data generated in this study and data generated by Fushan *et al.* (2015) and Brawand *et al.* (2011). We performed PCA and conducted a Kruskal-Wallis rank sum (K-W) test on PC1 before and after batch correction. The results showed that before correction PC1 separates the samples by tissues but also by BioProject (Kruskal-Wallis chi-squared = 30.19, P = 0.02). After batch correction, PC1 separated the samples by tissue but not by BioProject (Kruskal-Wallis chi-squared = 15.30, P = 0.50). In addition, we examined each tissue separately and consistently found that the main components did not distinguish the samples by BioProject after removing batch effect (Liver: P = 0.71; Kidney: P = 0.04; Brain: P = 0.05) (**Appendix Fig S17** and **Table EV5**).

We then tested the influence of the read type using the data set generated by Brawand *et al*. (2011) (NCBI BioProject: PRJNA143627). This data set includes brain RNA-seq reads of different types (76bp single-end and 100bp paired-end) generated on the Illumina Genome Analyzer IIx platform. PC1 and PC2 separated samples by species but not read type before and after batch removal (**Appendix Fig S18**), indicating the read type has a limited influence on gene expression variation.

Finally, we used the data from the ferret (*Mustela putorius*) and sugar glider (*Petaurus breviceps*) to examine the batch effect of sequencing platforms. The ferret data was generated on the Illumina HiSeq 2000 platform, and the sugar glider data using the Illumina Genome Analyzer IIx. Our data from the same species were generated on the Illumina Novaseq 6000 instrument. PC1 and PC2 separated samples by species but not by

sequencing platforms (**Appendix Fig S19**), indicating that the sequencing platform does not significantly affect gene expression.

Appendix Figure

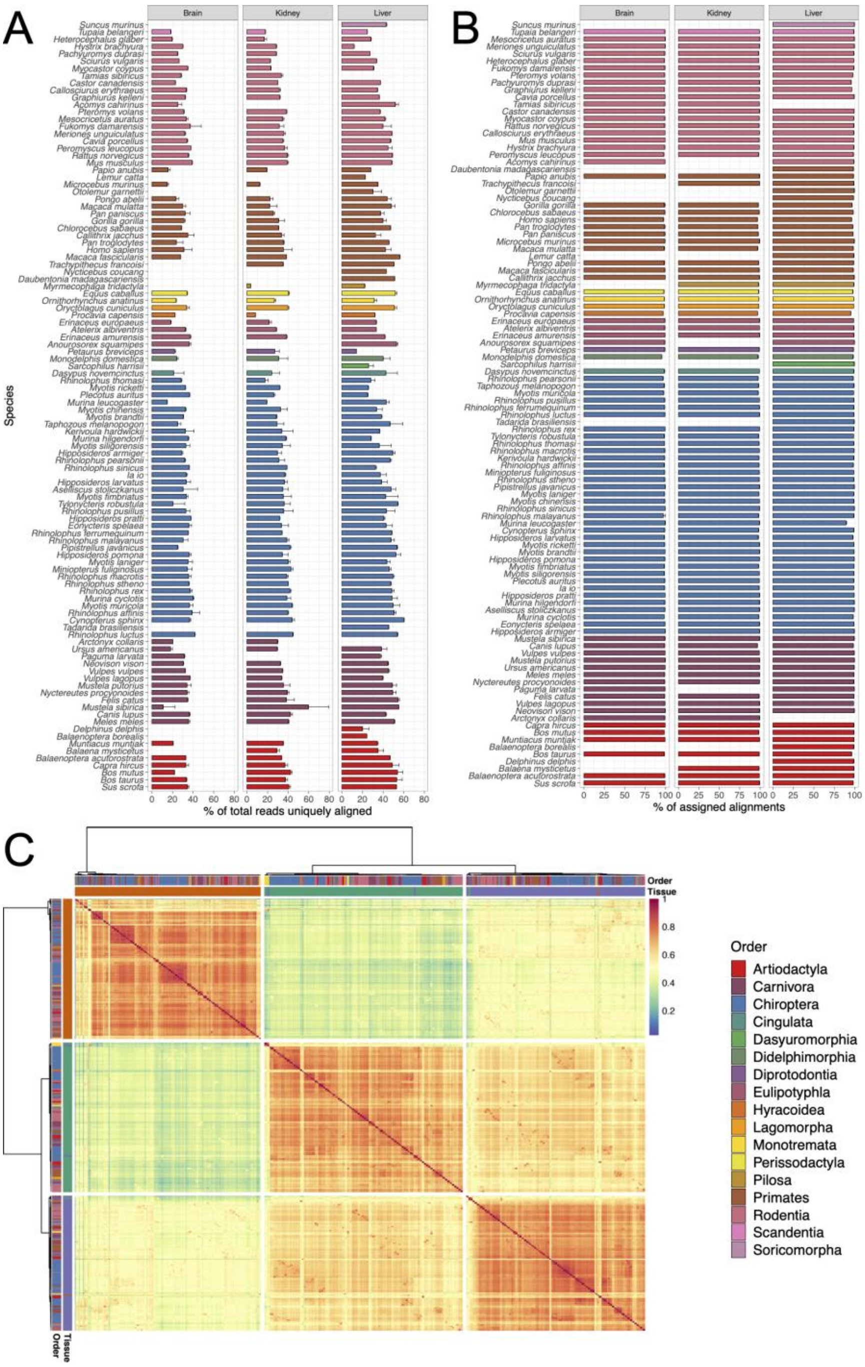

Appendix Fig S1 Sample alignment statistics and inter-sample correlation. (*A*) RNA-seq unique mapping rates in each tissue. (*B*) The ratio of reads assigned to genes in each tissue. (*C*) Heatmap of correlation coefficient between samples. All group colors represent different order.

A

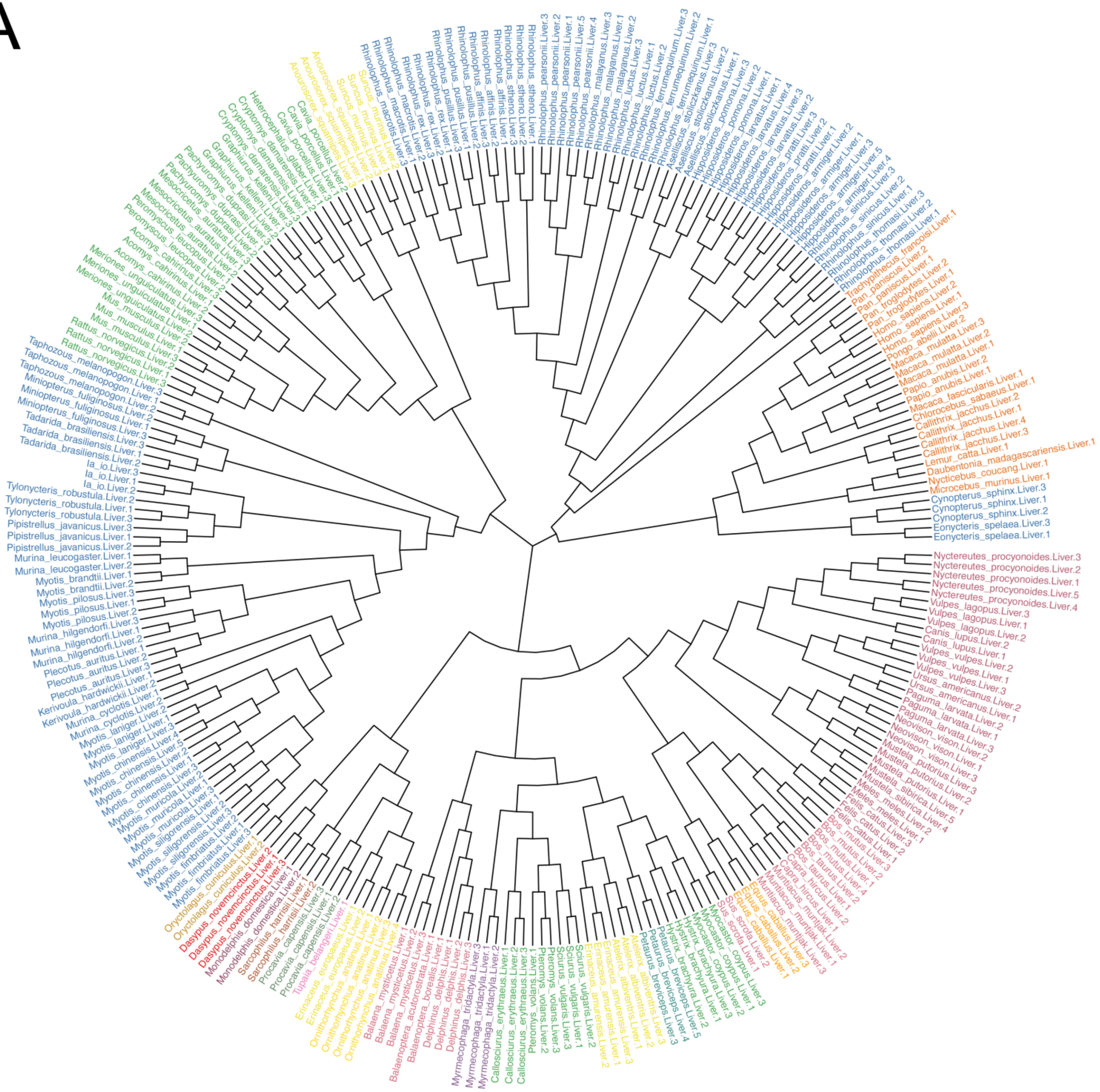

B

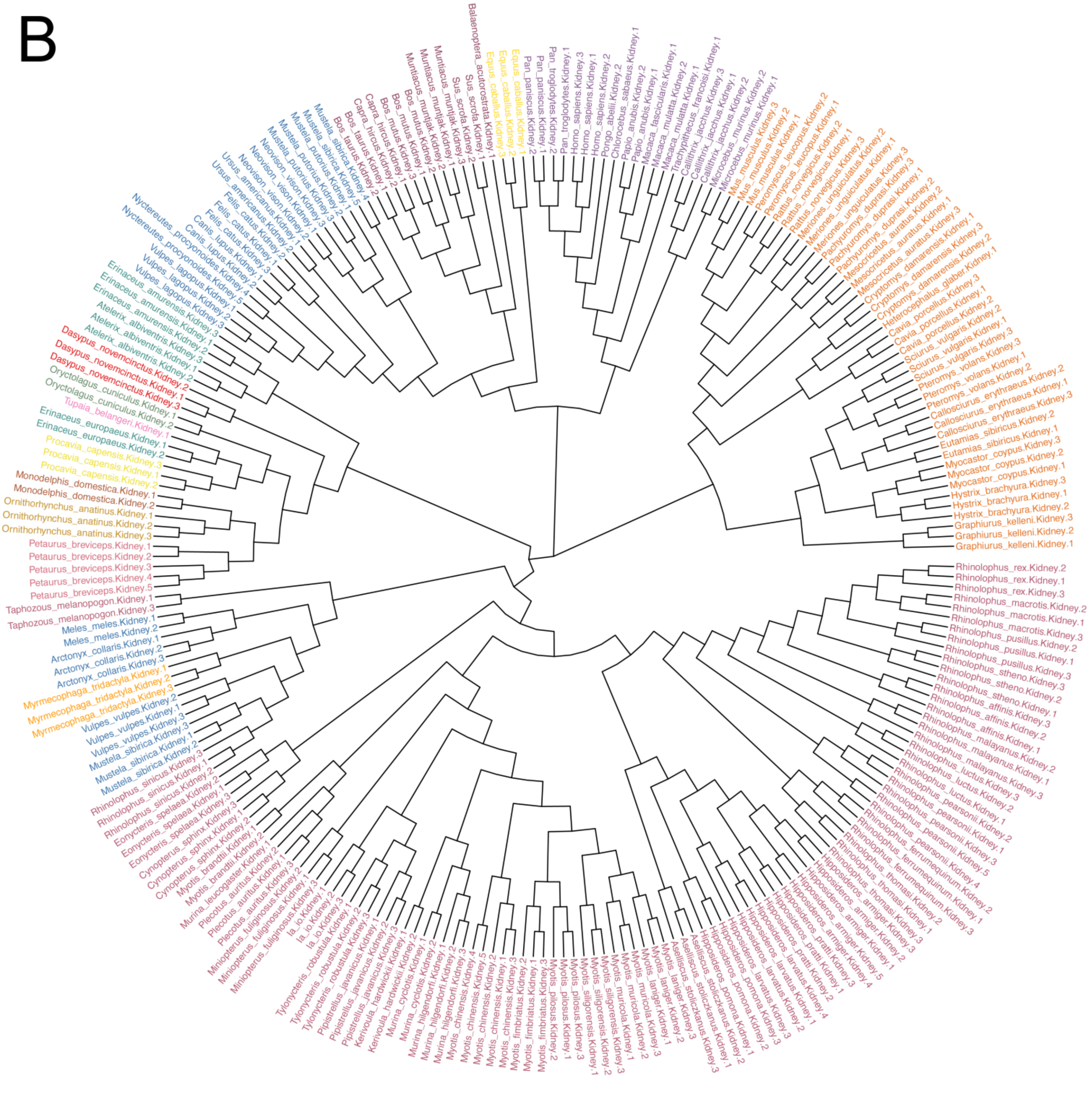

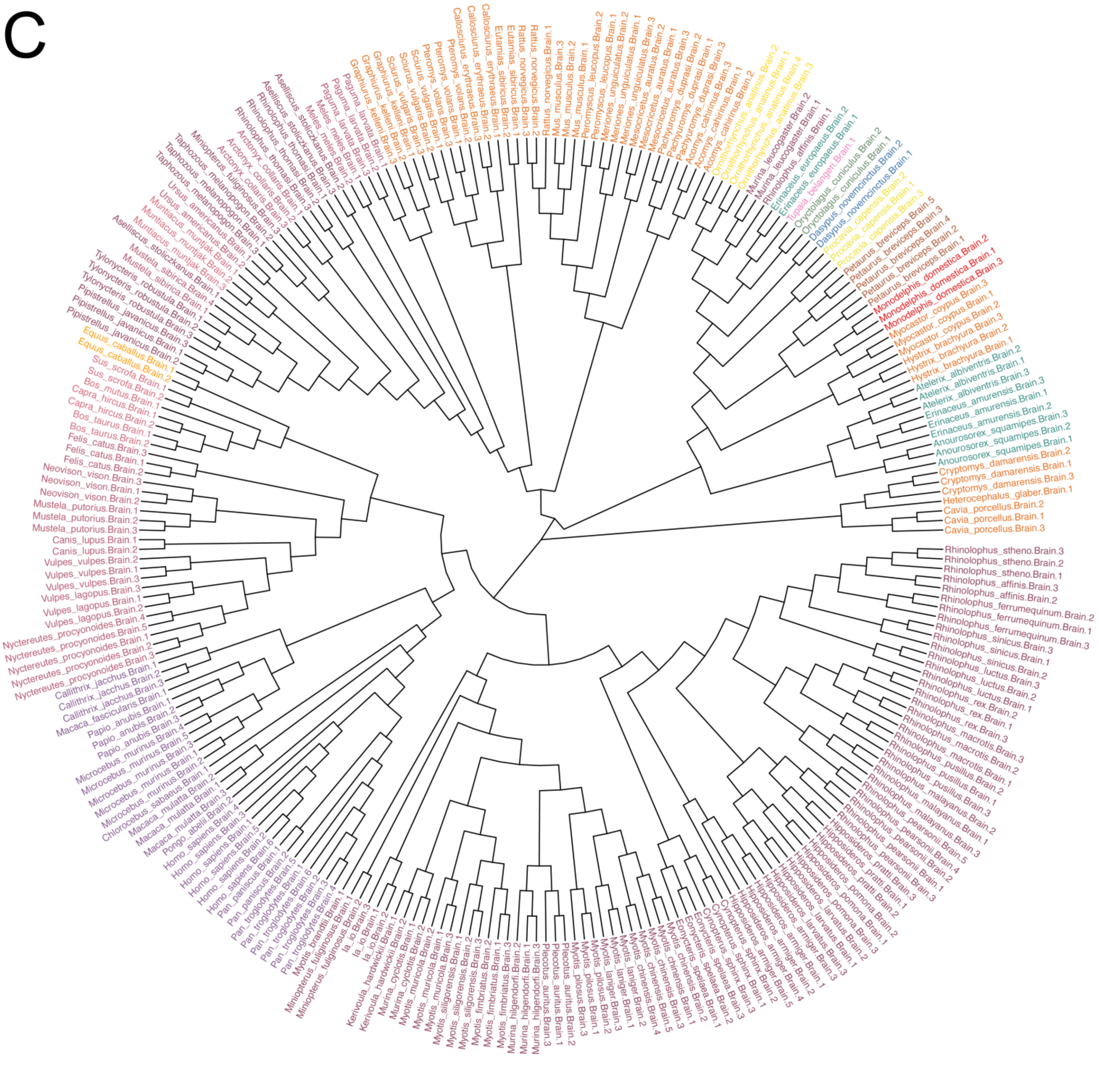

Appendix Fig S2 Sample clustering after outlier removal. The distance between samples is 1-Pearson correlation coefficient, and the tree is constructed by the neighbor-joining (NJ) method. (*A*) liver, (*B*) kidney and (*C*) brain.

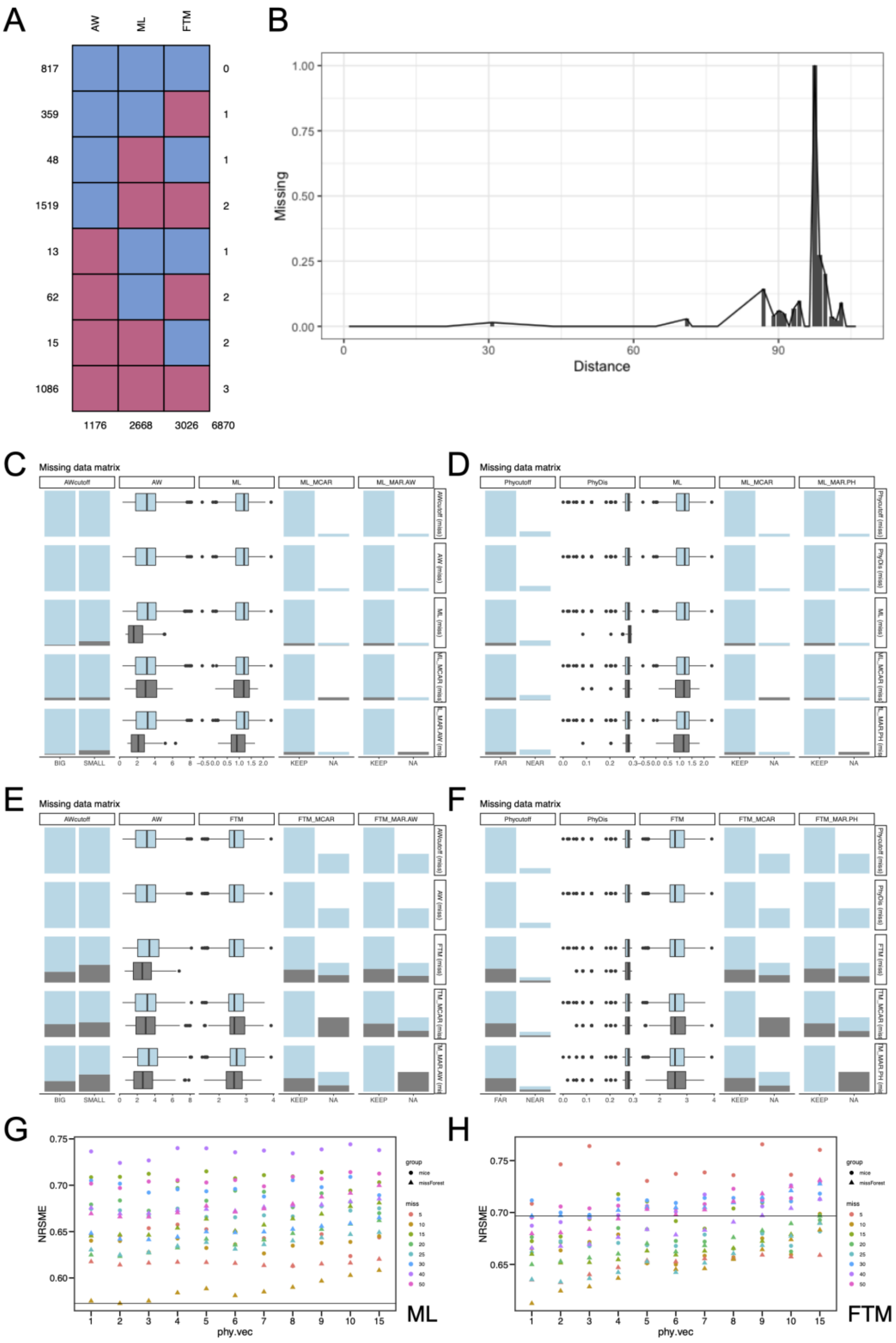

Appendix Fig S3 Life history data imputation. (*A*) Distribution of missing values in different life histories. (*B*) Distribution of missing values with increasing genetic distance from humans. (*C*) Correlation between missing value of maximum lifespan and adult weight. (*D*) Correlation between missing value of maximum life span and genetic distance from human. (*E*) Correlation between missing value of female time to maturity

and adult weight. (*F*) Correlation between missing value of female time to maturity and genetic distance from human. (*G*) For the maximum lifespan, the gradient estimation accuracy comparison of the number of phylogenetic vectors added in the model. The colors represent different proportions of missing values, and the shapes represent two imputation methods. (*H*) For the female time to maturity, the gradient estimation accuracy comparison of the number of phylogenetic vectors added in the model. The colors represent different proportions of missing values, and the shapes represent two imputation methods.

90

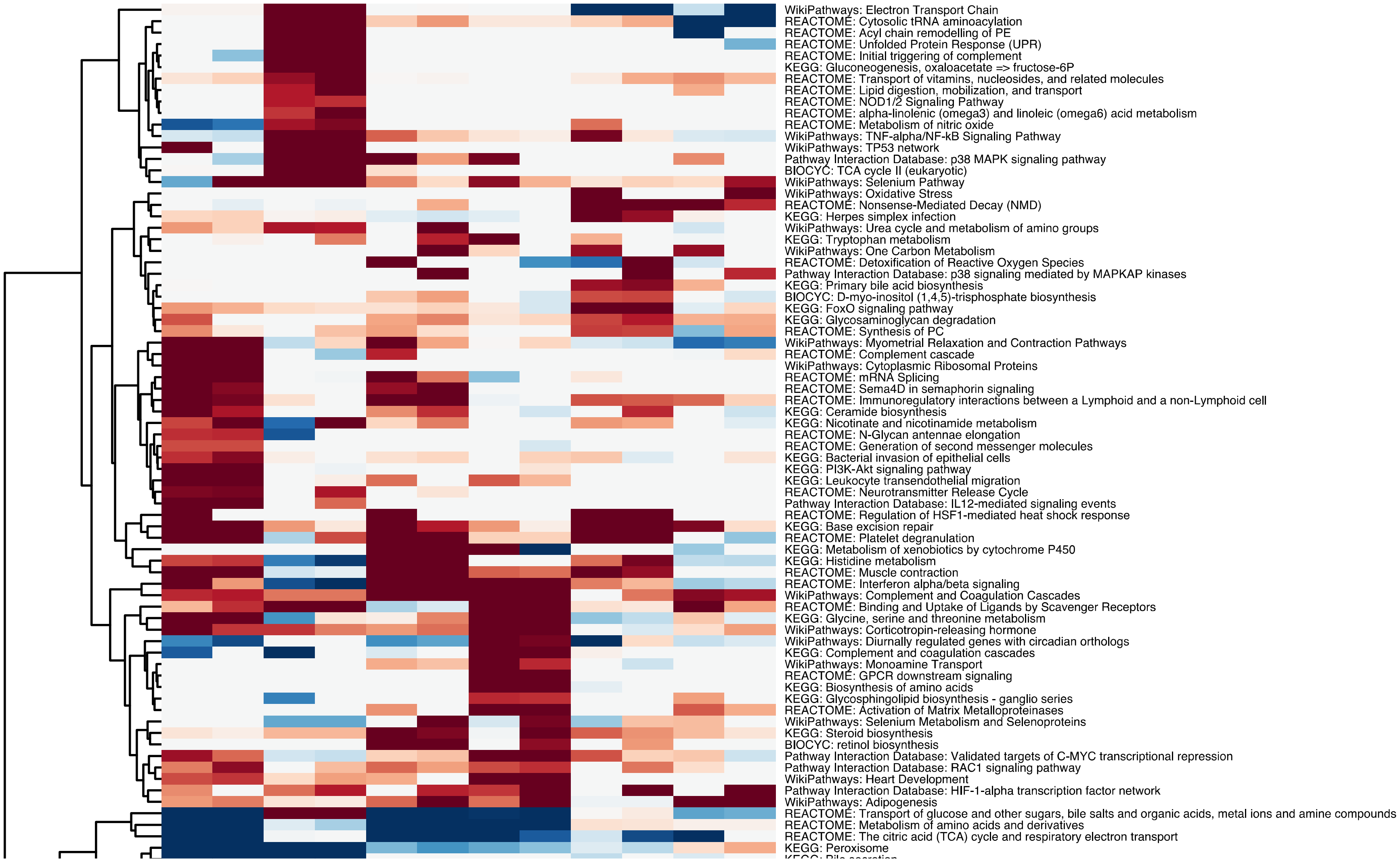

WikiPathways: Electron Transport Chain
REACTOME: Cytosolic tRNA aminoacylation
REACTOME: Acyl chain remodelling of PE
REACTOME: Unfolded Protein Response (UPR)
REACTOME: Initial triggering of complement
KEGG: Gluconeogenesis, oxaloacetate => fructose-6P
REACTOME: Transport of vitamins, nucleosides, and related molecules
REACTOME: Lipid digestion, mobilization, and transport
REACTOME: NOD1/2 Signaling Pathway
REACTOME: alpha-linolenic (omega3) and linoleic (omega6) acid metabolism
REACTOME: Metabolism of nitric oxide
WikiPathways: TNF-alpha/NF-kB Signaling Pathway
WikiPathways: TP53 network
Pathway Interaction Database: p38 MAPK signaling pathway
BIOCYC: TCA cycle II (eukaryotic)
WikiPathways: Selenium Pathway
WikiPathways: Oxidative Stress
REACTOME: Nonsense-Mediated Decay (NMD)
KEGG: Herpes simplex infection
WikiPathways: Urea cycle and metabolism of amino groups
KEGG: Tryptophan metabolism
WikiPathways: One Carbon Metabolism
REACTOME: Detoxification of Reactive Oxygen Species
Pathway Interaction Database: p38 signaling mediated by MAPKAP kinases
KEGG: Primary bile acid biosynthesis
BIOCYC: D-myo-inositol (1,4,5)-trisphosphate biosynthesis
KEGG: FoxO signaling pathway
KEGG: Glycosaminoglycan degradation
REACTOME: Synthesis of PC
WikiPathways: Myometrial Relaxation and Contraction Pathways
REACTOME: Complement cascade
WikiPathways: Cytoplasmic Ribosomal Proteins
REACTOME: mRNA Splicing
REACTOME: Sema4D in semaphorin signaling
REACTOME: Immunoregulatory interactions between a Lymphoid and a non-Lymphoid cell
KEGG: Ceramide biosynthesis
KEGG: Nicotinate and nicotinamide metabolism
REACTOME: N-Glycan antennae elongation
REACTOME: Generation of second messenger molecules
KEGG: Bacterial invasion of epithelial cells
KEGG: PI3K-Akt signaling pathway
KEGG: Leukocyte transendothelial migration
REACTOME: Neurotransmitter Release Cycle
Pathway Interaction Database: IL12-mediated signaling events
REACTOME: Regulation of HSF1-mediated heat shock response
KEGG: Base excision repair
REACTOME: Platelet degranulation
KEGG: Metabolism of xenobiotics by cytochrome P450
KEGG: Histidine metabolism
REACTOME: Muscle contraction
REACTOME: Interferon alpha/beta signaling
WikiPathways: Complement and Coagulation Cascades
REACTOME: Binding and Uptake of Ligands by Scavenger Receptors
KEGG: Glycine, serine and threonine metabolism
WikiPathways: Corticotropin-releasing hormone
WikiPathways: Diurnally regulated genes with circadian orthologs
KEGG: Complement and coagulation cascades
WikiPathways: Monoamine Transport
REACTOME: GPCR downstream signaling
KEGG: Biosynthesis of amino acids
KEGG: Glycosphingolipid biosynthesis - ganglio series
REACTOME: Activation of Matrix Metalloproteinases
WikiPathways: Selenium Metabolism and Selenoproteins
KEGG: Steroid biosynthesis
BIOCYC: retinol biosynthesis
Pathway Interaction Database: Validated targets of C-MYC transcriptional repression
Pathway Interaction Database: RAC1 signaling pathway
WikiPathways: Heart Development
Pathway Interaction Database: HIF-1-alpha transcription factor network
WikiPathways: Adipogenesis
REACTOME: Transport of glucose and other sugars, bile salts and organic acids, metal ions and amine compounds
REACTOME: Metabolism of amino acids and derivatives
REACTOME: The citric acid (TCA) cycle and respiratory electron transport
KEGG: Peroxisome

91

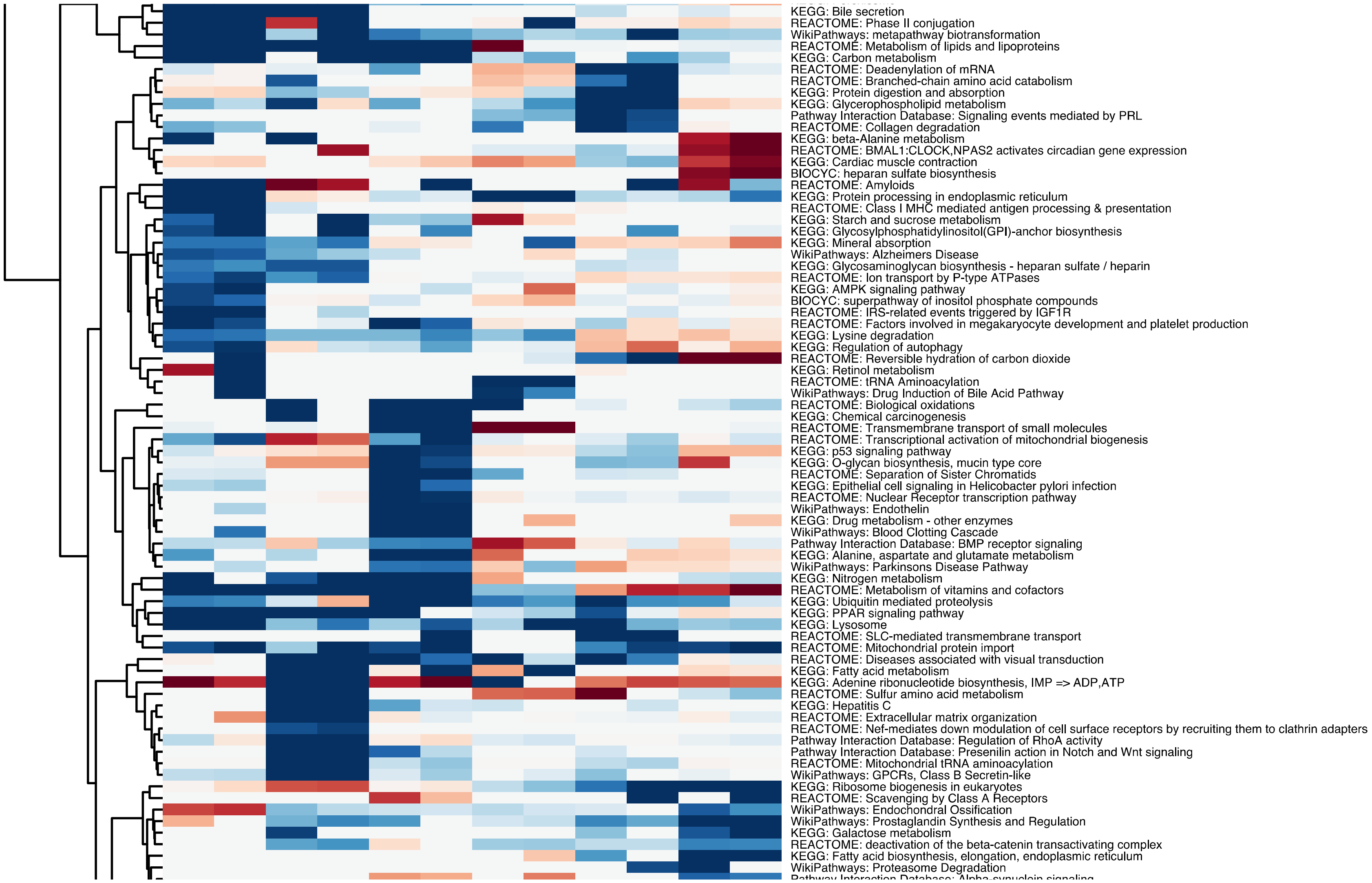
KEGG: Bile secretion
REACTOME: Phase II conjugation
WikiPathways: metapathway biotransformation
REACTOME: Metabolism of lipids and lipoproteins
KEGG: Carbon metabolism
REACTOME: Deadenylation of mRNA
REACTOME: Branched-chain amino acid catabolism
KEGG: Protein digestion and absorption
KEGG: Glycerophospholipid metabolism
Pathway Interaction Database: Signaling events mediated by PRL
REACTOME: Collagen degradation
KEGG: beta-Alanine metabolism
REACTOME: BMAL1:CLOCK,NPAS2 activates circadian gene expression
KEGG: Cardiac muscle contraction
BIOCYC: heparan sulfate biosynthesis
REACTOME: Amyloids
KEGG: Protein processing in endoplasmic reticulum
REACTOME: Class I MHC mediated antigen processing & presentation
KEGG: Starch and sucrose metabolism
KEGG: Glycosylphosphatidylinositol(GPI)-anchor biosynthesis
KEGG: Mineral absorption
WikiPathways: Alzheimers Disease
KEGG: Glycosaminoglycan biosynthesis - heparan sulfate / heparin
REACTOME: Ion transport by P-type ATPases
KEGG: AMPK signaling pathway
BIOCYC: superpathway of inositol phosphate compounds
REACTOME: IRS-related events triggered by IGF1R
REACTOME: Factors involved in megakaryocyte development and platelet production
KEGG: Lysine degradation
KEGG: Regulation of autophagy
REACTOME: Reversible hydration of carbon dioxide
KEGG: Retinol metabolism
REACTOME: tRNA Aminoacylation
WikiPathways: Drug Induction of Bile Acid Pathway
REACTOME: Biological oxidations
KEGG: Chemical carcinogenesis
REACTOME: Transmembrane transport of small molecules
REACTOME: Transcriptional activation of mitochondrial biogenesis
KEGG: p53 signaling pathway
KEGG: O-glycan biosynthesis, mucin type core
REACTOME: Separation of Sister Chromatids
KEGG: Epithelial cell signaling in Helicobacter pylori infection
REACTOME: Nuclear Receptor transcription pathway
WikiPathways: Endothelin
KEGG: Drug metabolism - other enzymes
WikiPathways: Blood Clotting Cascade
Pathway Interaction Database: BMP receptor signaling
KEGG: Alanine, aspartate and glutamate metabolism
WikiPathways: Parkinsons Disease Pathway
KEGG: Nitrogen metabolism
REACTOME: Metabolism of vitamins and cofactors
KEGG: Ubiquitin mediated proteolysis
KEGG: PPAR signaling pathway
KEGG: Lysosome
REACTOME: SLC-mediated transmembrane transport
REACTOME: Mitochondrial protein import
REACTOME: Diseases associated with visual transduction
KEGG: Fatty acid metabolism
KEGG: Adenine ribonucleotide biosynthesis, IMP => ADP,ATP
REACTOME: Sulfur amino acid metabolism
KEGG: Hepatitis C
REACTOME: Extracellular matrix organization
REACTOME: Nef-mediates down modulation of cell surface receptors by recruiting them to clathrin adapters
Pathway Interaction Database: Regulation of RhoA activity
Pathway Interaction Database: Presenilin action in Notch and Wnt signaling
REACTOME: Mitochondrial tRNA aminoacylation
WikiPathways: GPCRs, Class B Secretin-like
KEGG: Ribosome biogenesis in eukaryotes
REACTOME: Scavenging by Class A Receptors
WikiPathways: Endochondral Ossification
WikiPathways: Prostaglandin Synthesis and Regulation
KEGG: Galactose metabolism
REACTOME: deactivation of the beta-catenin transactivating complex
KEGG: Fatty acid biosynthesis, elongation, endoplasmic reticulum
WikiPathways: Proteasome Degradation

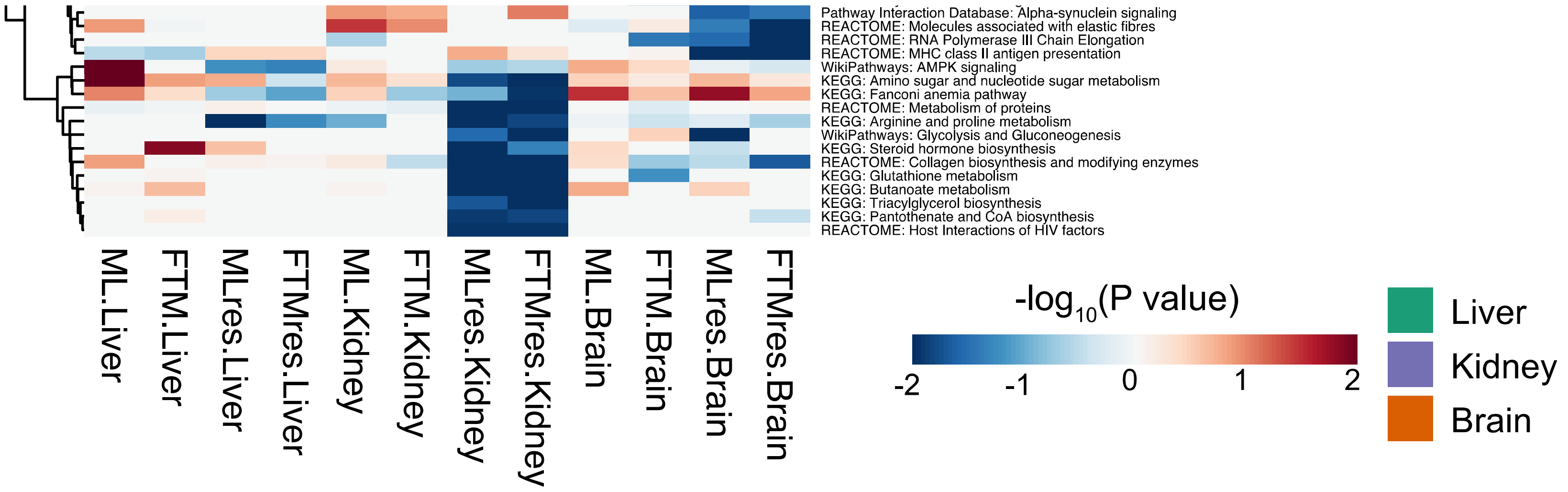

Appendix Fig S4 Gene set enrichment analysis heat map for all traits (postpruning). Represents a clustering heat map of pathways enriched by longevity-related genes and AW-related genes. The color intensity indicates the degree of significance, and the *P* value has undergone a -log10 transformation. Each row represents a different pathway, and each column represents related longevity traits (marked at the bottom). Among them, red is positive correlation, and blue is negative correlation.

Liver

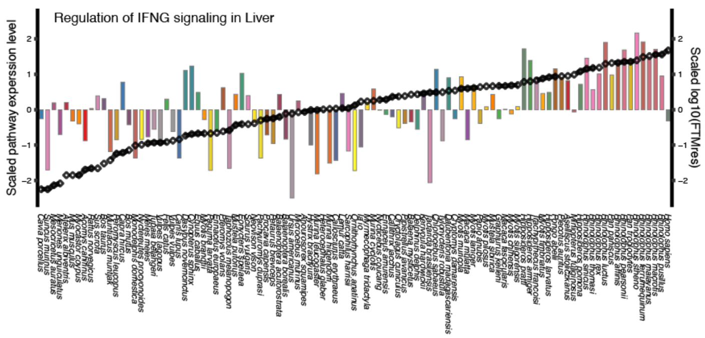

Regulation of IFNG signaling in Liver
Scaled pathway expression level
Scaled log10(FTMres)

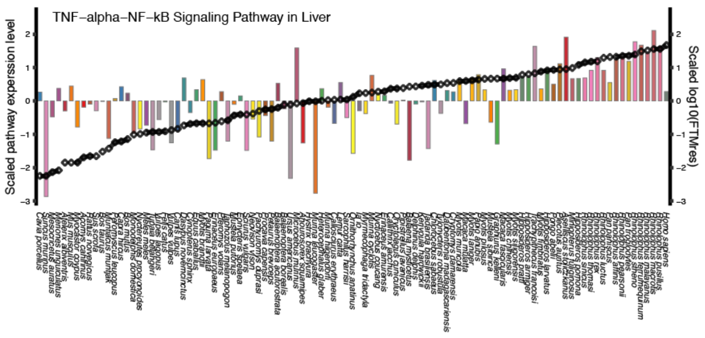

TNF−alpha−NF−kB Signaling Pathway in Liver
Scaled pathway expression level
Scaled log10(FTMres)

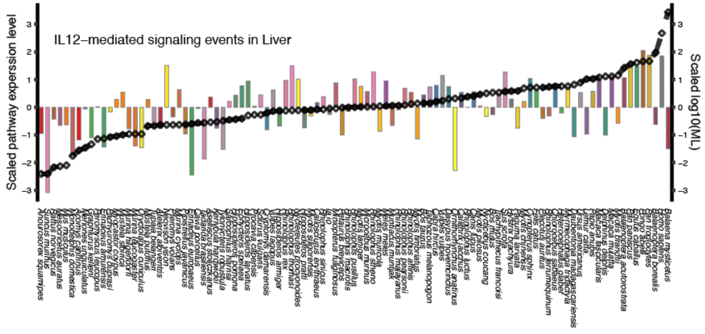

IL12−mediated signaling events in Liver
Scaled pathway expression level
Scaled log10(ML)

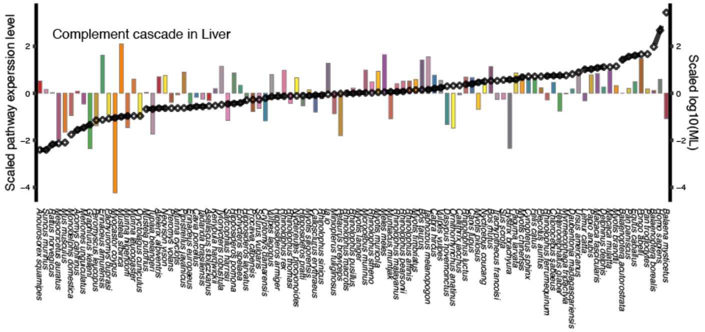

Complement cascade in Liver
Scaled pathway expression level
Scaled log10(ML)

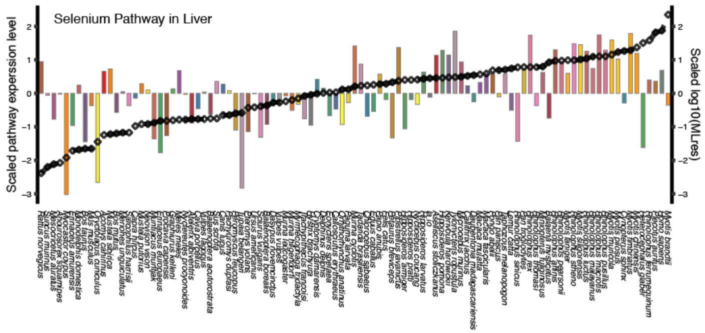

Selenium Pathway in Liver
Scaled pathway expression level
Scaled log10(MLres)

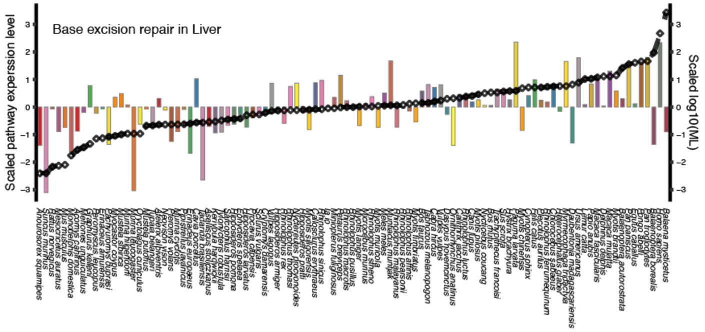

Base excision repair in Liver
Scaled pathway expression level
Scaled log10(ML)

97

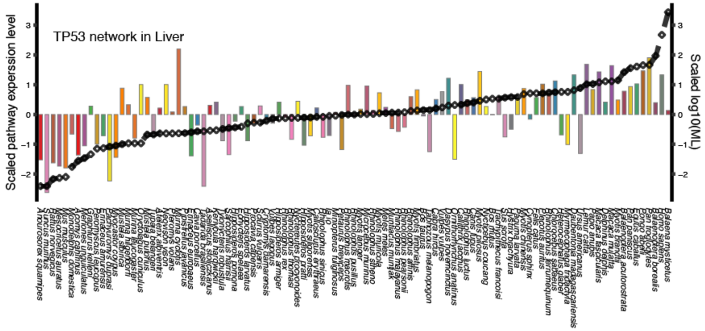

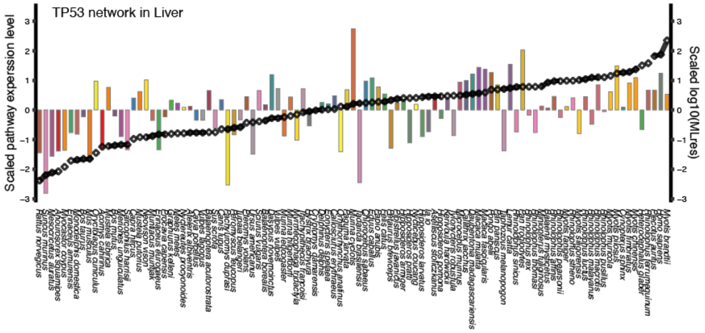

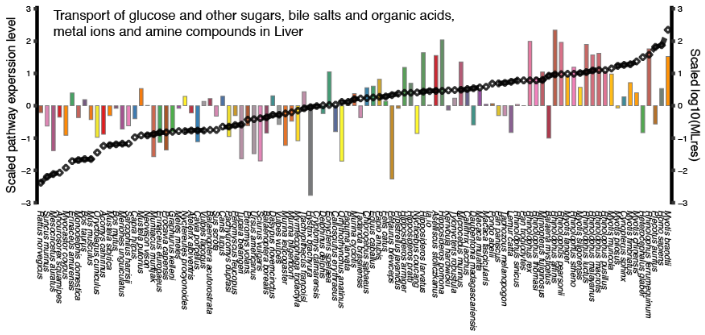

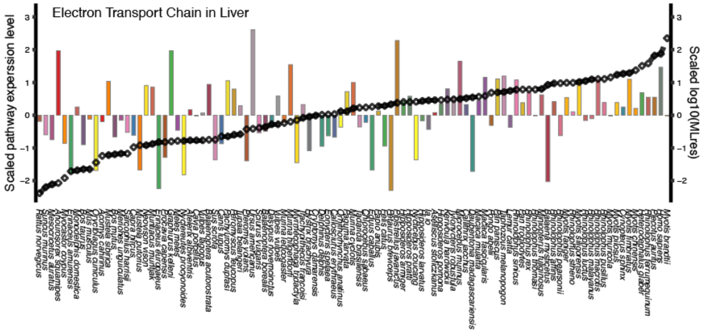

Postive

Negative

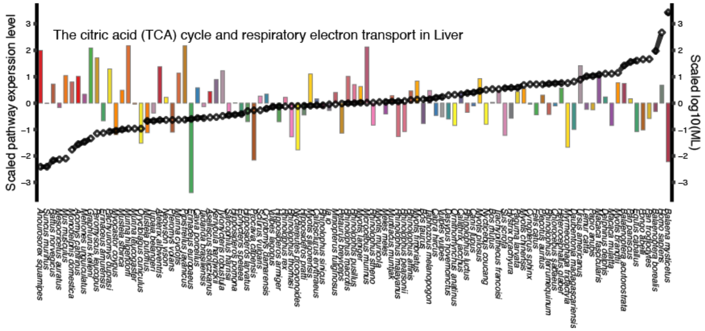

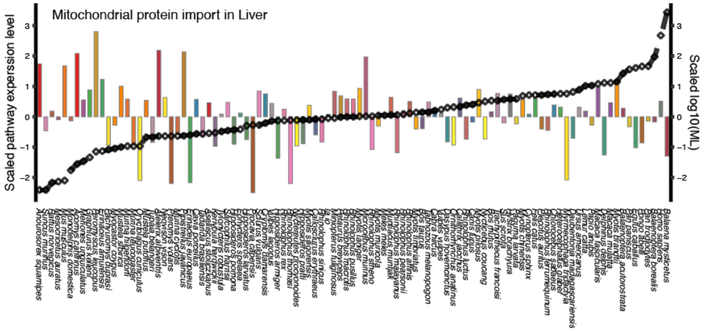

98

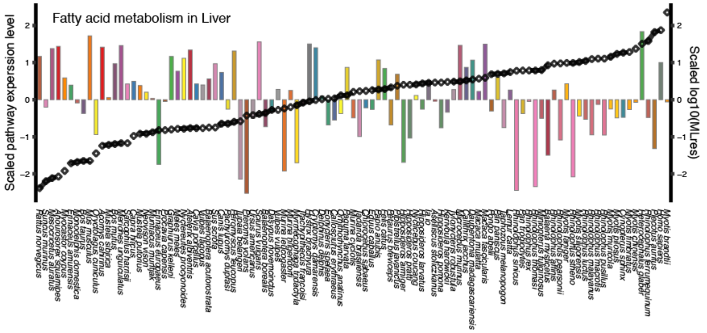

Appendix Fig S5 Top pathway for gene set enrichment in liver. The y-axis on the left represents the sum of the expression levels of all genes in the alas pathway of each species. The y-axis on the right represents the value of longevity-related traits are centered at 0 on log10 scale. The x-axis is the species name, and the ranking increases with the longevity-related traits. The upper part of the gray line is the positive correlation pathway, and the lower part is the negative correlation pathway.

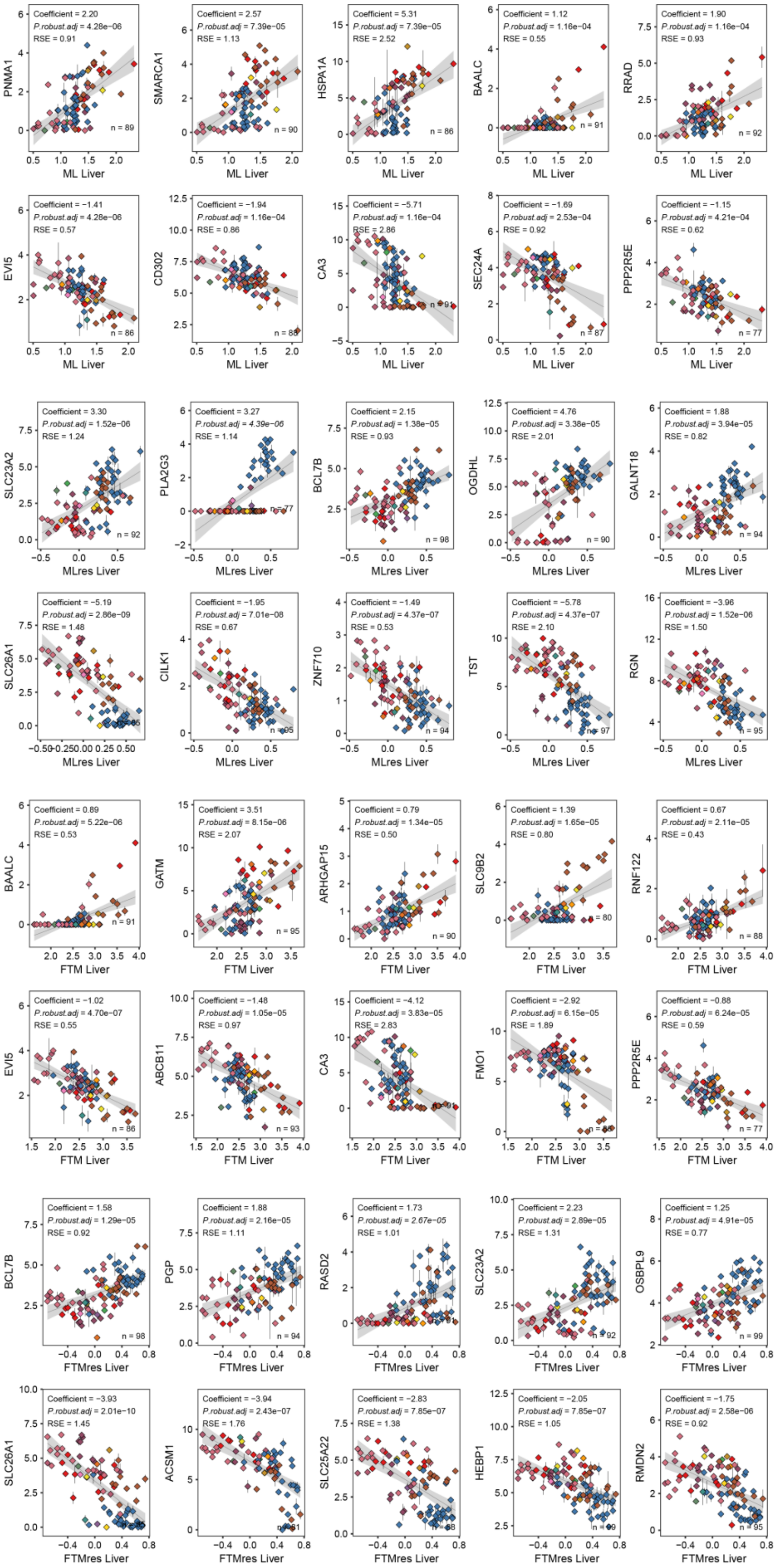
PNMA1 Coefficient = 2.20 P.robust.adj = 4.28e−06 RSE = 0.91 n = 89
SMARCA1 Coefficient = 2.57 P.robust.adj = 7.39e−05 RSE = 1.13 n = 90
HSPA1A Coefficient = 5.31 P.robust.adj = 7.39e−05 RSE = 2.52 n = 86
BAALC Coefficient = 1.12 P.robust.adj = 1.16e−04 RSE = 0.55 n = 91
RRAD Coefficient = 1.90 P.robust.adj = 1.16e−04 RSE = 0.93 n = 92
ML Liver
EVI5 Coefficient = −1.41 P.robust.adj = 4.28e−06 RSE = 0.57 n = 86
CD302 Coefficient = −1.94 P.robust.adj = 1.16e−04 RSE = 0.86 n = 88
CA3 Coefficient = −5.71 P.robust.adj = 1.16e−04 RSE = 2.86 n = 91
SEC24A Coefficient = −1.69 P.robust.adj = 2.53e−04 RSE = 0.92 n = 87
PPP2R5E Coefficient = −1.15 P.robust.adj = 4.21e−04 RSE = 0.62 n = 77
SLC23A2 Coefficient = 3.30 P.robust.adj = 1.52e−06 RSE = 1.24 n = 92
PLA2G3 Coefficient = 3.27 P.robust.adj = 4.39e−06 RSE = 1.14 n = 77
BCL7B Coefficient = 2.15 P.robust.adj = 1.38e−05 RSE = 0.93 n = 98
OGDHL Coefficient = 4.76 P.robust.adj = 3.38e−05 RSE = 2.01 n = 90
GALNT18 Coefficient = 1.88 P.robust.adj = 3.94e−05 RSE = 0.82 n = 94
MLres Liver
SLC26A1 Coefficient = −5.19 P.robust.adj = 2.86e−09 RSE = 1.48 n = 95
CILK1 Coefficient = −1.95 P.robust.adj = 7.01e−08 RSE = 0.67 n = 95
ZNF710 Coefficient = −1.49 P.robust.adj = 4.37e−07 RSE = 0.53 n = 94
TST Coefficient = −5.78 P.robust.adj = 4.37e−07 RSE = 2.10 n = 97
RGN Coefficient = −3.96 P.robust.adj = 1.52e−06 RSE = 1.50 n = 95
BAALC Coefficient = 0.89 P.robust.adj = 5.22e−06 RSE = 0.53 n = 91
GATM Coefficient = 3.51 P.robust.adj = 8.15e−06 RSE = 2.07 n = 95
ARHGAP15 Coefficient = 0.79 P.robust.adj = 1.34e−05 RSE = 0.50 n = 90
SLC9B2 Coefficient = 1.39 P.robust.adj = 1.65e−05 RSE = 0.80 n = 80
RNF122 Coefficient = 0.67 P.robust.adj = 2.11e−05 RSE = 0.43 n = 88
FTM Liver
EVI5 Coefficient = −1.02 P.robust.adj = 4.70e−07 RSE = 0.55 n = 86
ABCB11 Coefficient = −1.48 P.robust.adj = 1.05e−05 RSE = 0.97 n = 93
CA3 Coefficient = −4.12 P.robust.adj = 3.83e−05 RSE = 2.83
FMO1 Coefficient = −2.92 P.robust.adj = 6.15e−05 RSE = 1.89
PPP2R5E Coefficient = −0.88 P.robust.adj = 6.24e−05 RSE = 0.59 n = 77
BCL7B Coefficient = 1.58 P.robust.adj = 1.29e−05 RSE = 0.92 n = 98
PGP Coefficient = 1.88 P.robust.adj = 2.16e−05 RSE = 1.11 n = 94
RASD2 Coefficient = 1.73 P.robust.adj = 2.67e−05 RSE = 1.01
SLC23A2 Coefficient = 2.23 P.robust.adj = 2.89e−05 RSE = 1.31 n = 92
OSBPL9 Coefficient = 1.25 P.robust.adj = 4.91e−05 RSE = 0.77 n = 99
FTMres Liver
SLC26A1 Coefficient = −3.93 P.robust.adj = 2.01e−10 RSE = 1.45
ACSM1 Coefficient = −3.94 P.robust.adj = 2.43e−07 RSE = 1.76
SLC25A22 Coefficient = −2.83 P.robust.adj = 7.85e−07 RSE = 1.38
HEBP1 Coefficient = −2.05 P.robust.adj = 7.85e−07 RSE = 1.05
RMDN2 Coefficient = −1.75 P.robust.adj = 2.58e−06 RSE = 0.92 n = 95

Appendix Fig S6 Top five up/down-regulated genes with significant correlation to longevity in liver. In each figure, the y-axis is the scaled expression level of each gene with 0 as the center, and the x-axis is the longevity traits (ML: maximum lifespan; FTM: female time to maturity; MLres and FTMres: ML and FTM residuals adjusted for adult weight, respectively). Error bars represents SE. Potential outliers have been removed. The coefficient of phylogenetic regression, adj.P.robust and residual standard error (RSE) is included in the figure.

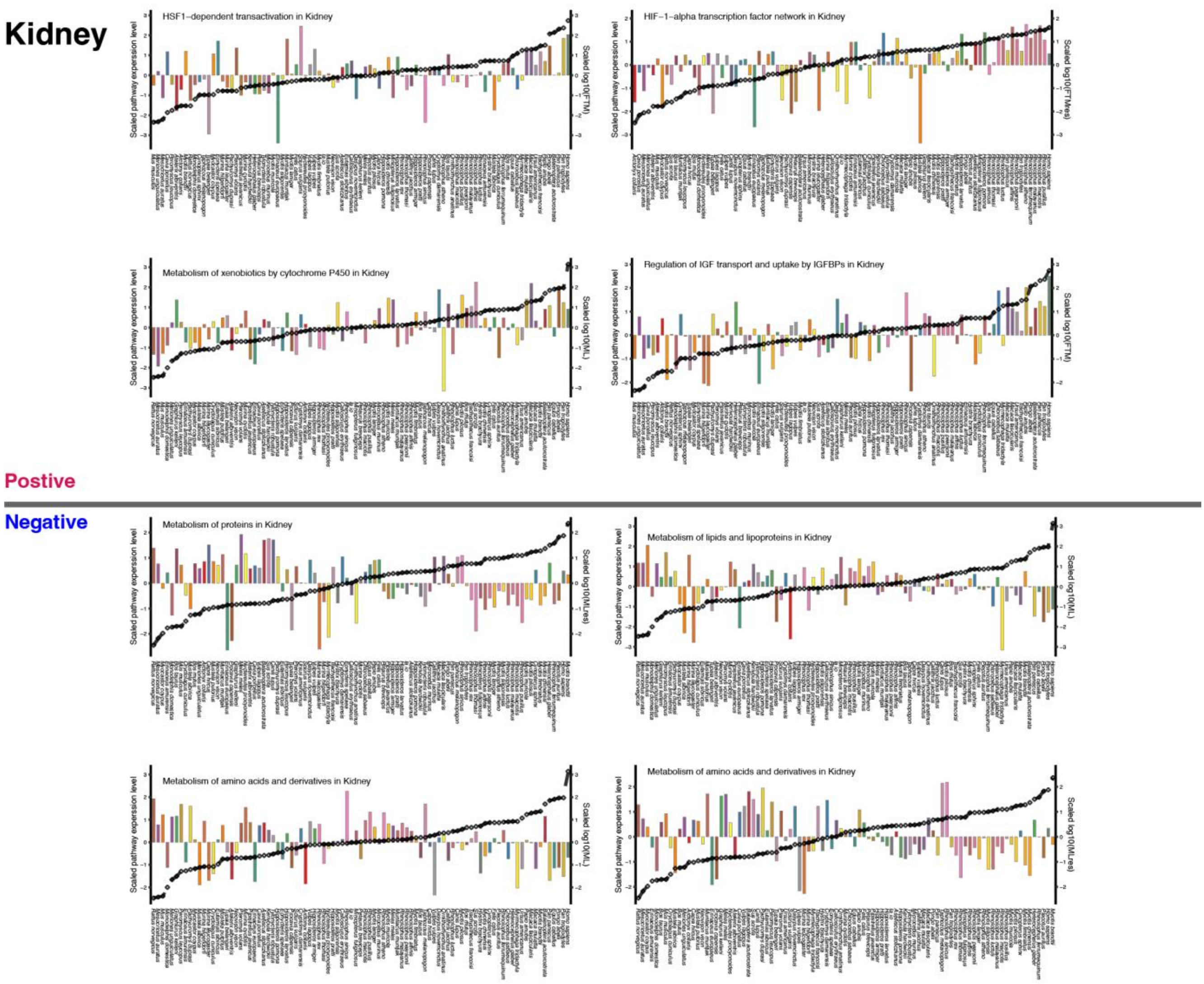

Appendix Fig S7 Top pathway for gene set enrichment in kidney. The y-axis on the left represents the sum of the expression levels of all genes in the alas pathway of each species. The y-axis on the right represents the value of longevity-related traits are centered at 0 on log10 scale. The x-axis is the species name, and the ranking increases with the longevity-related traits. The upper part of the gray line is the positive correlation pathway, and the lower part is the negative correlation pathway.

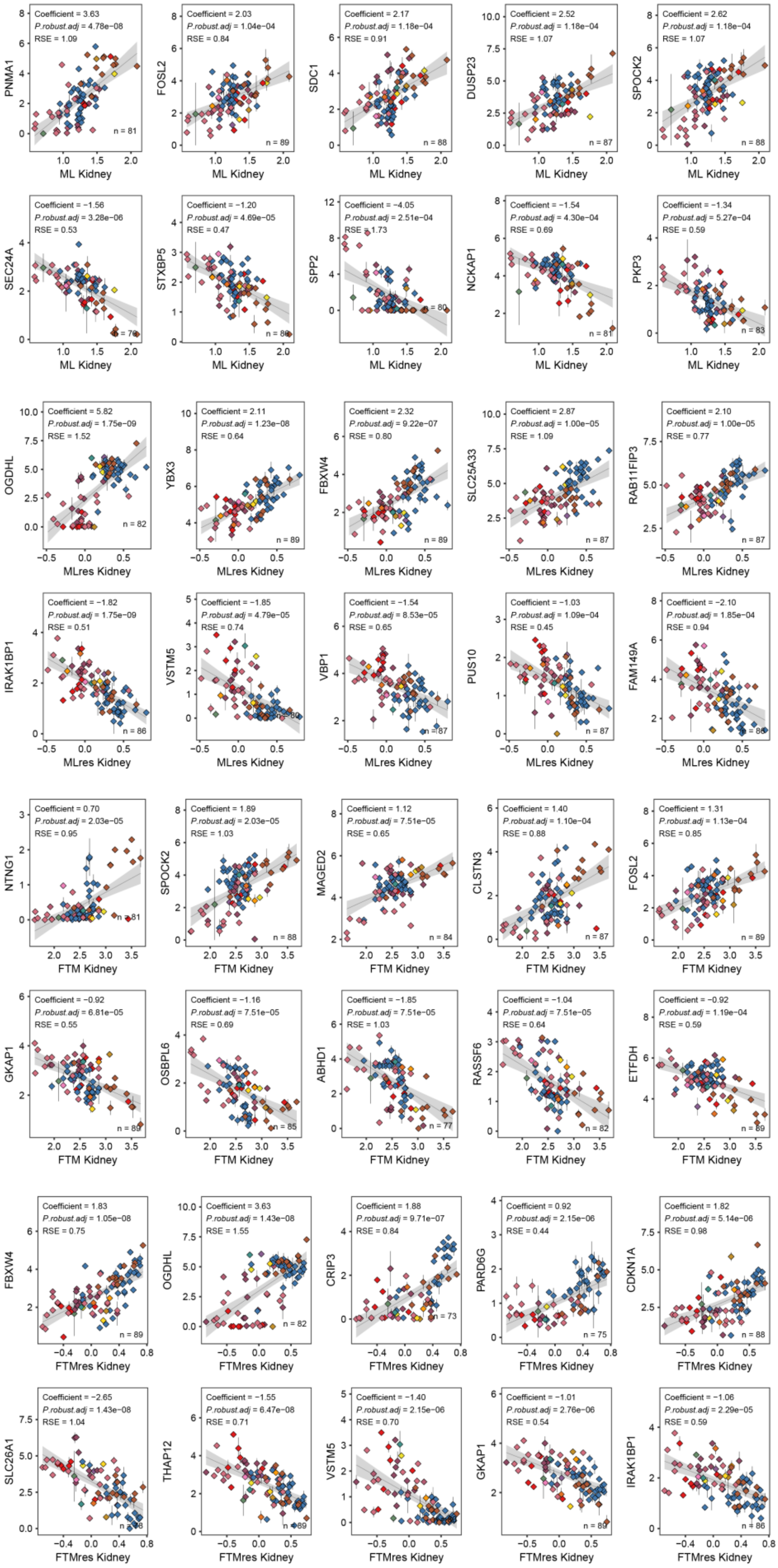
PNMA1 Coefficient = 3.63 P.robust.adj = 4.78e−08 RSE = 1.09 n = 81 ML Kidney
FOSL2 Coefficient = 2.03 P.robust.adj = 1.04e−04 RSE = 0.84 n = 89 ML Kidney
SDC1 Coefficient = 2.17 P.robust.adj = 1.18e−04 RSE = 0.91 n = 88 ML Kidney
DUSP23 Coefficient = 2.52 P.robust.adj = 1.18e−04 RSE = 1.07 n = 87 ML Kidney
SPOCK2 Coefficient = 2.62 P.robust.adj = 1.18e−04 RSE = 1.07 n = 88 ML Kidney
SEC24A Coefficient = −1.56 P.robust.adj = 3.28e−06 RSE = 0.53 ML Kidney
STXBP5 Coefficient = −1.20 P.robust.adj = 4.69e−05 RSE = 0.47 ML Kidney
SPP2 Coefficient = −4.05 P.robust.adj = 2.51e−04 RSE = 1.73 ML Kidney
NCKAP1 Coefficient = −1.54 P.robust.adj = 4.30e−04 RSE = 0.69 n = 81 ML Kidney
PKP3 Coefficient = −1.34 P.robust.adj = 5.27e−04 RSE = 0.59 n = 83 ML Kidney
OGDHL Coefficient = 5.82 P.robust.adj = 1.75e−09 RSE = 1.52 n = 82 MLres Kidney
YBX3 Coefficient = 2.11 P.robust.adj = 1.23e−08 RSE = 0.64 n = 89 MLres Kidney
FBXW4 Coefficient = 2.32 P.robust.adj = 9.22e−07 RSE = 0.80 n = 89 MLres Kidney
SLC25A33 Coefficient = 2.87 P.robust.adj = 1.00e−05 RSE = 1.09 n = 87 MLres Kidney
RAB11FIP3 Coefficient = 2.10 P.robust.adj = 1.00e−05 RSE = 0.77 n = 87 MLres Kidney
IRAK1BP1 Coefficient = −1.82 P.robust.adj = 1.75e−09 RSE = 0.51 n = 86 MLres Kidney
VSTM5 Coefficient = −1.85 P.robust.adj = 4.79e−05 RSE = 0.74 MLres Kidney
VBP1 Coefficient = −1.54 P.robust.adj = 8.53e−05 RSE = 0.65 MLres Kidney
PUS10 Coefficient = −1.03 P.robust.adj = 1.09e−04 RSE = 0.45 n = 87 MLres Kidney
FAM149A Coefficient = −2.10 P.robust.adj = 1.85e−04 RSE = 0.94 MLres Kidney
NTNG1 Coefficient = 0.70 P.robust.adj = 2.03e−05 RSE = 0.95 FTM Kidney
SPOCK2 Coefficient = 1.89 P.robust.adj = 2.03e−05 RSE = 1.03 n = 88 FTM Kidney
MAGED2 Coefficient = 1.12 P.robust.adj = 7.51e−05 RSE = 0.65 n = 84 FTM Kidney
CLSTN3 Coefficient = 1.40 P.robust.adj = 1.10e−04 RSE = 0.88 n = 87 FTM Kidney
FOSL2 Coefficient = 1.31 P.robust.adj = 1.13e−04 RSE = 0.85 n = 89 FTM Kidney
GKAP1 Coefficient = −0.92 P.robust.adj = 6.81e−05 RSE = 0.55 FTM Kidney
OSBPL6 Coefficient = −1.16 P.robust.adj = 7.51e−05 RSE = 0.69 n = 85 FTM Kidney
ABHD1 Coefficient = −1.85 P.robust.adj = 7.51e−05 RSE = 1.03 n = 77 FTM Kidney
RASSF6 Coefficient = −1.04 P.robust.adj = 7.51e−05 RSE = 0.64 n = 82 FTM Kidney
ETFDH Coefficient = −0.92 P.robust.adj = 1.19e−04 RSE = 0.59 FTM Kidney
FBXW4 Coefficient = 1.83 P.robust.adj = 1.05e−08 RSE = 0.75 n = 89 FTMres Kidney
OGDHL Coefficient = 3.63 P.robust.adj = 1.43e−08 RSE = 1.55 n = 82 FTMres Kidney
CRIP3 Coefficient = 1.88 P.robust.adj = 9.71e−07 RSE = 0.84 n = 73 FTMres Kidney
PARD6G Coefficient = 0.92 P.robust.adj = 2.15e−06 RSE = 0.44 n = 75 FTMres Kidney
CDKN1A Coefficient = 1.82 P.robust.adj = 5.14e−06 RSE = 0.98 n = 88 FTMres Kidney
SLC26A1 Coefficient = −2.65 P.robust.adj = 1.43e−08 RSE = 1.04 FTMres Kidney
THAP12 Coefficient = −1.55 P.robust.adj = 6.47e−08 RSE = 0.71 FTMres Kidney
VSTM5 Coefficient = −1.40 P.robust.adj = 2.15e−06 RSE = 0.70 FTMres Kidney
GKAP1 Coefficient = −1.01 P.robust.adj = 2.76e−06 RSE = 0.54 FTMres Kidney
IRAK1BP1 Coefficient = −1.06 P.robust.adj = 2.29e−05 RSE = 0.59 n = 86 FTMres Kidney

Appendix Fig S8 Top five up/down-regulated genes with significant correlation to longevity in kidney. In each figure, the y-axis is the scaled expression level of each gene with 0 as the center, and the x-axis is the longevity traits (ML: maximum lifespan; FTM: female time to maturity; MLres and FTMres: ML and FTM residuals adjusted for adult weight, respectively). Error bars represents SE. Potential outliers have been removed. The coefficient of phylogenetic regression, adj.P.robust and residual standard error (RSE) is included in the figure.

128

Brain

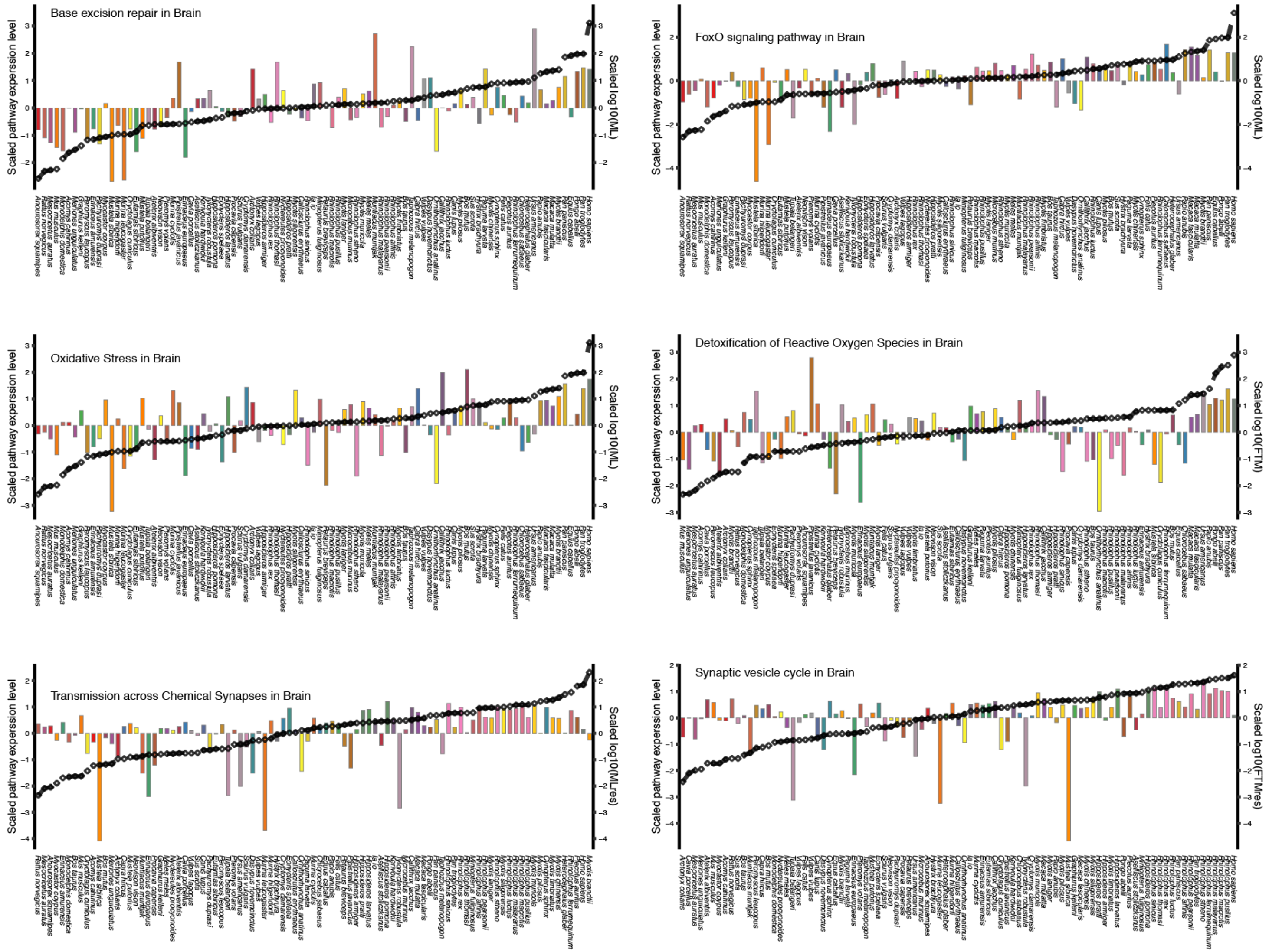

Postive

129 **Negative**

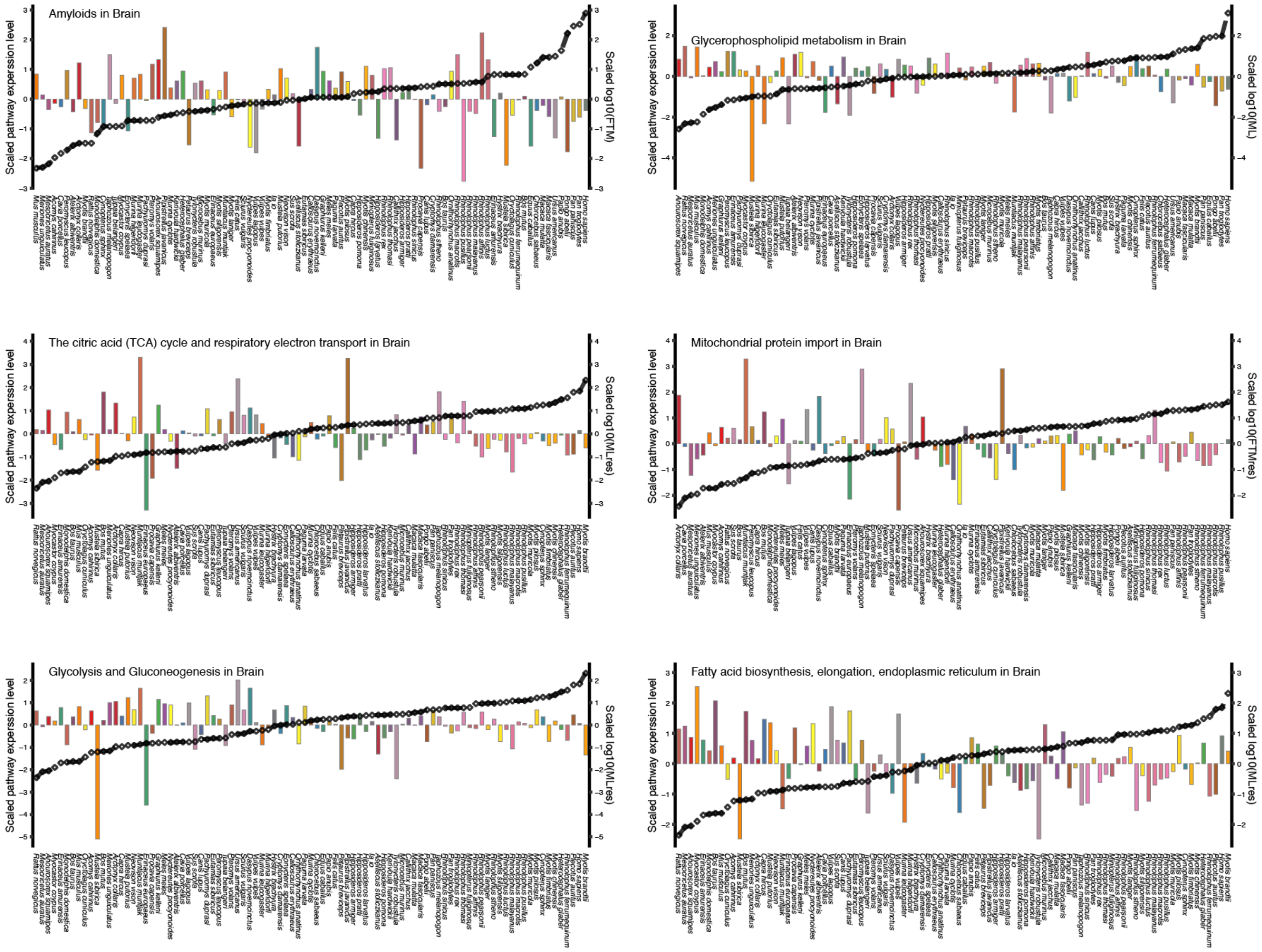

Appendix Fig S9 Top pathway for gene set enrichment in brain. The y-axis on the left represents the sum of the expression levels of all genes in the alas pathway of each species. The y-axis on the right represents the value of longevity-related traits are centered at 0 on log10 scale. The x-axis is the species name, and the ranking increases with the longevity-related traits. The upper part of the gray line is the positive correlation pathway, and the lower part is the negative correlation pathway.

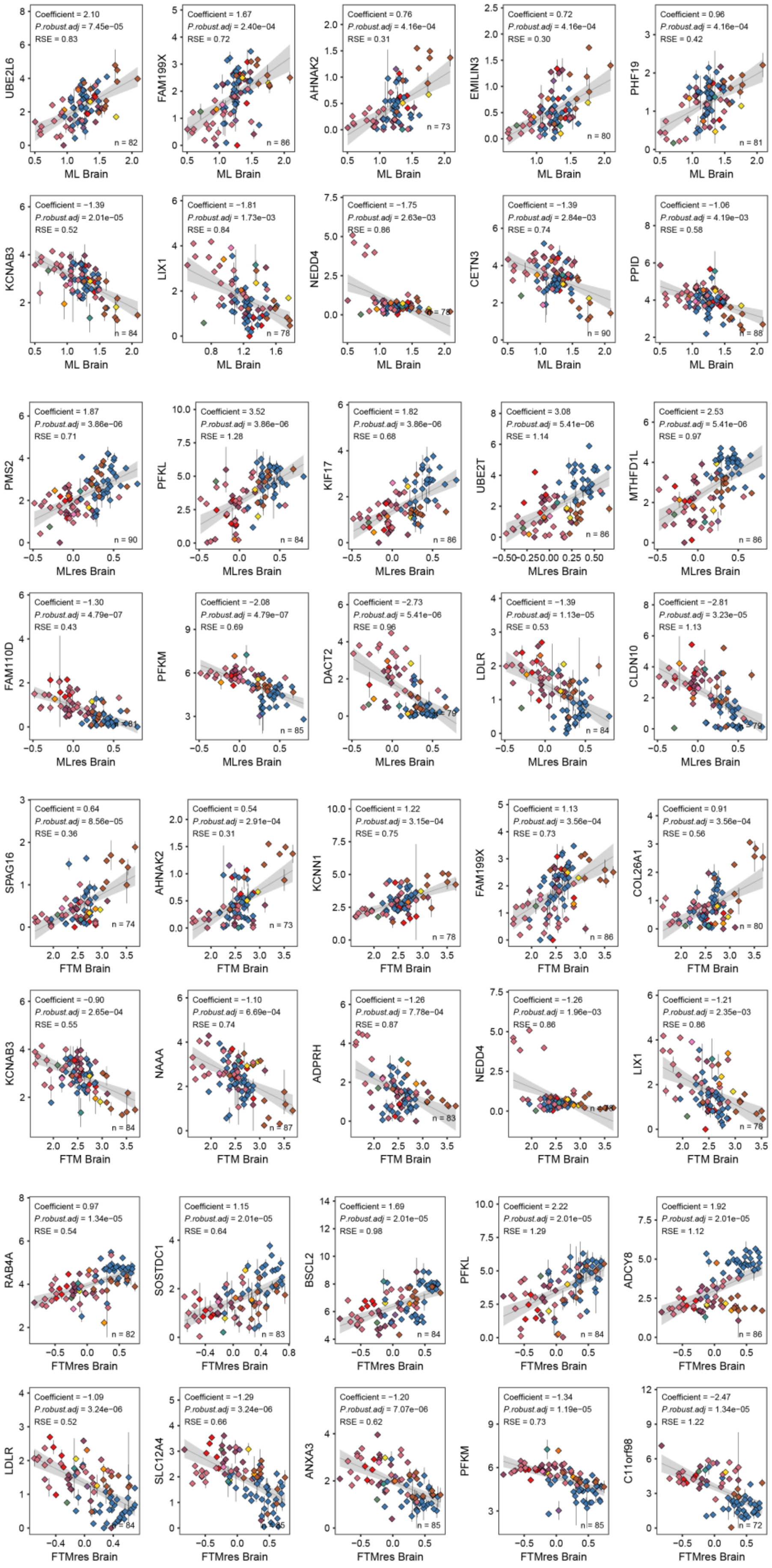
UBE2L6
Coefficient = 2.10
P.robust.adj = 7.45e−05
RSE = 0.83
n = 82
FAM199X
Coefficient = 1.67
P.robust.adj = 2.40e−04
RSE = 0.72
n = 86
AHNAK2
Coefficient = 0.76
P.robust.adj = 4.16e−04
RSE = 0.31
n = 73
EMILIN3
Coefficient = 0.72
P.robust.adj = 4.16e−04
RSE = 0.30
n = 80
PHF19
Coefficient = 0.96
P.robust.adj = 4.16e−04
RSE = 0.42
n = 81
KCNAB3
Coefficient = −1.39
P.robust.adj = 2.01e−05
RSE = 0.52
n = 84
LIX1
Coefficient = −1.81
P.robust.adj = 1.73e−03
RSE = 0.84
n = 78
NEDD4
Coefficient = −1.75
P.robust.adj = 2.63e−03
RSE = 0.86
CETN3
Coefficient = −1.39
P.robust.adj = 2.84e−03
RSE = 0.74
n = 90
PPID
Coefficient = −1.06
P.robust.adj = 4.19e−03
RSE = 0.58
n = 88
ML Brain
PMS2
Coefficient = 1.87
P.robust.adj = 3.86e−06
RSE = 0.71
n = 90
PFKL
Coefficient = 3.52
P.robust.adj = 3.86e−06
RSE = 1.28
n = 84
KIF17
Coefficient = 1.82
P.robust.adj = 3.86e−06
RSE = 0.68
n = 86
UBE2T
Coefficient = 3.08
P.robust.adj = 5.41e−06
RSE = 1.14
n = 86
MTHFD1L
Coefficient = 2.53
P.robust.adj = 5.41e−06
RSE = 0.97
n = 86
FAM110D
Coefficient = −1.30
P.robust.adj = 4.79e−07
RSE = 0.43
PFKM
Coefficient = −2.08
P.robust.adj = 4.79e−07
RSE = 0.69
n = 85
DACT2
Coefficient = −2.73
P.robust.adj = 5.41e−06
RSE = 0.96
LDLR
Coefficient = −1.39
P.robust.adj = 1.13e−05
RSE = 0.53
n = 84
CLDN10
Coefficient = −2.81
P.robust.adj = 3.23e−05
RSE = 1.13
MLres Brain
SPAG16
Coefficient = 0.64
P.robust.adj = 8.56e−05
RSE = 0.36
n = 74
AHNAK2
Coefficient = 0.54
P.robust.adj = 2.91e−04
RSE = 0.31
n = 73
KCNN1
Coefficient = 1.22
P.robust.adj = 3.15e−04
RSE = 0.75
n = 78
FAM199X
Coefficient = 1.13
P.robust.adj = 3.56e−04
RSE = 0.73
n = 86
COL26A1
Coefficient = 0.91
P.robust.adj = 3.56e−04
RSE = 0.56
n = 80
KCNAB3
Coefficient = −0.90
P.robust.adj = 2.65e−04
RSE = 0.55
n = 84
NAAA
Coefficient = −1.10
P.robust.adj = 6.69e−04
RSE = 0.74
n = 87
ADPRH
Coefficient = −1.26
P.robust.adj = 7.78e−04
RSE = 0.87
n = 83
NEDD4
Coefficient = −1.26
P.robust.adj = 1.96e−03
RSE = 0.86
LIX1
Coefficient = −1.21
P.robust.adj = 2.35e−03
RSE = 0.86
n = 78
FTM Brain
RAB4A
Coefficient = 0.97
P.robust.adj = 1.34e−05
RSE = 0.54
n = 82
SOSTDC1
Coefficient = 1.15
P.robust.adj = 2.01e−05
RSE = 0.64
n = 83
BSCL2
Coefficient = 1.69
P.robust.adj = 2.01e−05
RSE = 0.98
n = 84
PFKL
Coefficient = 2.22
P.robust.adj = 2.01e−05
RSE = 1.29
n = 84
ADCY8
Coefficient = 1.92
P.robust.adj = 2.01e−05
RSE = 1.12
n = 86
LDLR
Coefficient = −1.09
P.robust.adj = 3.24e−06
RSE = 0.52
SLC12A4
Coefficient = −1.29
P.robust.adj = 3.24e−06
RSE = 0.66
ANXA3
Coefficient = −1.20
P.robust.adj = 7.07e−06
RSE = 0.62
n = 85
PFKM
Coefficient = −1.34
P.robust.adj = 1.19e−05
RSE = 0.73
n = 85
C11orf98
Coefficient = −2.47
P.robust.adj = 1.34e−05
RSE = 1.22
n = 72
FTMres Brain

Appendix Fig S10 Top five up/down-regulated genes with significant correlation to longevity in brain. In each figure, the y-axis is the scaled expression level of each gene with 0 as the center, and the x-axis is the longevity traits (ML: maximum lifespan; FTM: female time to maturity; MLres and FTMres: ML and FTM residuals adjusted for adult weight, respectively). Error bars represents SE. Potential outliers have been removed. The coefficient of phylogenetic regression, adj.P.robust and residual standard error (RSE) is included in the figure.

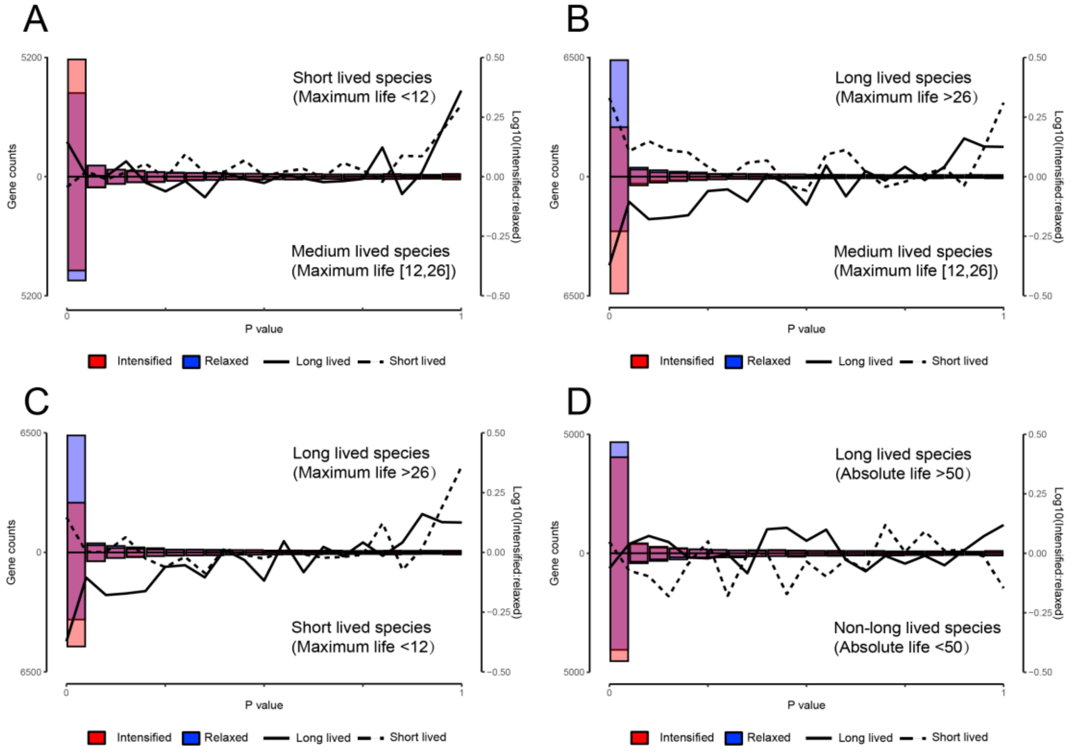

Appendix Fig S11 Relaxation of Selection in different lifespan interval

P value distributions of the RELAX tests of long-lived species (ML > 26 or ML > 50), medium-lived species (12 <= ML <= 26), short-lived species (ML < 12) and non-long-lived species (ML < 50). (*A*) Contrast between short-lived species and medium-lived species, (*B*) Contrast between long-lived species and medium-lived species, (*C*) Contrast between long-lived species and short-lived species, and (*D*) Contrast between long-lived species and non-long-lived species are shown. Blue bars, genes under relaxed selection (k < 1); red bars, intensified genes (k > 1). Dotted (short-lived) and solid (long-lived) lines are the log ratios of k > 1: k < 1 gene counts.

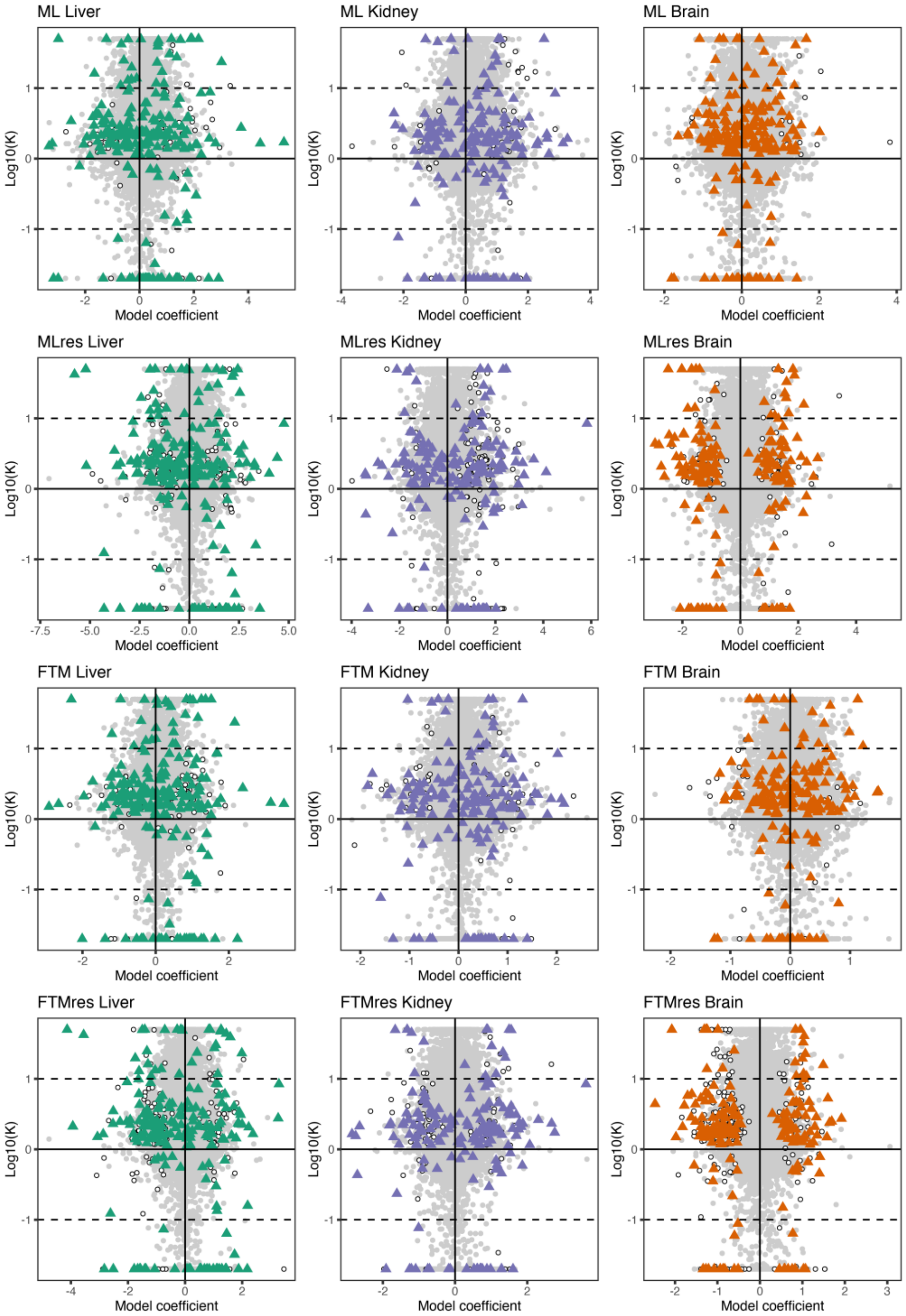

Appendix Fig S12 The relationship between the index of relaxed choice (K) and the regression coefficient. Scatter plot showing the log10-transformed relaxation parameter (k) on the y-axis, and the variation rate of gene expression along the longevity trait gradient on the x-axis. The colored points represent longevity-related genes (the color corresponds to the tissue type shown in Fig.4B), white point represent genes that are only significant related to this trait and the grey points represent genes that are only significant in RLEAX.

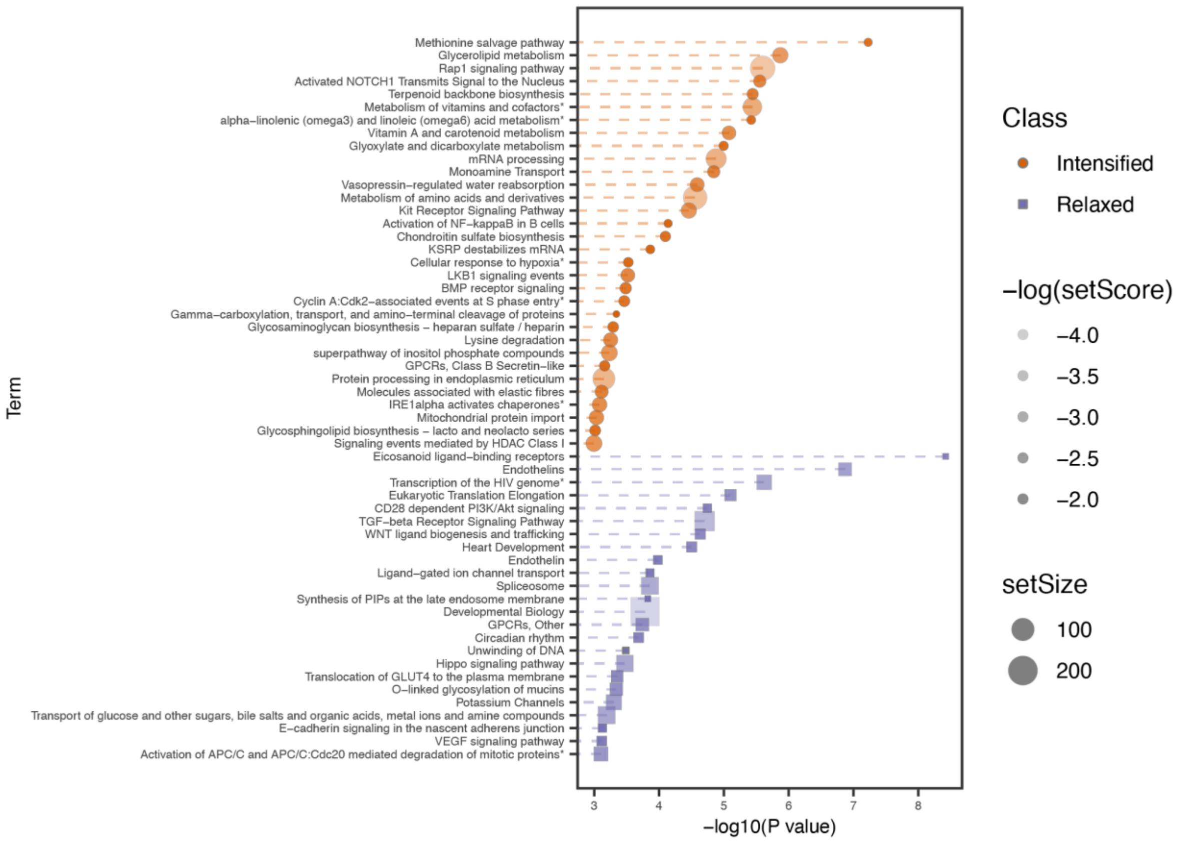

Appendix Fig S13 Gene set enrichment of genes under relaxed or strengthened selection. Polysel gene set enrichment analysis of genes under relaxed or strengthened selection. The x-axis is the *P* value on -log10 scale. Different selection uses different shapes and colors. The shade of the color represents the SUMSTAT, and the size of the dot represents the size of the gene set.

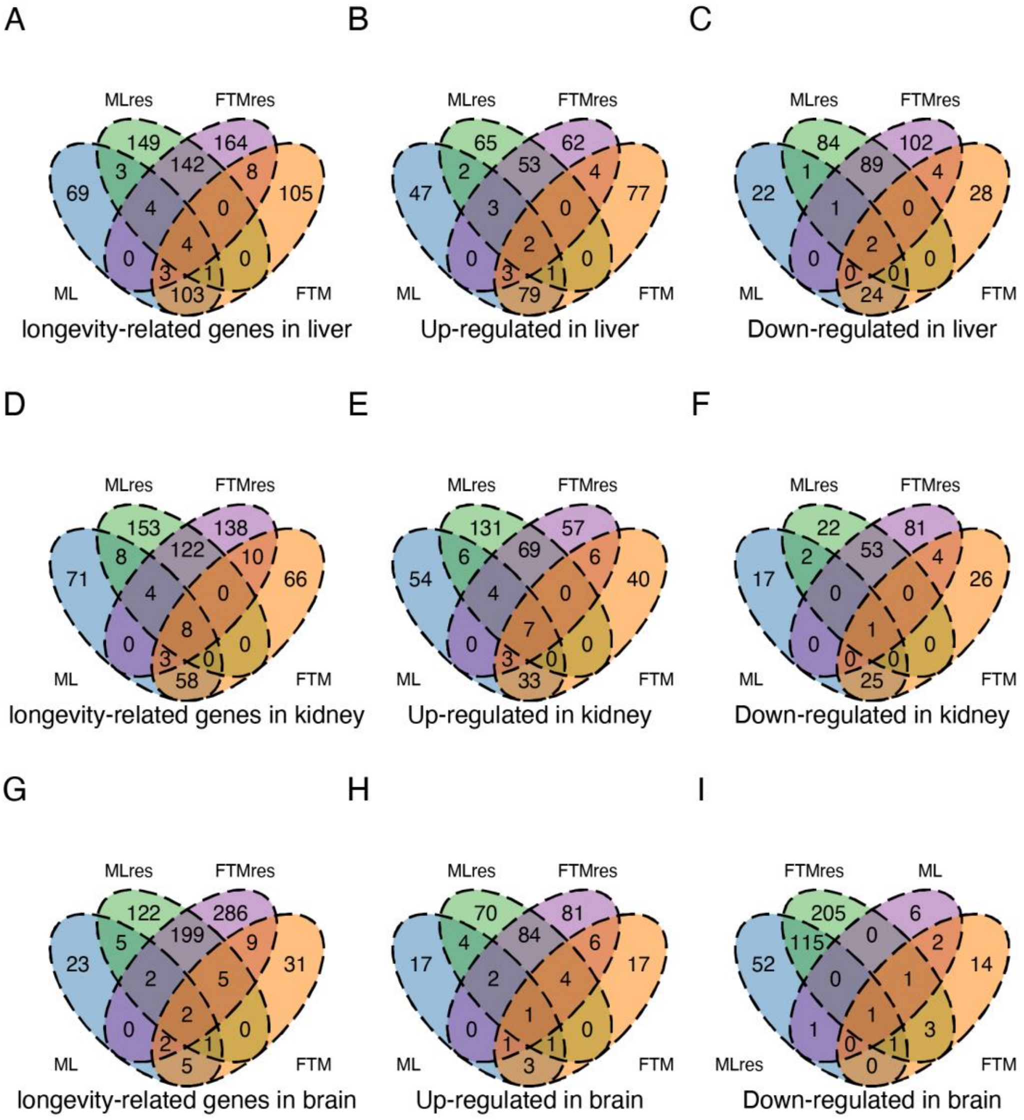

Appendix Fig S14 Overlap of longevity-related genes. (*A, D, G*) Venn diagrams of each tissue for longevity-related genes. (*B, E, H*) Venn diagrams of each tissue for positive longevity-related genes. (*C, F, I*) Venn diagrams of each tissue for negative longevity-related genes.

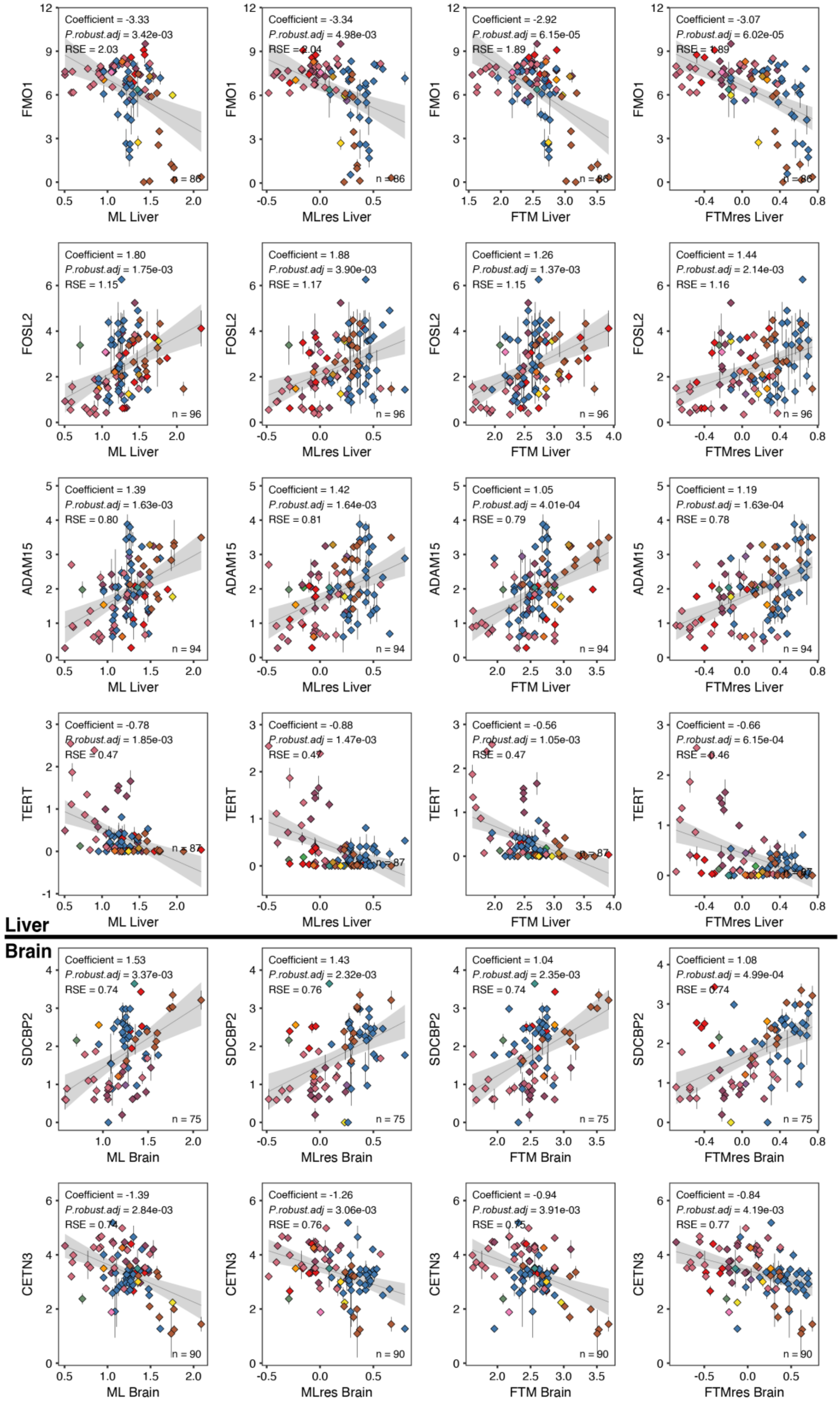
Coefficient = -3.33
P.robust.adj = 3.42e-03
RSE = 2.03
n = 86
FMO1
ML Liver
Coefficient = -3.34
P.robust.adj = 4.98e-03
RSE = 2.04
n = 86
MLres Liver
Coefficient = -2.92
P.robust.adj = 6.15e-05
RSE = 1.89
n = 86
FTM Liver
Coefficient = -3.07
P.robust.adj = 6.02e-05
RSE = 1.89
n = 86
FTMres Liver
Coefficient = 1.80
P.robust.adj = 1.75e-03
RSE = 1.15
n = 96
FOSL2
Coefficient = 1.88
P.robust.adj = 3.90e-03
RSE = 1.17
Coefficient = 1.26
P.robust.adj = 1.37e-03
RSE = 1.15
Coefficient = 1.44
P.robust.adj = 2.14e-03
RSE = 1.16
Coefficient = 1.39
P.robust.adj = 1.63e-03
RSE = 0.80
n = 94
ADAM15
Coefficient = 1.42
P.robust.adj = 1.64e-03
RSE = 0.81
Coefficient = 1.05
P.robust.adj = 4.01e-04
RSE = 0.79
Coefficient = 1.19
P.robust.adj = 1.63e-04
RSE = 0.78
Coefficient = -0.78
P.robust.adj = 1.85e-03
RSE = 0.47
n = 87
TERT
Coefficient = -0.88
P.robust.adj = 1.47e-03
RSE = 0.47
Coefficient = -0.56
P.robust.adj = 1.05e-03
RSE = 0.47
Coefficient = -0.66
P.robust.adj = 6.15e-04
RSE = 0.46
Liver
Brain
Coefficient = 1.53
P.robust.adj = 3.37e-03
RSE = 0.74
n = 75
SDCBP2
ML Brain
Coefficient = 1.43
P.robust.adj = 2.32e-03
RSE = 0.76
MLres Brain
Coefficient = 1.04
P.robust.adj = 2.35e-03
RSE = 0.74
FTM Brain
Coefficient = 1.08
P.robust.adj = 4.99e-04
RSE = 0.74
FTMres Brain
Coefficient = -1.39
P.robust.adj = 2.84e-03
RSE = 0.74
n = 90
CETN3
Coefficient = -1.26
P.robust.adj = 3.06e-03
RSE = 0.76
Coefficient = -0.94
P.robust.adj = 3.91e-03
RSE = 0.75
Coefficient = -0.84
P.robust.adj = 4.19e-03
RSE = 0.77

Appendix Fig S15 Overlap genes in liver and brain. In each figure, the y-axis is the scaled expression level of each gene with 0 as the center, and the x-axis is the longevity traits (ML: maximum lifespan; FTM: female time to maturity; MLres and FTMres: ML and FTM residuals adjusted for adult weight, respectively). Error bars represents SE. Potential outliers have been removed. The coefficient of phylogenetic regression, adj.P.robust and residual standard error (RSE) is included in the figure.

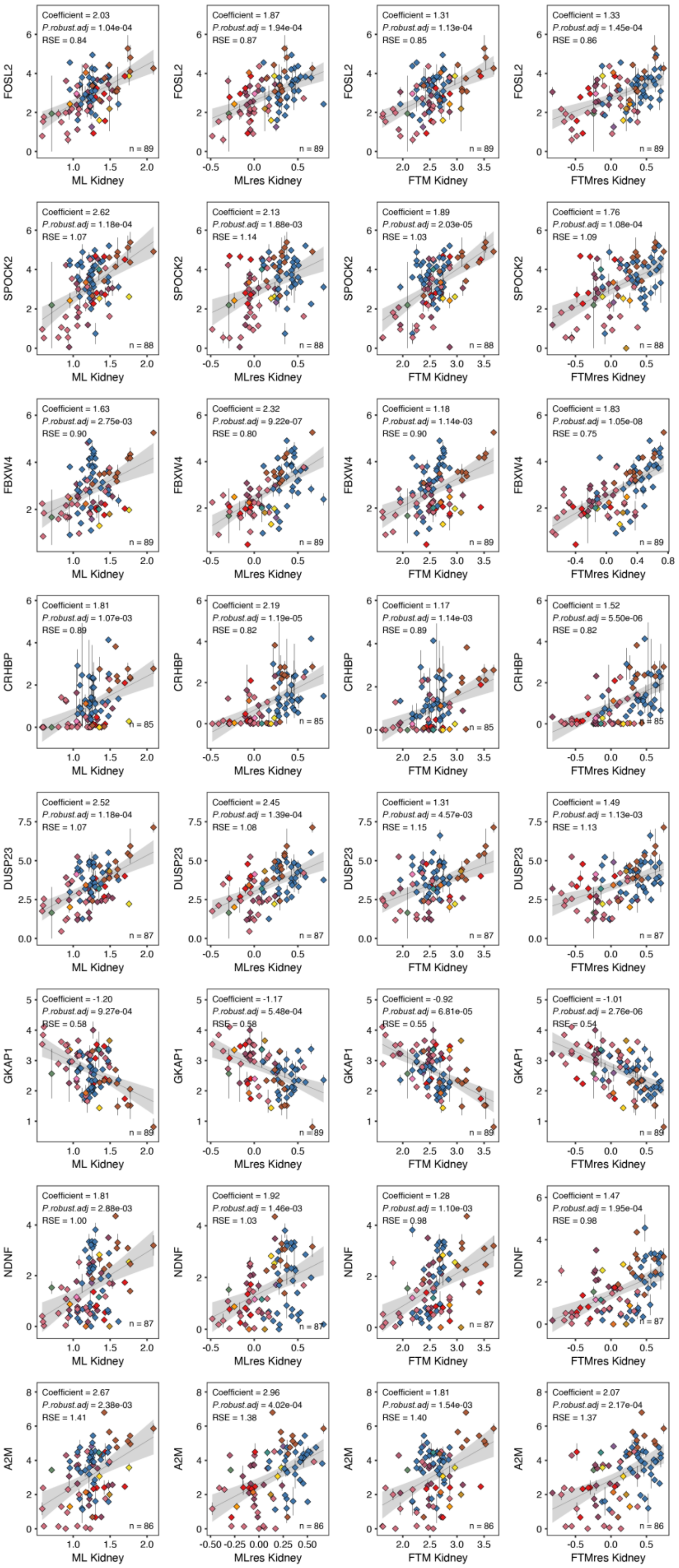
Coefficient = 2.03
P.robust.adj = 1.04e-04
RSE = 0.84
n = 89
FOSL2
ML Kidney
Coefficient = 1.87
P.robust.adj = 1.94e-04
RSE = 0.87
n = 89
FOSL2
MLres Kidney
Coefficient = 1.31
P.robust.adj = 1.13e-04
RSE = 0.85
n = 89
FOSL2
FTM Kidney
Coefficient = 1.33
P.robust.adj = 1.45e-04
RSE = 0.86
n = 89
FOSL2
FTMres Kidney
Coefficient = 2.62
P.robust.adj = 1.18e-04
RSE = 1.07
n = 88
SPOCK2
ML Kidney
Coefficient = 2.13
P.robust.adj = 1.88e-03
RSE = 1.14
n = 88
SPOCK2
MLres Kidney
Coefficient = 1.89
P.robust.adj = 2.03e-05
RSE = 1.03
n = 88
SPOCK2
FTM Kidney
Coefficient = 1.76
P.robust.adj = 1.08e-04
RSE = 1.09
n = 88
SPOCK2
FTMres Kidney
Coefficient = 1.63
P.robust.adj = 2.75e-03
RSE = 0.90
n = 89
FBXW4
ML Kidney
Coefficient = 2.32
P.robust.adj = 9.22e-07
RSE = 0.80
n = 89
FBXW4
MLres Kidney
Coefficient = 1.18
P.robust.adj = 1.14e-03
RSE = 0.90
n = 89
FBXW4
FTM Kidney
Coefficient = 1.83
P.robust.adj = 1.05e-08
RSE = 0.75
n = 89
FBXW4
FTMres Kidney
Coefficient = 1.81
P.robust.adj = 1.07e-03
RSE = 0.89
n = 85
CRHBP
ML Kidney
Coefficient = 2.19
P.robust.adj = 1.19e-05
RSE = 0.82
n = 85
CRHBP
MLres Kidney
Coefficient = 1.17
P.robust.adj = 1.14e-03
RSE = 0.89
n = 85
CRHBP
FTM Kidney
Coefficient = 1.52
P.robust.adj = 5.50e-06
RSE = 0.82
n = 85
CRHBP
FTMres Kidney
Coefficient = 2.52
P.robust.adj = 1.18e-04
RSE = 1.07
n = 87
DUSP23
ML Kidney
Coefficient = 2.45
P.robust.adj = 1.39e-04
RSE = 1.08
n = 87
DUSP23
MLres Kidney
Coefficient = 1.31
P.robust.adj = 4.57e-03
RSE = 1.15
n = 87
DUSP23
FTM Kidney
Coefficient = 1.49
P.robust.adj = 1.13e-03
RSE = 1.13
n = 87
DUSP23
FTMres Kidney
Coefficient = -1.20
P.robust.adj = 9.27e-04
RSE = 0.58
n = 89
GKAP1
ML Kidney
Coefficient = -1.17
P.robust.adj = 5.48e-04
RSE = 0.58
n = 89
GKAP1
MLres Kidney
Coefficient = -0.92
P.robust.adj = 6.81e-05
RSE = 0.55
n = 89
GKAP1
FTM Kidney
Coefficient = -1.01
P.robust.adj = 2.76e-06
RSE = 0.54
n = 89
GKAP1
FTMres Kidney
Coefficient = 1.81
P.robust.adj = 2.88e-03
RSE = 1.00
n = 87
NDNF
ML Kidney
Coefficient = 1.92
P.robust.adj = 1.46e-03
RSE = 1.03
n = 87
NDNF
MLres Kidney
Coefficient = 1.28
P.robust.adj = 1.10e-03
RSE = 0.98
n = 87
NDNF
FTM Kidney
Coefficient = 1.47
P.robust.adj = 1.95e-04
RSE = 0.98
n = 87
NDNF
FTMres Kidney
Coefficient = 2.67
P.robust.adj = 2.38e-03
RSE = 1.41
n = 86
A2M
ML Kidney
Coefficient = 2.96
P.robust.adj = 4.02e-04
RSE = 1.38
n = 86
A2M
MLres Kidney
Coefficient = 1.81
P.robust.adj = 1.54e-03
RSE = 1.40
n = 86
A2M
FTM Kidney
Coefficient = 2.07
P.robust.adj = 2.17e-04
RSE = 1.37
n = 86
A2M
FTMres Kidney

183

Appendix Fig S16 Overlap genes in kidney. In each figure, the y-axis is the scaled expression level of each gene with 0 as the center, and the x-axis is the longevity traits (ML: maximum lifespan; FTM: female time to maturity; MLres and FTMres: ML and FTM residuals adjusted for adult weight, respectively). Error bars represents SE. Potential outliers have been removed. The coefficient of phylogenetic regression, adj.P.robust and residual standard error (RSE) is included in the figure.

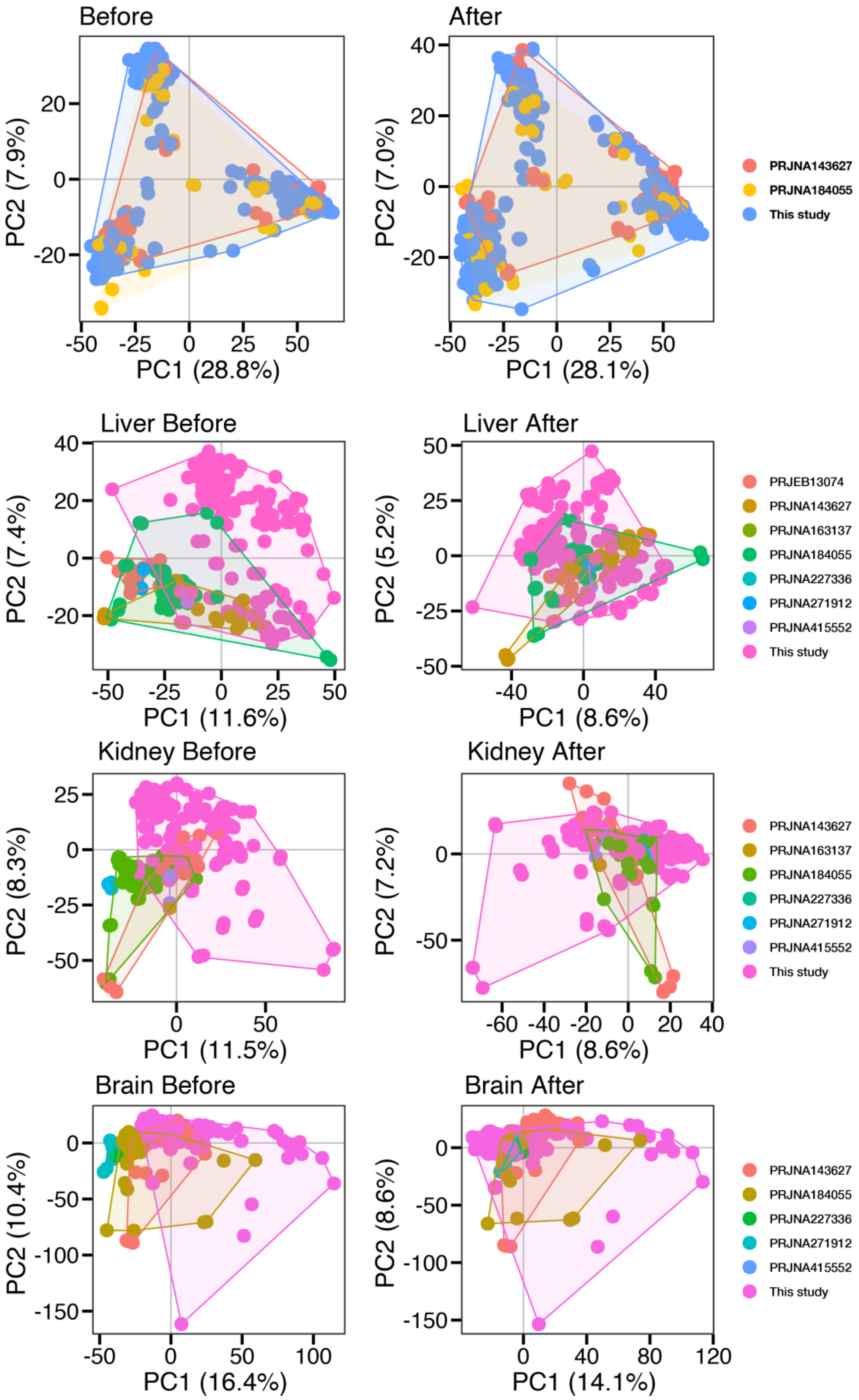

Appendix Fig S17 PCA of before and after batch correction.

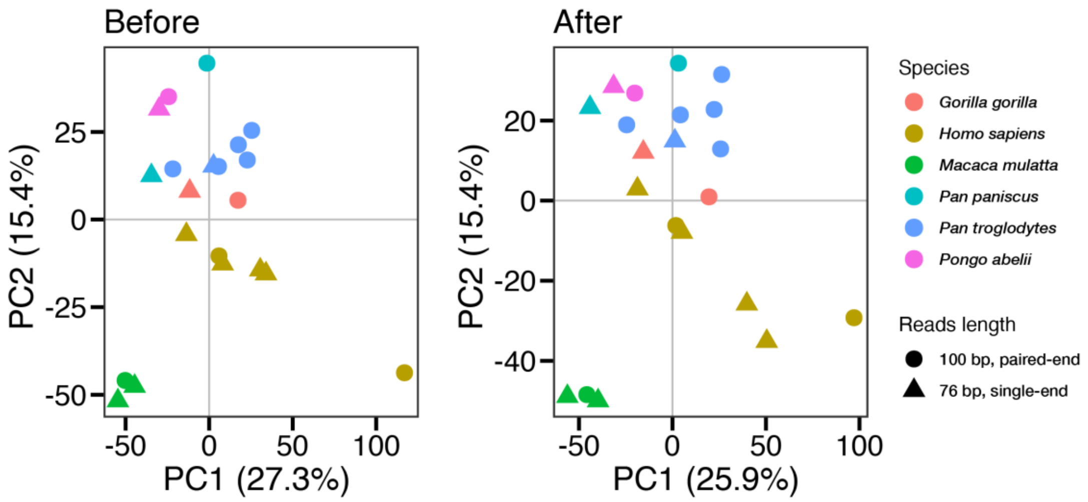

Appendix Fig S18 Comparison of the impact of reads length on PCA before and after batch correction

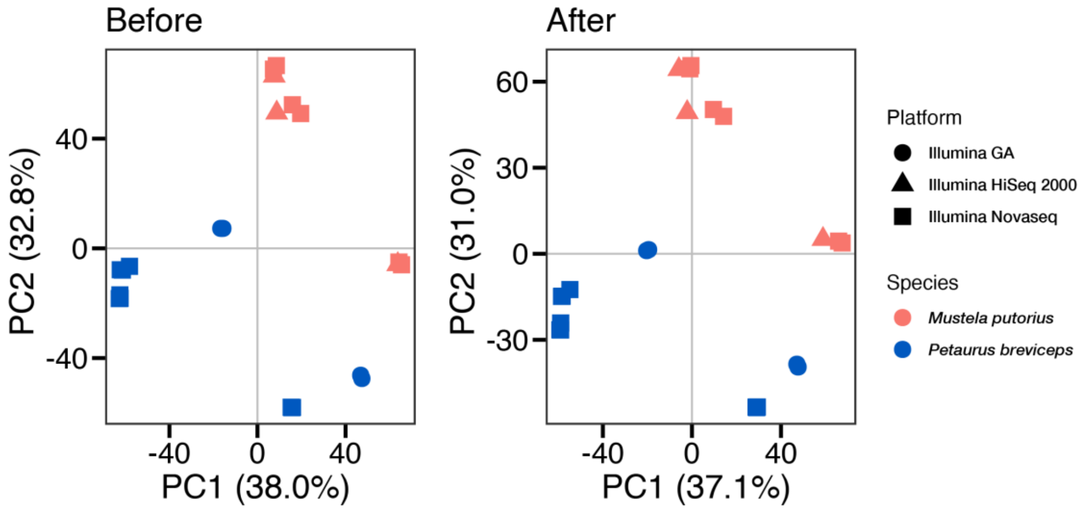

Appendix Fig S19 Comparison of the impact of sequencing platforms on PCA before and after batch correction.
