# Supplementary material for: Large‐scale across species transcriptomic analysis identifies genetic selection signatures associated with longevity in mammals
Source: EMBO J. 2023 Jul 10;42(17):e112740. doi: 10.15252/embj.2022112740 (PMC10476176; doi:10.15252/embj.2022112740)
Supplement: Supplementary file 22 [file EMBJ-42-e112740-s020.pdf]

THE EMBO JOURNAL

Large-scale across species transcriptomic analysis identifies genetic selection signatures associated with longevity in mammals

Weiqiang Liu, Pingfen Zhu, Meng Li, Zihao Li, Yang Yu, Gaoming Liu, Juan Du, Xiao Wang, Jing Yang, Ran Tian, Inge Seim, Alaattin Kaya, Mingzhou Li, Ming Li, Vadim Gladyshev, and Xuming Zhou
DOI: 10.15252/embj.2022112740

Corresponding author(s): Xuming Zhou (zhouxuming@ioz.ac.cn)

Review Timeline:

Submission Date:	4th Oct 22
Editorial Decision:	2nd Jan 23
Revision Received:	1st Apr 23
Editorial Decision:	12th May 23
Revision Received:	12th May 23
Accepted:	22nd May 23

Editor: Kelly Anderson

Transaction Report:

(Note: With the exception of the correction of typographical or spelling errors that could be a source of ambiguity, letters and reports are not edited. Depending on transfer agreements, referee reports obtained elsewhere may or may not be included in this compilation. Referee reports are anonymous unless the Referee chooses to sign their reports.)

1st Editorial Decision 2nd Jan 2023

Dear Dr. Zhou,

Thank you for submitting your manuscript for consideration by the EMBO Journal. It has now been seen by three referees whose comments are shown below.

Should you be able to address these criticisms in full, we could consider a revised manuscript. It would be good to discuss your plan to address referee concerns and I am available to do so via email or zoom in the coming weeks. I should remind you that it is EMBO Journal policy to allow a single round of revision only and that, therefore, acceptance or rejection of the manuscript will depend on the completeness of your responses in this revised version. Please bear in mind that decision letters, referee comments and your point by point response will form part of the Review Process File, and will therefore be available online to the community. For more details on our Transparent Editorial Process, please visit our website: https://www.embo.org/embo-press

We generally allow three months as standard revision time. As a matter of policy, competing manuscripts published during this period will not negatively impact on our assessment of the conceptual advance presented by your study. However, we request that you contact the editor as soon as possible upon publication of any related work, to discuss how to proceed. Should you foresee a problem in meeting this three-month deadline, please let us know in advance and we may be able to grant an extension. I have attached a guide for revisions to this email for your convenience.

Thank you for the opportunity to consider your work for publication. I look forward to your revision.

Yours sincerely,

Kelly M Anderson, PhD
Editor
The EMBO Journal
k.anderson@embojournal.org

Further information is available in our Guide For Authors: https://www.embopress.org/page/journal/14602075/authorguide

The revision must be submitted online within 90 days; please click on the link below to submit the revision online before 2nd Apr 2023.

https://emboj.msubmit.net/cgi-bin/main.plex

Referee #1:

This is very important study, in which the authors compared transcriptome differences in three organs (liver, brain and kidney) between 106 different mammalian species. The authors demonstrate that IGF and FoxO signaling pathways show associations with mammalian longevity. Also, methionine restriction related genes appear to be under strong selection in long-lived mammals. The authors conclude that mammalian longevity is regulated through polygenic model and indirect selection.
Overall, manuscript is well-written and illustrated.

The only comment is that the authors need to provide a correlation between the longevity-associated genes and human disease-associated genes for the organs analyzed. Since the common pathology for all three organs is cancer, correlation of the longevity associated and cancer-related genes (https://cancer.sanger.ac.uk/census) provided as a final part of the manuscript would strengthen this very important work.

Referee #2:

This is a very interesting manuscript that build ups on the recent analysis of multi species data on longevity to identify clues to pathways involved in life extension history. This is a manuscript that is very difficult to judge and analyze there are several data sets and the analyzes is certainly very complex and requires a substantial expertise in bioinformatics. From the biological point of view this is certainly a provocative manuscript that could serve as a source of data that may drive further investigation on the field.
Therefore, I find it very difficult to evaluate. It would certainly be a manuscript that would be of use to many investigators and

may have high citations.
The main potential issues are related with the comparison of the historical data with new sets that may not be all done the same way. This needs to be certificated on should be discussed as a major limitation of the study.
Another main issue is that it is not clear at which time in the life span cycle has the data been generated and it is possible that the transcriptomics data may change significantly within the same species during the lifespan of each organism, this certainly needs to be taken in consideration and has to be also discussed and perhaps analyzed experimentally.
Overall, this is a very complex big data manuscript that provides a very unique data set that is provocative and can be of significant value to the aging community but needs to be interpretation with caution.

Referee #3:

In this study, Liu and colleagues analyse transcriptomics data from 3 tissues, spanning 106 mammals to find genes that correlate and may co-evolve with lifespan. They generate a new dataset comprising 56 species and combine this dataset with the publicly available transcriptomics data from 50 species. They use tau index for the analysis of tissue and species specificity, phylogenetic models to analyse gene expression and sequence evolution, and they analyse their findings in comparison with longevity associated traits (i.e. maximum lifespan, female time to maturity, and residuals after correcting for the adult body weight). I believe results from this line of work are important and interesting for both evolutionary biologists and ageing researchers.

While I appreciate the effort to create a comprehensive dataset through data integration, I have concerns about this process as detailed below. Below I outline my major comments:

1) Data integration: The authors attempted to integrate data generated through different technologies in different labs in order to create a comprehensive dataset. While the authors stated that they used ComBat for batch correction, they did not provide any detailed description or exploratory data analysis demonstrating that this correction was effective. This is a concern as the effectiveness of batch correction is crucial to the validity of the study.

Table S1 lists the datasets used in the study, including cases where one dataset only provides data for a single species, with no other dataset involving that species (e.g. Balaena mysticetus). In these cases, it is not possible to correct for batch effects using ComBat as the biological factor (species) completely overlaps with the batch. There are additional cases like Castor canadensis, Heterocephalus glaber, and Cryptomys damarensis... where this is an issue. The authors should remove these cases from the dataset and provide EDA demonstrating the effectiveness of their batch correction. For example, the authors could calculate pairwise transcriptomic (parametric) and phylogenetic distances between species and check if the distribution is as expected. Outliers could suggest poor integration and should be excluded.

Supplementary Figure 1C shows a correlation plot between samples, with an overall positive correlation between kidney and liver samples and a negative correlation between brain and the other tissues. 60% of all kidney and liver samples were generated in the present study, while all brain data were obtained from public repositories. The high correlation between kidney and liver raises further concerns about the effectiveness of batch correction. Indeed, the authors report brain had the highest number of brain specific genes (6.7k compared to 2.5k and 1.2k in others) - however, this might be driven (or amplified) by the fact that brain data is collected from other experiments and the batch correction is not effective.

Some of the datasets used in the study also include tissue samples from the same animals, which should be accounted for in the models.

2) Lack of age information for animals used in the study: While it is understandable that it can be difficult to compile comparable data across different studies, it is important to consider that gene expression changes with age. It is possible that the authors did not study comparable time points across species and that their results (e.g. finding mRNA surveillance negatively correlated with lifespan) are confounded by age-related changes in gene expression due to study bias in long-lived species.

For example, the naked mole rat samples in the study include not only young adult samples, as in most of the other datasets, but also samples from 20-year-old NMRs. This means that their average expression value would incorporate age-related changes in expression for this species. The same bias may be present in other long-lived animals. The authors should provide additional information showing that their data is comparable and exclude other samples (the dataset also includes a limited number of juvenile samples), or analyse the data separately.

It is important to note that a significant proportion of the data was generated either in the present study or in the Fushan et al. paper, which shares some authors with this study. Therefore, age information should be available for at least these samples.

3) Tissue and species specificity analysis: In addition to the potential impact of batch correction, it is possible that gene expression levels are confounding this analysis as the authors do not seem to scale the gene expression levels. Highly expressed genes can result in higher variation and higher tau values. If the calculation includes a correction for this, the authors should mention it in the methods.

I am also surprised that there is not even one gene that is stably expressed across mammals, including housekeeping genes. It is possible that the tau index does not provide enough resolution for this analysis, or that the threshold should be changed considering the breadth of the sampling.

When the authors say they used the sum of the tau index for pathway analysis, do they mean the average? Otherwise, this value would be dramatically affected by the number of genes in a pathway.

4) Longevity-related genes: The authors found that only 76 out of 974 longevity-regulating genes were associated with lifespan across phylogeny. Is this number statistically significant?

5) Expression-trait correlation plots: I am confused by Fig 2E-G, supplementary figures S8, and S12, which show correlations between some of the genes the authors emphasize and longevity traits. Although the p values are quite significant, these plots show almost no correlation. Is this due to the effect of phylogeny? If so, perhaps showing correlations within taxa would be more informative. Otherwise, despite the significant p values, it does not appear that any of these traits explain a significant portion of the variation in the data (perhaps r2 values from PGLS analysis would be helpful in this regard).

6) Selection signatures: The authors used the absolute number of years (ML >30), rather than a metric relative to the taxa. I am concerned that this may lead to the study of taxa with longer lifespans rather than species that have evolved longer lifespans. Can the authors provide more information on this choice and its potential impact on the results? I think it might be more helpful to perform a taxa-specific analysis to better understand selection signatures.

Detailed responses to reviewers

All comments provided by reviewers are in gray italics, and our responses are in blue. All revisions in the manuscript are marked in red.

REVIEWERS COMMENTS

Reviewer #1

Comment 1-1:

This is very important study, in which the authors compared transcriptome differences in three organs (liver, brain and kidney) between 106 different mammalian species. The authors demonstrate that IGF and FoxO signaling pathways show associations with mammalian longevity. Also, methionine restriction related genes appear to be under strong selection in long-lived mammals. The authors conclude that mammalian longevity is regulated through polygenic model and indirect selection.
Overall, manuscript is well-written and illustrated.

Response 1-1:

We sincerely appreciate these positive remarks.

Comment 1-2:

The only comment is that the authors need to provide a correlation between the longevity-associated genes and human disease-associated genes for the organs analyzed. Since the common pathology for all three organs is cancer, correlation of the longevity associated and cancer-related genes (https://cancer.sanger.ac.uk/census) provided as a final part of the manuscript would strengthen this very important work.

Response 1-2:

Thank you for your suggestion. This is really an important idea.

During revision, we performed a Fisher's exact test of longevity-related and cancer-related genes, as suggested, and found that longevity-related genes were enriched for carcinogenic genes in the three tissues examined. We have added the result to the section "Comparison of longevity-correlated genes among tissues and gene sets": (**Lines 397-403, Fig 3D and Table R1**) [In addition, we found that longevity-related genes in the three tissues were enriched for carcinogenic genes (Liver-ML-Both P = 0.04, odds ratio = 2.69; Kidney-MLres-Both P = 0.03, odds ratio = 2.13; Brain-FTMres-Both P = 0.02, odds ratio = 1.83) and disease-harboring genes were enriched only in the liver (P = 0.04, odds ratio = 1.36), hinting that carcinogenesis is a restrictive factor for organ aging and function.]. Combined with Fisher's exact test for other categories (**Fig 3D** and **Dataset EV8**), we have updated the results as

[Longevity-related genes enriched for essential genes (Liver-ML-Both P = 0.01, odds ratio = 1.58; Kidney-FTMres-Both P = 0.02×10^{-2}, odds ratio = 1.80; Brain-MLres-Both P =0.02×10^{-2}, odds ratio = 1.72) and old genes (i.e., genes that originated before the emergence of mammals) (Liver-FTM-Both P = 0.03×10^{-2}, odds ratio = 1.64; Kidney-MLres-Both P = 0.02, odds ratio = 1.31; Brain-FTMres-Both P = 0.01×10^{-1}, odds ratio = 1.37) in all tissues (**Fig 3D Dataset EV8** and **Methods**). Most essential genes were also old genes, indicating that expression changes of genes with essential functions in cells are important for lifespan control across species.] (**Lines 389-397, Fig 3D**).

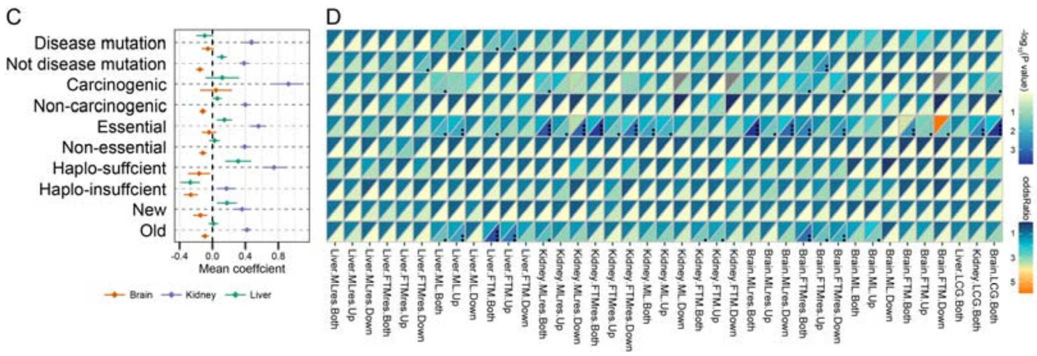

Figure 3C and D: The correlation among different gene categories and longevity-related genes.

(C) On the x-axis, the coefficient represents the rate of gene expression variation with life-history gradient. Data are presented as means with error bars.

(D) Heatmap of Fisher's exact test. The upper triangle heatmap represents the odds ratio and the lower the $-\log_{10}(P$ value). The black point represents significance levels (P<0.05: ·; P<0.01: ··; P<0.001: ···). LCG represents the longevity-correlated gene.

Table R1. Fisher exact test of carcinogenic and disease-harboring genes.

Tissue	Trait	Category	Pvalue	oddsRatio	Direction	Alternative
**Liver**	ML	Carcinogenic	0.0442	2.6932	Both	Greater
**Kidney**	MLres	Carcinogenic	0.0329	2.1348	Both	Greater
**Brain**	FTMres	Carcinogenic	0.0188	1.8304	Both	Greater
**Brain**	FTMres	Carcinogenic	0.0334	2.0201	Down	Greater
**Brain**	Core	Carcinogenic	0.0391	2.4289	Both	Greater
**Liver**	FTM	Disease mutation	0.0176	1.5304	Up	Greater
**Liver**	ML	Disease mutation	0.0221	1.5734	Up	Greater
**Liver**	FTM	Disease mutation	0.0355	1.3599	Both	Greater

We have added the relevant information to the Methods as well (**Lines 807-808**) [Cancer-related genes were obtained from The Cancer Gene Census (CGC) (https://cancer.sanger.ac.uk/census) (Tate *et al*, 2018)], and (**Lines 823-826**) [We conducted gene enrichment analysis of positively

correlated genes (Up), negatively correlated genes (Down) and all genes (Both) using Fisher's exact test].

Reviewer #2

Comment 2-1:

This is a very interesting manuscript that build ups on the recent analysis of multi species data on longevity to identify clues to pathways involved in life extension history. This is a manuscript that is very difficult to judge and analyze there are several data sets and the analyzes is certainly very complex and requires a substantial expertise in bioinformatics. From the biological point of view this is certainly a provocative manuscript that could serve as a source of data that may drive further investigation on the field.

Therefore, I find it very difficult to evaluate. It would certainly be a manuscript that would be of use to many investigators and may have high citations.

Response 2-1:

Thank you very much for these positive remarks and valuable comments.

Comment 2-2:

The main potential issues are related with the comparison of the historical data with new sets that may not be all done the same way. This needs to be certificated on should be discussed as a major limitation of the study.

Response 2-2:

Thank you for this comment. We appreciate your concern about the data integration issues. We agree that potential influencing factors (e.g., read type and sequencing platforms) should be considered, and we have taken steps to address them. As mentioned in the Methods section, we have considered batch effects from different sources (i.e., NCBI BioProject) and sequencing platforms. We have re-organized the Methods section to make this clearer (**Lines 590-594**) [The comBat_seq in the R package sva (Leek *et al*, 2012) was applied to correct for batch effect, including two factors that likely affect the data: data source (i.e., NCBI BioProject and sequencing batches of our data) and sequencing platform (Fukushima & Pollock, 2020). Tissue and species were set as fixed effects].

During revision, we conducted additional analyses to assess batch effects: First, the data are mainly from three resources: new sequencing data of this study and data generated by Fushan *et al* (2015) and Brawand *et al* (2011). To verify the effectiveness of batch correction, we performed PCA on these datasets and conducted a Kruskal-Wallis rank sum test on PC1 before and after batch correction (**Fig R1**). The results show that, before batch correction, PC1 separates tissues and also significantly distinguishes samples by

BioProject (Kruskal-Wallis chi-squared = 30.19, *P* = 0.02), indicating that the study source has effects. After batch correction, PC1 consistently separated the tissues but not sample source (Kruskal-Wallis chi-squared = 15.30, *P* > 0.50). In addition, we further repeat analyses by tissue (**Fig R1**) and consistently found that the main components do not distinguish the samples by BioProject (Liver: *P* = 0.71; Kidney: *P* = 0.04; Brain: *P* = 0.05) after batch effect correction. These results indicate that BioProject-associated batch effects were efficiently removed.

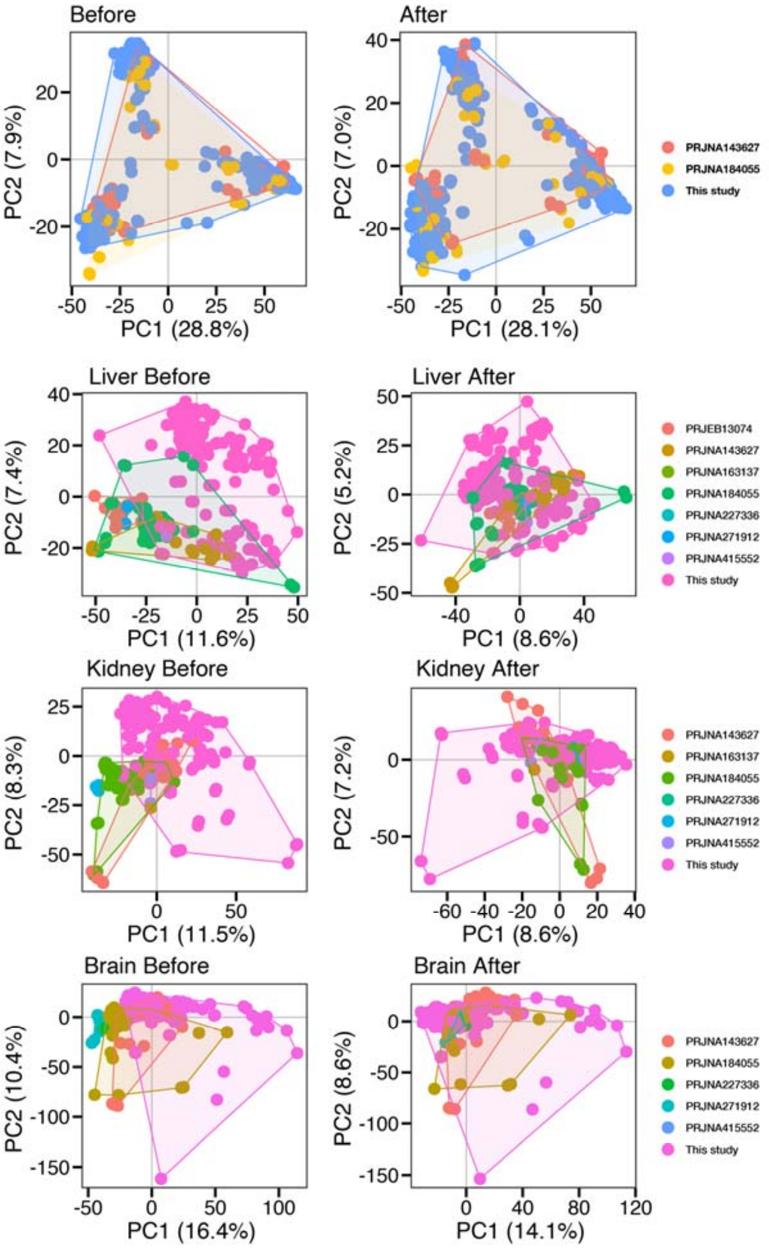

Figure R1: PCA analyses of data before and after batch correction.

We also tested the influence of the read type using the data set generated by Brawand *et al* (2011) (NCBI BioProject PRJNA143627). Their data includes brain RNA-seq (76 bp single-end and 100bp paired-end) generated on the Illumina Genome Analyzer IIx platform. Comparing different read types from the same BioProject, PC1 and PC2 were separated by species but not read type before and after batch removal (**Fig R2**), indicating a limited effect.

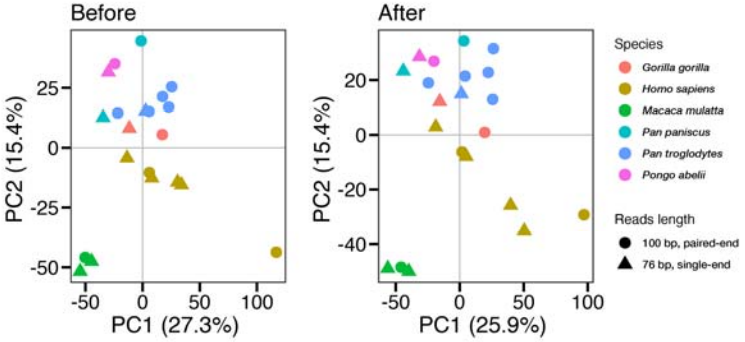

Figure R2: The impact of reads length on PCA before and after batch correction.

Finally, we used the data from the ferret (*Mustela putorius*) and sugar glider (*Petaurus breviceps*) to examine the batch effect of sequencing platforms. The ferret data was generated on the Illumina HiSeq 2000 platform, and the sugar glider data using the Illumina Genome Analyzer IIx. Our data from the same species were generated on the Illumina Novaseq 6000 instrument. PC1 and PC2 separated the samples by species but not by sequencing platforms (**Fig R3**), indicating that the sequencing platform does not significantly affect gene expression.

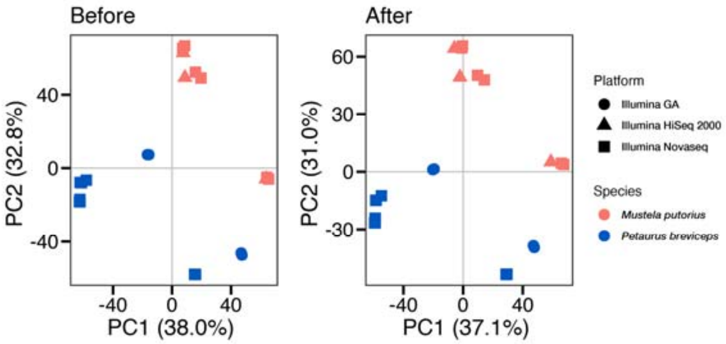

Figure R3: The impact of sequencing platforms on PCA before and after batch correction.

Our results show that the reads type and sequencing platform across different studies does not significantly affect a species' gene expression

variation. Integrating data from BioProjects impacts gene expression estimates; however, the batch correction removed this variability from our data. Other factors that may influence the results, but cannot be easily removed, have been mentioned in the Discussion as (**Lines 483-487**) [In addition, although we have attempted to control for batch effects (e.g., NCBI BioProject and sequencing platform), additional sources of unwanted variation exist and were not considered in our study (e.g., sample status, library preparation, genome quality, and ortholog calling).]

We also added related information and figure in Methods and Supplementary file. Fig R1, Fig R2 and Fig R3 were added to the Supplementary file as Appendix Fig S17, Appendix Fig S18 and Appendix Fig S19.

In Methods section (**Lines 597-603**), we added [

To verify the effectiveness of batch correction, we performed PCA on these three datasets and conducted a Kruskal-Wallis rank sum test (K-W test) on PC1 before and after batch correction (**Table EV5**). There was no significant difference between different BioProjects after correction (**Appendix Results**). Moreover, the read type (short- vs. paired-end reads) and sequencing platform had little effect on gene expression variation after batch correction (**Appendix Results**).]

Detail approaches and results are available at Supplementary file (**Lines 19-47**) [

Batch effect assessment

To verify the effectiveness of batch correction, we selected several subsets to compare the batch correction of BioProject, reads type (short- vs. paired-end reads), and sequencing platform. The data were mainly from three sources: new sequencing data generated in this study and data generated by Fushan *et al.* (2015) and Brawand *et al.* (2011). We performed PCA and conducted a Kruskal-Wallis rank sum (K-W) test on PC1 before and after batch correction. The results showed that before correction PC1 separates the samples by tissues but also by BioProject (Kruskal-Wallis chi-squared = 30.19, P = 0.02). After batch correction, PC1 separated the samples by tissue but not by BioProject (Kruskal-Wallis chi-squared = 15.30, P = 0.50). In addition, we examined each tissue separately and consistently found that the main components did not distinguish the samples by BioProject after removing batch effect (Liver: P = 0.71; Kidney: P = 0.04; Brain: P = 0.05) (**Appendix Fig S17** and **Table EV5**).

We then tested the influence of the read type using the data set generated by Brawand *et al*. (2011) (NCBI BioProject: PRJNA143627). This data set

includes brain RNA-seq reads of different types (76bp single-end and 100bp paired-end) generated on the Illumina Genome Analyzer IIx platform. PC1 and PC2 separated samples by species but not read type before and after batch removal (**Appendix Fig S18**), indicating the read type has a limited influence on gene expression variation.

Finally, we used the data from the ferret (*Mustela putorius*) and sugar glider (*Petaurus breviceps*) to examine the batch effect of sequencing platforms. The ferret data was generated on the Illumina HiSeq 2000 platform, and the sugar glider data using the Illumina Genome Analyzer IIx. Our data from the same species were generated on the Illumina Novaseq 6000 instrument. PC1 and PC2 separated samples by species but not by sequencing platforms (**Appendix Fig S19**), indicating that the sequencing platform does not significantly affect gene expression.].

Comment 2-3:

Another main issue is that it is not clear at which time in the life span cycle has the data been generated and it is possible that the transcriptomics data may change significantly within the same species during the lifespan of each organism, this certainly needs to be taken in consideration and has to be also discussed and perhaps analyzed experimentally.

Overall, this is a very complex big data manuscript that provides a very unique data set that is provocative and can be of significant value to the aging community but needs to be interpretation with caution.

Response 2-3:

Thank you for raising this issue. We agree that the chronological age of samples would affect gene expression. Unfortunately, obtaining information on the exact age of wild specimens is challenging. It usually requires long-term field studies based on individual tagging and tracking of newborns. Nevertheless, morphological features can distinguish individuals from infants, juveniles, and adults. For example, a bat can be assigned as an adult by the degree of bone development. Thus, we adapt the strategies used in most previous studies that provided an age-categories (i.e., adults) for a sample [e.g., Ma *et al* (2015), Fushan *et al* (2015), Brawand *et al* (2011)].

However, we understand your concern and have conducted additional analyses to gauge the effect of a sample's chronological age on results. To this end, we considered a recent comparative transcriptomics of multiple organs across seven species (six mammals) at different life stages: embryos, infants, juveniles, and adults (Cardoso-Moreira *et al*, 2019). In their study, PCA analyses showed that PC1 separated the samples by tissue, PC2 separated samples by developmental stages, and both PC3 and PC4 mainly determined samples by species. Because only mature individuals were used in our study,

we selected adolescents to mature/old samples in Cardoso-Moreira *et al.*'s data set and carried out PCA analysis using our pipeline. Consistent with the pattern in our 106-species data set, PC1 separated samples by tissue and PC2 separated samples by species (**Fig R4**). Moreover, the top five principal components do not separate samples by life stage (**Fig R4**). Therefore, gene expressions of the liver, brain, and kidney are generally conserved. And, gene expression variations among mature stages are generally smaller than among species.

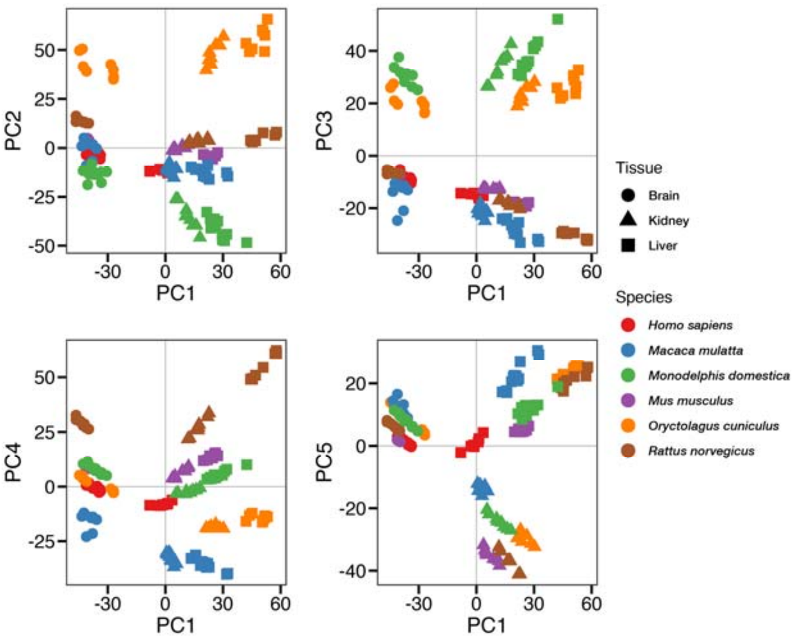

Figure R4: PCA on different life stages of mammals. Samples from adolescent to mature/old individuals were examined.

We further analyzed the Cardoso-Moreira *et al* (2019)'s data from humans and macaques whose trancriptome data from five life stages (**Fig R5**). The results show that, for the same organ (e.g., liver and brain), the gene expression variation between species is much greater than between life stages (**Fig R5**). To identify genes with expression changes at different life stages after maturity, we performed a linear regression between gene expression and developmental stages in the two primate species (genes with *P*-value < 0.01 and R-square > 0.9 were defined as age-related genes). There was no significant overlap (20 out of 515) between longevity-correlated genes identified in the current study and age-related genes. The definition of longevity-correlated genes has been added to the Results (**Lines 223-225**) [Significant genes for more than two longevity-related traits were defined as longevity-correlated genes (*n* = 669, **Table 1**, **Methods**)], and Methods (**Lines**

736-737) [we denote genes associated with two or more longevity traits as longevity-correlated genes.] (please see **Comment 3-6** for details).

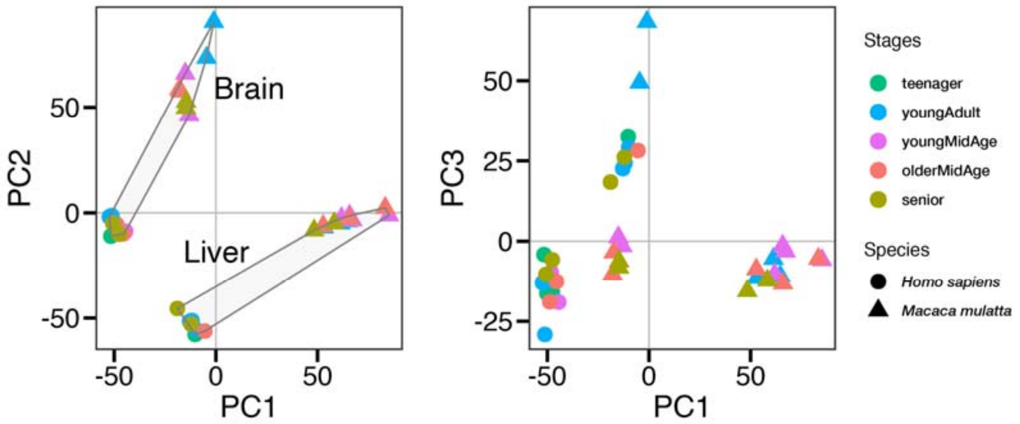

Fig R5 PCA in different life stages of *Homo sapiens* and *Macaca mulatta*.

In our 106-species data set, three species (four individuals) are with mature/older individuals (*Heterocephalus glaber*, 20 years PRJNA143625; *Pongo abelii*, 56 years, PRJNA143627; *Gorilla gorilla*, ~50 years, PRJNA143627). During revision, we excluded these individuals and repeated our analysis (we also excluded outlier samples. For details, see **Response 3-2**). The correlation of the model coefficients between the raw and new analyses was very high in all groups (0.91-0.95, Spearman's *r*), suggesting the results are highly consistent.

In summary, by analyzing the effect of age/life stages, we show that our results are generally not confounded by age-related changes in gene expression. To make the results robust, we used the results after the removal of older individuals and outlier samples. We also revised our discussion about this limitation as (**Lines 487-489**) [Finally, while it is difficult to estimate the actual age of wild samples accurately, these traits and factors should be explored in future studies.]

Reviewer #3

Comment 3-1:

In this study, Liu and colleagues analyse transcriptomics data from 3 tissues, spanning 106 mammals to find genes that correlate and may co-evolve with lifespan. They generate a new dataset comprising 56 species and combine this dataset with the publicly available transcriptomics data from 50 species. They use tau index for the analysis of tissue and species specificity, phylogenetic models to analyse gene expression and sequence evolution, and they analyse their findings in comparison with longevity associated traits (i.e., maximum lifespan, female time to maturity, and residuals after correcting for the adult body weight). I believe results from this line of work are important and interesting for both evolutionary biologists and ageing researchers.

While I appreciate the effort to create a comprehensive dataset through data integration, I have concerns about this process as detailed below. Below I outline my major comments:

Response 3-1:

We sincerely appreciated these positive remarks and valuable comments.

Comment 3-2:

1) Data integration: The authors attempted to integrate data generated through different technologies in different labs in order to create a comprehensive dataset. While the authors stated that they used ComBat for batch correction, they did not provide any detailed description or exploratory data analysis demonstrating that this correction was effective. This is a concern as the effectiveness of batch correction is crucial to the validity of the study.

Table EV1 lists the datasets used in the study, including cases where one dataset only provides data for a single species, with no other dataset involving that species (e.g., Balaena mysticetus). In these cases, it is not possible to correct for batch effects using ComBat as the biological factor (species) completely overlaps with the batch. There are additional cases like Castor canadensis, Heterocephalus glaber, and Cryptomys damarensis... where this is an issue. The authors should remove these cases from the dataset and provide EDA demonstrating the effectiveness of their batch correction. For example, the authors could calculate pairwise transcriptomic (parametric) and phylogenetic distances between species and check if the distribution is as expected. Outliers could suggest poor integration and should be excluded.

Supplementary Figure S1C shows a correlation plot between samples, with an overall positive correlation between kidney and liver samples and a negative

correlation between brain and the other tissues. 60% of all kidney and liver samples were generated in the present study, while all brain data were obtained from public repositories. The high correlation between kidney and liver raises further concerns about the effectiveness of batch correction. Indeed, the authors report brain had the highest number of brain specific genes (6.7k compared to 2.5k and 1.2k in others) - however, this might be driven (or amplified) by the fact that brain data is collected from other experiments and the batch correction is not effective.

Some of the datasets used in the study also include tissue samples from the same animals, which should be accounted for in the models.

Response 3-2:

Thank you for your thoughtful comment and suggestion. We understand your concern. We have now tested the effectiveness of batch correction on BioProject, platform, and read type (i.e., short- and paired-end) on gene expression. We found that BioProject had an impact on data and that batch corrections were able to correct for this variable. The reads type and sequencing platform across different studies did not significantly affect the variation between species. Please see a response to **Comment 2-2** for further details.

Regarding the second question, there are 13 species (from 14 BioProjects) with data from a single individual (**Table R2**), some of which are long-lived (e.g., the naked mole rat). We agree that it may be necessary to assess effects of such samples. We examined if these samples show unexpected phylogenetic positions by clustering the samples according to the gene expression of each species in each tissue (i.e., tissue-specific transcription) and calculating the distance between species (1-Pearson’s correlation coefficient). We found several samples with unexpected positions (**Table R3**). For example, a sample from the North American beaver (*Castor canadensis*) clustered with primates but not rodents (**Fig R6A-C**).

All other samples generally had reasonable phylogenetic positions (**Fig R7A-C**). For example, the Damaraland mole rat (*Cryptomys damarensis*) and naked mole rat (*Heterocephalus glaber*) clustered together, and the crab-eating macaque *Macaca fascicularis* clustered with non-human primates. During revision, we removed samples with unexpected positioning and repeated all analyses in our manuscript. We have added relative information in Methods (**Lines 595-597**) [We calculated the paired distance of samples (1-Pearson’s correlation coefficient) and excluded outlier samples and old individuals (*n* = 24) (**Dataset EV1** and **Appendix Fig S2A-C**).]

Table R2: List of 14 NCBI BioProjects with data from a single species.

BioProject id	Scientific name	Common name	ML/yrs	Tissue*
**PRJNA263931**	*Balaena mysticetus*	bowhead whale	211.0	all
**PRJNA72723**	*Balaenoptera acutorostrata*	minke whale	50.0	all
**GSE42297**	*Myotis brandtii*	Brandt’s bat	41.0	all
**PRJNA141257**	*Macaca fascicularis*	macaque	39.0	all
**PRJNA143625**	*Heterocephalus glaber*	naked mole rat	31.0	all
**PRJNA362606**	*Bos grunniens* (*Bos mutus*)	yak	26.3	kidney
**PRJNA149279**	*Bos grunniens* (*Bos mutus*)	yak	26.3	brain
**PRJEB32966**	*Castor canadensis*	North American beaver	23.4	all
**PRJNA470431**	*Callithrix jacchus*	white-tufted-ear marmoset	22.8	kidney, brain
**PRJNA580502**	*Dasypus novemcinctus*	nine-banded armadillo	22.3	brain
**PRJNA349047**	*Otolemur garnettii*	small-eared galago	20.0	all
**PRJNA218853**	*Cryptomys damarensis*	Damaraland mole rat	15.5	all
**PRJNA170104**	*Tupaia belangeri*	northern three shrew	11.1	all
**PRJNA323834**	*Acomys cahirinus*	spiny mouse	5.9	brain

* Tissue refers to the types of organs (liver, kidney, brain, and all) included in the BioProject.

Table R3: List of 14 NCBI BioProjects with data from a single species.

Scientific name	Tissue	Replicate	BioProject
Castor canadensis	Liver	1	PRJEB32966
Otolemur garnettii	Liver	1	PRJNA349047
Otolemur garnettii	Liver	2	PRJNA349047
Otolemur garnettii	Liver	3	PRJNA349047
Otolemur garnettii	Liver	4	PRJNA349047
Castor canadensis	Kidney	1	PRJEB32966
Balaena mysticetus	Kidney	1	PRJNA263931
Balaena mysticetus	Kidney	2	PRJNA263931
Balaena mysticetus	Kidney	3	PRJNA263931
Balaena mysticetus	Kidney	4	PRJNA263931
Castor canadensis	Brain	1	PRJEB32966
Balaenoptera acutorostrata	Brain	1	PRJNA72723

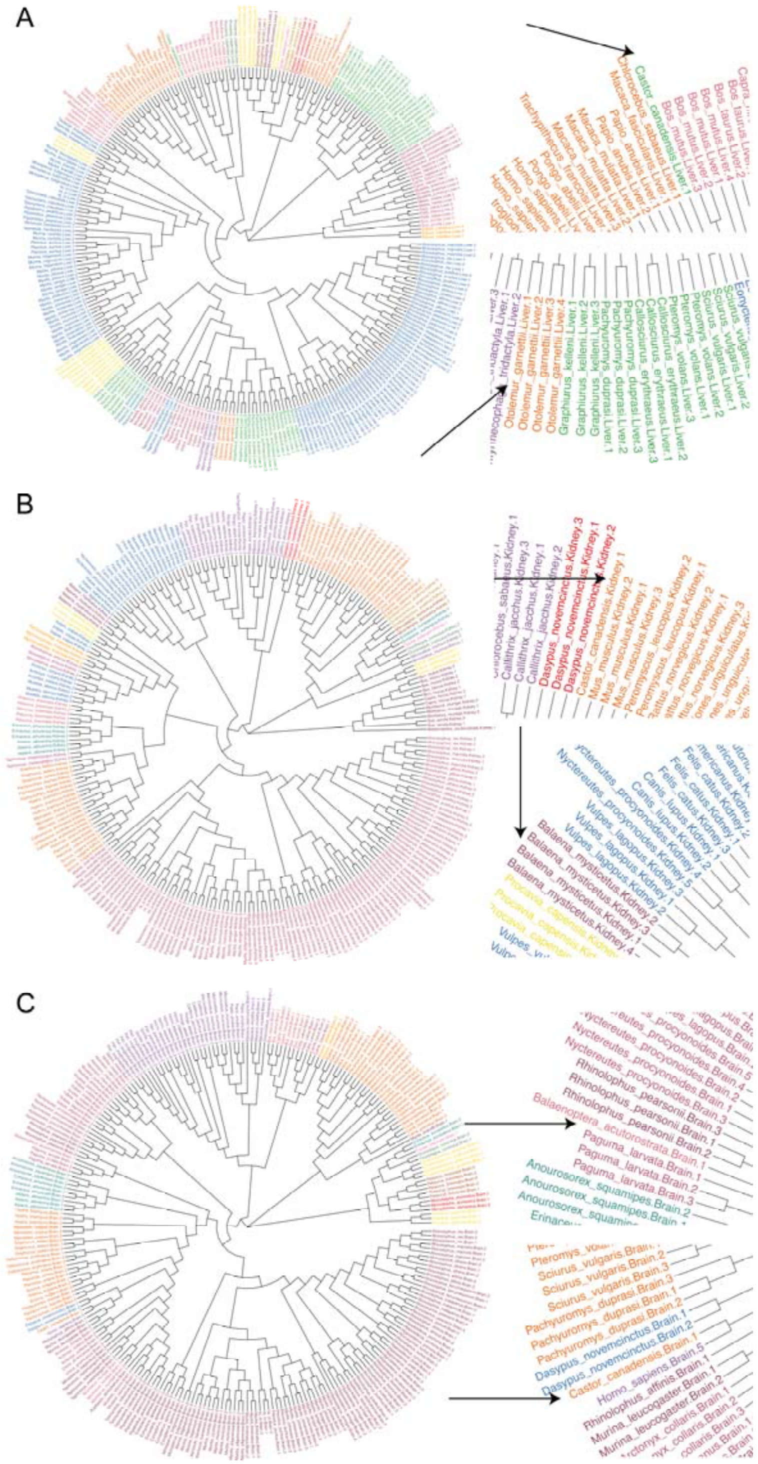

Figure R6: Sample clustering before outlier removal. The distance between samples is 1-Pearson correlation coefficient, and the tree is constructed by the neighbor-joining (NJ) method. (*A*) liver, (*B*) kidney and (*C*) brain.

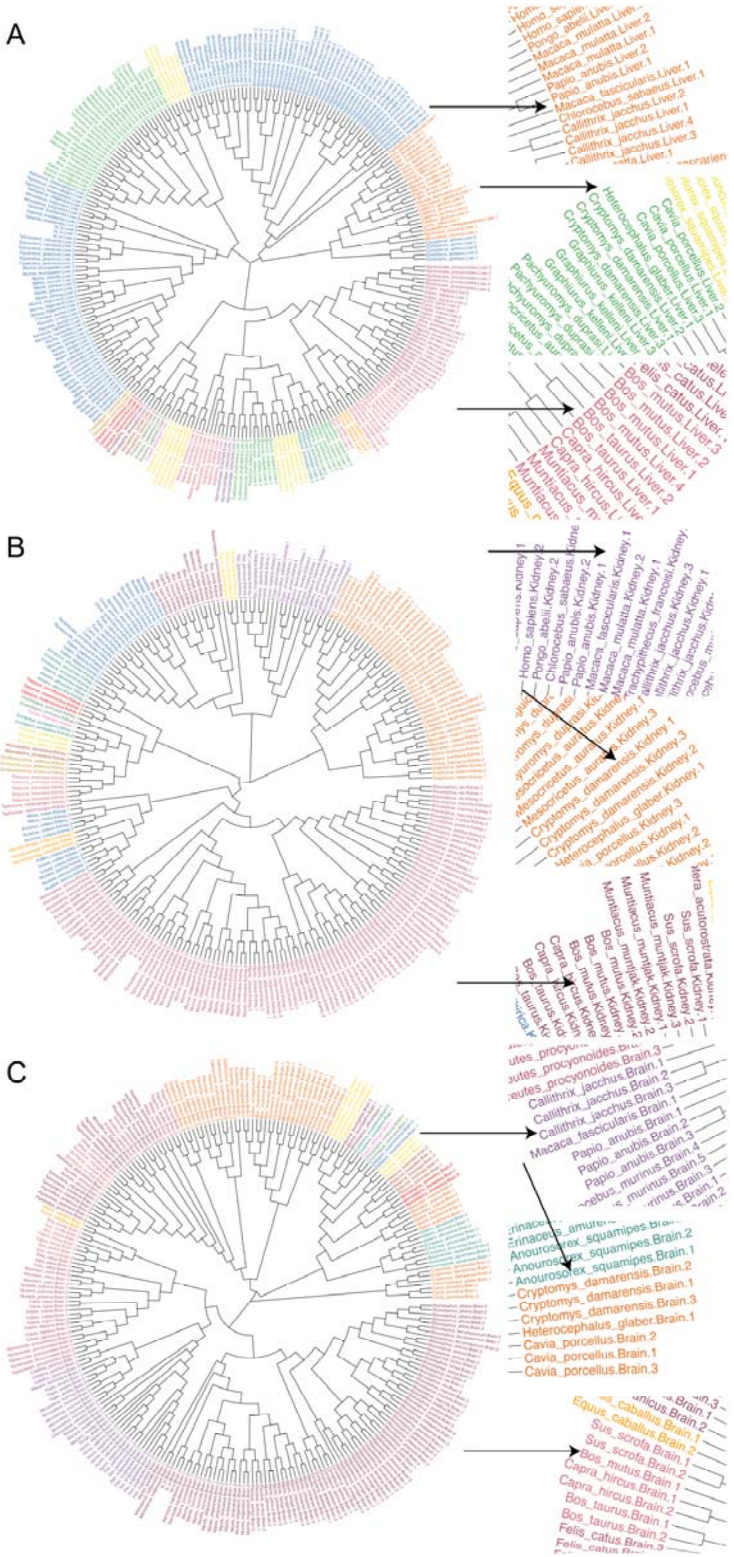

Figure R7: Sample clustering after outlier removal. The distance between samples is 1-Pearson correlation coefficient, and the tree is constructed by the neighbor-joining (NJ) method. (*A*) liver, (*B*) kidney and (*C*) brain.

The three tissues generated by our laboratory were processed simultaneously – the brain data part of our recent study (Zhu et al, 2023). Moreover, many studies have shown that the expression pattern of the brain is significantly different from that of the liver and kidney (Brawand *et al*, 2011; Cardoso-Moreira *et al*, 2019; Fukushima & Pollock, 2020; Fushan *et al*, 2015; Ma *et al*, 2016; Ma *et al*, 2015), so the higher number of genes in the brain may be a tissue-specific difference. Another possibility is that, when calculating Tau, we used all species for each gene, thereby introducing many zero values. In our revision, we repeated the analysis and only included species with corresponding orthologous genes (please see **Comment 3-4** for details). In our new results, the brain is not the tissue with the most species-specific gene expression.

If we understand your last question correctly, you indicate that the PGLS analysis should consider technological replicates. In PGLS, we did not treat each repetition as an observation. Instead, the mean value of all repetitions was calculated in each tissue as the expression of the species (**Lines 712-714**) [To identify genes with the expression related to longevity, three evolution models were tested for gene expression in each tissue (mean value of log_2-scaled TMM-RPKM)]. However, one cannot consider the variation between replicates in GLS. We label the technical duplicate samples generated from this study (e.g., Technical1, Technical2) in **Dataset EV1**.

Comment 3-3:

2) Lack of age information for animals used in the study: While it is understandable that it can be difficult to compile comparable data across different studies, it is important to consider that gene expression changes with age. It is possible that the authors did not study comparable time points across species and that their results (e.g. finding mRNA surveillance negatively correlated with lifespan) are confounded by age-related changes in gene expression due to study bias in long-lived species.

For example, the naked mole rat samples in the study include not only young adult samples, as in most of the other datasets, but also samples from 20-year-old NMRs. This means that their average expression value would incorporate age-related changes in expression for this species. The same bias may be present in other long-lived animals. The authors should provide additional information showing that their data is comparable and exclude other samples (the dataset also includes a limited number of juvenile samples), or analyse the data separately.

It is important to note that a significant proportion of the data was generated either in the present study or in the Fushan et al. paper, which shares some

authors with this study. Therefore, age information should be available for at least these samples.

Response 3-3:

Thank you for this comment. We agree that chronological age of samples can confound results. Please see **response 2-3**.

After removing the abnormal samples (older and outlier), we found that the biological processes related to transcriptional fidelity play important roles in the evolution of mammalian lifespan (**Lines 367-379**) [Several common pathways are found to show correlation with longevity traits in the three tissues examined (**Fig 2A, Dataset EV7**). Positively correlated genes showed enrichment for transcription and translation fidelity pathways such as eukaryotic translation Initiation, eukaryotic translation elongation, nonsense-mediated decay (NMD), peptide chain elongation and translation (**Fig 3A-B**), while the tRNA aminoacylation pathway was enriched for negatively correlated genes. The regulation of translation fidelity affects the lifespan of many organisms (Martinez-Miguel *et al*, 2021; von der Haar *et al*, 2017). For example, the naked mole-rat has higher translational fidelity than the mouse (Azpurua *et al*, 2013). Transfer RNA aminoacylation is inhibited in senescent cells to limit protein synthesis errors (Anisimova *et al*, 2020). In addition, seryl-tRNA can directly bind to telomere repeat sequences, leading to telomere shortening and cell senescence (Li *et al*, 2019).].

Overall, by analyzing the effect of age, we demonstrate that our results are not confounded by age if samples of different ages after sexual maturity are compared. We also revised our previous discussion about this limitation in the Conclusions section (**Lines 487-489**) [Finally, while it is difficult to estimate the actual age of wild samples accurately, these traits and factors should be explored in future studies]. We also traced the sample records of Fushan *et al* (2015) and were not able to obtain accurate age data. However, authors of this study confirm all samples were from old/mature individuals.

Comment 3-4:

3) Tissue and species specificity analysis: In addition to the potential impact of batch correction, it is possible that gene expression levels are confounding this analysis as the authors do not seem to scale the gene expression levels. Highly expressed genes can result in higher variation and higher tau values. If the calculation includes a correction for this, the authors should mention it in the methods.

I am also surprised that there is not even one gene that is stably expressed across mammals, including housekeeping genes. It is possible that the tau index does not provide enough resolution for this analysis, or that the threshold should be changed considering the breadth of the sampling.

When the authors say they used the sum of the tau index for pathway analysis, do they mean the average? Otherwise, this value would be dramatically affected by the number of genes in a pathway.

Response 3-4:

We acknowledge that highly expressed genes can result in higher variation and tau values, potentially affecting the analysis. In addition to batch effects removal, we removed genes whose total expression of all samples accounted for 5% of the expression of the entire data set (**Lines 585-590**) [Finally for 18,553 genes, abnormally low-expressed genes that is, genes whose expression levels were less than 10 in 4 or more samples were filtered before normalization (2,564 genes were removed). And, abnormally high expression genes, that is, genes whose total expression of all samples accounted for 5% of the expression of the entire data set was also removed (1 gene was removed)]. We performed $\log_2$ conversion for on gene expression value (**Lines 606-608**) [The R software package edgeR (Robinson et al, 2010) was used to normalize the library size and gene length (based on humans) by $\log_2$(TMM-RPKM + 1).]. During manuscript revision, we made this clear in the Methods (**Lines 609-610**) [$\log_2$-transformation reduced the effect of higher expression value on the analysis and the variation among replicates].

No gene was expressed stably across mammals because we included all species in the analyses for each ortholog, which brings many zero values into the gene expression analysis. This is clearly undesirable. During revision, we improved our analysis and clarified our approach in the Methods (**Lines 625-627**) [For each ortholog, only species with corresponding orthologous and expression values were considered in analyses]. This approach largely reduces the impacts of zero values on the results. After repeating the analysis, we detected broadly expressed genes containing essential genes. The manuscript includes our new results (**Lines 159-210** and **Fig R8**): [The specificity index (Tau or τ) for gene expression was calculated to characterize genes with species-specific expression patterns in a tissue (**Dataset EV3** and **Methods**). Tau ranges from 0 to 1 and indicates how broadly (<0.2) or specific (>0.8) a gene is expressed (Yanai *et al*, 2005). Compared to the liver and kidney, the brain presented the lowest number of genes showing species-specific expression (792, 1,410, and 2,401 genes for the brain, kidney, and liver) (**Fig 1E**). In parallel, the brain had the highest number of broadly expressed genes (123, 85, and 37 genes for the brain, kidney, and liver) (**Fig 1E**). Broadly expressed genes likely reflect that they contribute to core biological processes of each organ. For example, amyloid precursor protein (*APP*) (τ = 0.11) was widely expressed in the brain. This gene participates in many important brain functions, including synaptogenesis, neuron cell

proliferation, and differentiation (Czeczor & McGee, 2017). The most commonly expressed gene in the kidney was electron-transferring flavoprotein α-subunit (*ETFA*) (τ = 0.15). Inactivation of *ETFA* leads to multiple acyl-CoA dehydrogenase deficiency, a serious mitochondrial disease (Kim *et al*, 2013). Complement protein 3 (*C3*) (τ = 0.11), a gene widely expressed in the liver, plays a central role in all three complement activation pathways (Reis *et al*, 2006).

In parallel, species-specific genes mainly reflect the unique phenotypes or adaptations of particular groups. The gene with the highest species-specific index in the brain was *CXCR3* (τ = 0.98). The primary role of this gene is to participate in inflammation as a chemokine receptor (Long & Jaiswal, 2000), and it may contribute to mouse cognition and behavior (Blank *et al*, 2016). The top species-specific gene in the kidney was glutamate metabotropic receptor 1 (*GRM1*) (τ = 0.98), a gene that regulates cell proliferation (Martino *et al*, 2013). Deficiency of *GRM1* led to the inability to maintain the morphology and intracellular signal transduction in kidney podocytes (Puliti *et al*, 2011). *GRM1* was also highly expressed in long-lived animals, such as bats, humans, and naked mole-rats. This may be related to the supreme cell vitality of long-lived animals (Link, 2019). In the liver, the most species-specific genes, such as *SKOR1* (τ = 0.99), *NWD1* (τ = 0.99), and *KIAA1549L* (τ = 0.99), are associated with cancers (Correa *et al*, 2014; Heiss & Brenner, 2017; Sluimer *et al*, 2023) (**Dataset EV3**). SKI family transcriptional corepressor 1 (*SKOR1*) is also associated with type 2 diabetes. NK-derived exocrine miR-1249-3p directly acts on *SKOR1* to regulate glucose homeostasis and remission of insulin resistance (Wang *et al*, 2021).

Pathway enrichment analysis was performed under a polygenic model to characterize pathways showing expression specificity across species in each organ (Daub *et al*, 2013; Daub *et al*, 2017) (**Fig 1F**, **Dataset EV4**, and **Methods**). Pathways related to Alzheimer's disease, such as the NF-κB signaling pathway (Score = 43.80, $P = 2.49\times10^{-6}$), primary immunodeficiency (Score = 20.94, $P = 2.49\times10^{-6}$), and tight junction interactions (Score = 26.59, $P = 2.49\times10^{-6}$) were significantly enriched by genes showing expression specificity in the brain. In liver and kidney, genes with species-specific expression enriched for cell growth and communication pathways such as nicotine addiction (Liver: Score = 66.84, $P = 2.49\times10^{-6}$; Kidney: Score = 26.30, $P = 2.49\times10^{-6}$), GPCRs class A Rhodopsin-like (Liver: Score = 23.56, $P = 2.49\times10^{-6}$; Kidney: Score = 58.67, $P = 2.49\times10^{-6}$), NCAM1 interactions (Liver: Score = 20.34, $P = 3.99\times10^{-5}$; Kidney: Score = 22.30, $P = 2.49\times10^{-6}$), and voltage-gated potassium channels (Liver: Score = 20.79, $P = 2.49\times10^{-6}$; Kidney: Score = 23.86, $P = 2.49\times10^{-6}$).]

We used the PolySel pipeline (Daub *et al*, 2013; Daub *et al*, 2017) to detect the longevity-correlated pathways by considering the cumulative effect of

genes. The "SUMSTAT" score is the sum of Tau scores of genes in the set of interest ('pathway'). Here, the sum of Tau is not the average value. The significant pathway was inferred with the SUMSTAT score of the null distribution with random gene sets of the same size. For example, if a pathway includes 20 genes, the SUMSTAT score of these 20 genes is calculated, i.e., the observed SUMSTAT score. Next, we sampled 20 genes at random from all genes in all pathways 400,000 times and computed the SUMSTAT score each time. The *P* value for a pathway was estimated by comparing the observed SUMSTAT score with the random SUMSTAT scores. The "size" of each pathway is also taken into account (too few (<10) or too many (>1000) genes were not considered) when calculating the *P* value in PolySel, so the significance of the pathway will not increase because of more genes (please see https://github.com/CMPG/polysel). In addition, redundant gene sets (overlapping genes with other gene sets) are removed by a pruning process: the genes of the highest-scoring gene set will be removed from all lower-ranked gene sets and tested against the remaining gene sets. This process is repeated until no sets are left to be tested. This process reduces the impact of large gene sets.

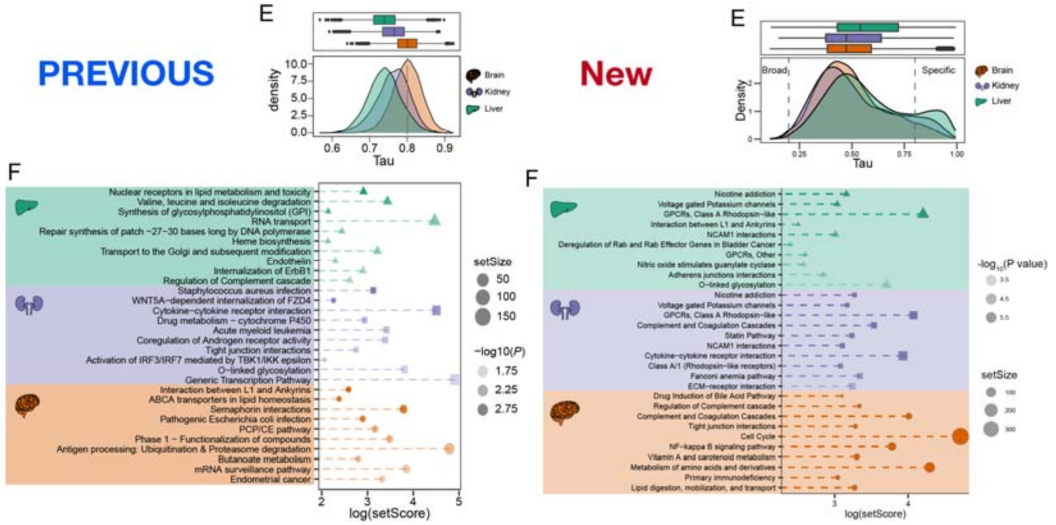

Fig R8: Revisions in Fig 1E and F.

Comment 3-5:

4) Longevity-related genes: The authors found that only 76 out of 974 longevity-regulating genes were associated with lifespan across phylogeny. Is this number statistically significant?

Response 3-5:

Thank you for your suggestion. After removing the old and outlier samples (please see **response 2-3** and **3-2** for details), 33 out of 974

longevity-correlated genes were associated with lifespan across the phylogeny. We conducted Fisher's exact tests and obtained a *P* value of 0.03 (greater, odds ratio is 1.42). The Results now reads (**Lines 357-366**): [Next, we explored the intersection between longevity-correlated genes and genes with known effects on aging in model organisms documented in GenAge (*n* = 974 genes) (Tacutu *et al*, 2013). Our longevity-correlated genes showed enrichment for GenAge genes (*n* = 33; e.g., *CEBPB*, *MYC*, and *TERT*) (Fisher's exact test: *P* = 0.03, greater, odds ratio = 1.42). Nevertheless, most longevity-correlated genes in our dataset are not known as aging-related genes, indicating that most aging-related genes do not serve as a basis for the evolution of longevity across species, although they have been shown to directly contribute to lifespan control in one or more model species.]

Comment 3-6:

5) Expression-trait correlation plots: I am confused by Fig 2E-G, supplementary figures S8, and S12, which show correlations between some of the genes the authors emphasize and longevity traits. Although the p values are quite significant, these plots show almost no correlation. Is this due to the effect of phylogeny? If so, perhaps showing correlations within taxa would be more informative. Otherwise, despite the significant p values, it does not appear that any of these traits explain a significant portion of the variation in the data (perhaps r2 values from PGLS analysis would be helpful in this regard).

Response 3-6:

Sorry for this confusion. The longevity-related traits and gene expression data were log-transformed and scaled (R function 'scale') (**Fig R9**). After removing the old and outlier samples, we have improved the visualization.

In addition, the effect of the phylogeny on the gene expression by comparing three models for each gene was considered in PGLS analyses, i.e., OU (Ornstein-Uhlenbeck Motion), the ordinary least squares (OLS), and the Brownian model. The best-supported model of most genes was OLS (i.e., no phylogeny relationships), indicating that phylogeny did not significantly influence the expression of these genes. Otherwise, the effect of phylogeny has been controlled when the OU or Brownian model was best supported. We also used RSE (residual standard error) instead of *r2* values to represent the quality of model fitting since the result from an evolutionary model PGLS is not directly comparable to a null model (see discussion in Whitney *et al* (2011)). During revision, we modified the top 5 up/down-regulated genes to the top five up/down-regulated genes significantly associated with at least two or more longevity traits (longevity-correlated genes). This increases the robustness of the correlation between gene expression and longevity. The definition of

longevity-correlated genes has been added to the Results (**Lines 223-225**) [Significant genes for more than two longevity-related traits were defined as longevity-correlated genes (*n* = 669, **Table 1**, **Methods**)], and Methods (**Lines 736-737**) [We denote genes associated with two or more longevity traits as longevity-correlated genes.]

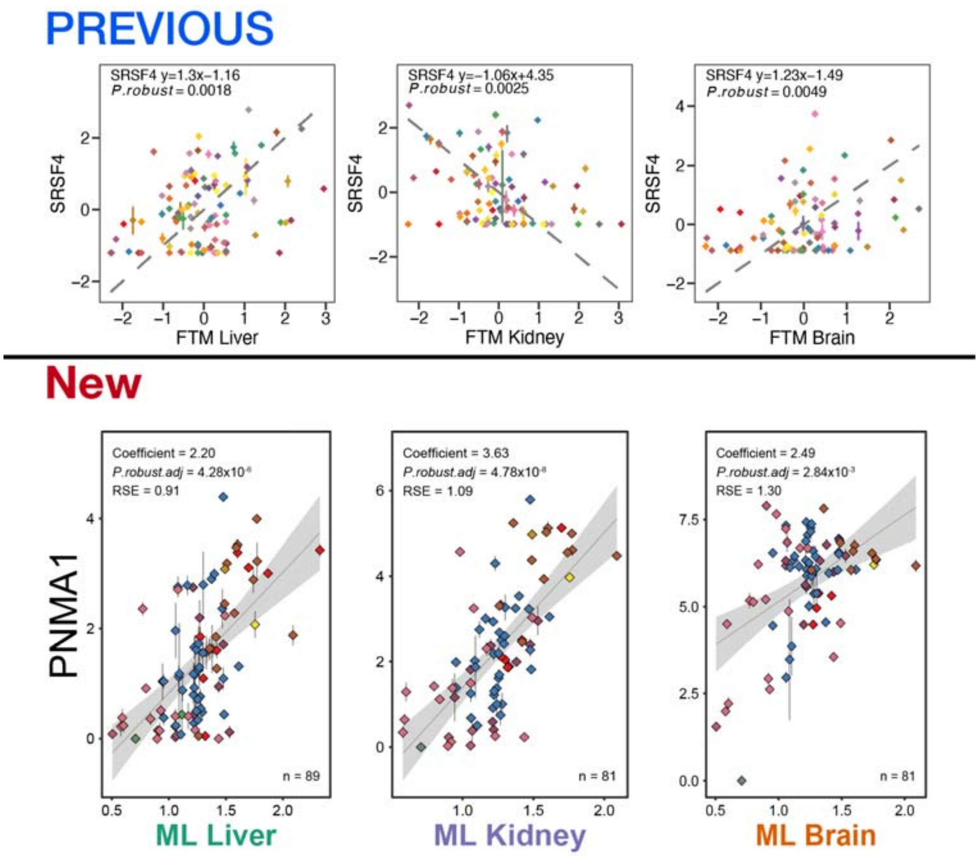

Figure R9 Revision in Fig 2E-G.

Comment 3-7:

6) Selection signatures: The authors used the absolute number of years (ML >30), rather than a metric relative to the taxa. I am concerned that this may lead to the study of taxa with longer lifespans rather than species that have evolved longer lifespans. Can the authors provide more information on this choice and its potential impact on the results? I think it might be more helpful to perform a taxa-specific analysis to better understand selection signatures.

Response 3-7:

Thank you very much for pointing this out. To consider biological and statistical significance, we refer to previous studies to delineate longevity species (Jobson *et al*, 2010; Zhu *et al*, 2023). The cut-off value of 26 includes very

long-lived species (e.g., human and bowhead whale) and relatively long-lived species (e.g., when considering body mass: the naked mole rat and Brandt's bat). Specially, we used the Partitioning Around Medoids (PAM) algorithm to cluster the longevity of 974 species into two groups (long-lived group and non-long-lived group) by setting the parameter *k* (the number of clusters) as two (Kaufman & Rousseeuw, 1990). The range of maximum lifespan for the two clusters was 1.00–17.30 years (428 species, mean = 8.88 years) and 17.40–211 years (536 species, mean = 30.49 years). We used the mean ages of long-lived groups (30 years) as the cut-off value, which is also close to 26 years that suggested by Jobson *et al* (2010).

Moreover, since we also estimated longevity for several species that do not have records in the documents, none of the longevity estimates are more than 30 years (among the 103 species in this study), which could decrease the potential errors resulting from imputation. We have added this information to the Methods (**Lines 752-763**) [To define a cutoff for long-lived species, we used the Partitioning Around Medoids (PAM) algorithm to cluster longevity data of 974 species (Zhu *et al*, 2023) into two groups (long-lived group and non-long-lived group) by setting the parameter *k* (the number of clusters) as two (Kaufman & Rousseeuw, 1990). The range of maximum lifespan was 1.00–17.30 years (428 species, mean = 8.88 years) for the non-long-lived group and 17.40–211 years (536 species, mean = 30.49 years) for the long-lived group. Therefore, we set the ML cut-off of long-lived mammals at 30 years, which is also close to the 26 years cut-off suggested by Jobson *et al* (2010). Moreover, none of the estimated maximum lifespans are larger than 30 years (among the 103 species in this study), which could alleviate the potential errors resulting from imputation.]

We appreciate your suggestion to perform a taxa-specific analysis. We selected two orders (Rodentia and Chiroptera) for RELAX and PGLS analyses. These orders include species with diverse lifespans (e.g., 18 species of Rodentia) and particularly long-lived species (e.g., 36 bats in Chiroptera). As for the RELAX analyses, few common enriched pathways were shared by the two orders, and these enriched pathways were also enriched when considering the entire 103-species data set (**Lines 448-451**) [We further stratified our data set and considered two subgroups (i.e., Chiroptera and Rodentia). Pathways such as Wnt signaling, pluripotency, and HIV Infection were under relaxed selection in both subgroups (**Table EV4**).]

For PGLS analyses, the enriched pathways shared by two subgroups were highly similar to analyses of total species, which supports the importance of transcriptional fidelity for lifespan evolution (**Lines 451-455**) [Specially, pathways related to transcription and translation fidelity and DNA replications were also significantly enriched by longevity-correlated genes in both subgroups, as found in the analyses of the complete 103-species dataset

(**Dataset EV13**), further evidencing those pathways are important for lifespan evolution.]

We have added those results in the revision and updated the method. Thank you again for this constructive suggestion.

References

Brawand D, Soumillon M, Necsulea A, Julien P, Csardi G, Harrigan P, Weier M, Liechti A, Aximu-Petri A, Kircher M *et al* (2011) The evolution of gene expression levels in mammalian organs. *Nature* 478: 343-348

Cardoso-Moreira M, Halbert J, Valloton D, Velten B, Chen C, Shao Y, Liechti A, Ascencao K, Rummel C, Ovchinnikova S *et al* (2019) Gene expression across mammalian organ development. *Nature*

Daub JT, Hofer T, Cutivet E, Dupanloup I, Quintana-Murci L, Robinson-Rechavi M, Excoffier L (2013) Evidence for polygenic adaptation to pathogens in the human genome. *Mol Biol Evol* 30: 1544-1558

Daub JT, Moretti S, Davydov, II, Excoffier L, Robinson-Rechavi M (2017) Detection of Pathways Affected by Positive Selection in Primate Lineages Ancestral to Humans. *Mol Biol Evol* 34: 1391-1402

Fukushima K, Pollock DD (2020) Amalgamated cross-species transcriptomes reveal organ-specific propensity in gene expression evolution. *Nat Commun* 11: 4459

Fushan AA, Turanov AA, Lee SG, Kim EB, Lobanov AV, Yim SH, Buffenstein R, Lee SR, Chang KT, Rhee H *et al* (2015) Gene expression defines natural changes in mammalian lifespan. *Aging Cell* 14: 352-365

Jobson RW, Nabholz B, Galtier N (2010) An evolutionary genome scan for longevity-related natural selection in mammals. *Mol Biol Evol* 27: 840-847

Kaufman L, Rousseeuw PJ (1990) Partitioning Around Medoids (Program PAM). In: *Finding Groups in Data*, pp. 68-125.

Ma S, Upneja A, Galecki A, Tsai YM, Burant CF, Raskind S, Zhang Q, Zhang ZD, Seluanov A, Gorbunova V *et al* (2016) Cell culture-based profiling across mammals reveals DNA repair and metabolism as determinants of species longevity. *Elife* 5

Ma S, Yim SH, Lee SG, Kim EB, Lee SR, Chang KT, Buffenstein R, Lewis KN, Park TJ, Miller RA *et al* (2015) Organization of the Mammalian Metabolome according to Organ Function, Lineage Specialization, and Longevity. *Cell Metab* 22: 332-343

Whitney KD, Boussau B, Baack EJ, Garland T, Jr. (2011) Drift and Genome Complexity Revisited. *PLOS Genetics* 7: e1002092

Zhu P, Liu W, Zhang X, Li M, Liu G, Yu Y, Li Z, Li X, Du J, Wang X *et al* (2023) Correlated evolution of social organization and lifespan in mammals. *Nat Commun* 14: 372

Dear Dr. Zhou,

Congratulations on a great revision! Overall, the referees have been positive and are in support of publication. Referee 2 has raised a minor concern that we ask you at least respond to in a new point by point response or perform the request if you choose.

Thank you for the opportunity to consider your work for publication, I look forward to your revision so we can move forward with your manuscript.

Kind regards,

Kelly

Kelly M Anderson, PhD
Editor
The EMBO Journal
k.anderson@embojournal.org

Further information is available in our Guide For Authors: https://www.embopress.org/page/journal/14602075/authorguide

Use the link below to submit your revision:

https://emboj.msubmit.net/cgi-bin/main.plex

Referee #2:

The authors did a great job responding to the questions and the manuscript has been greatly improved.

Referee #3:

This is important work, providing new and integrated data, not only analyzing varied taxa to understand the underpinnings of longevity evolution but also allowing further research by making data available.

The authors provided an extensive revision and addressed all my comments, some of which required complete reanalysis. The authors updated the analysis and made the required changes in the manuscript. I am very happy to support the publication of this manuscript.

I have only a minor suggestion to increase the impact and reusability of the dataset generated - I would like to suggest the authors consider creating an R data object with expression and metadata corresponding to their integrated (batch-corrected) dataset, to make it easy to use by others, for sure increasing the reach of the work. However, this is not required but just a minor suggestion as all raw data is deposited to a public repository (please note that the embargo needs to be lifted with the publication, it is not available at the moment).

Detailed responses to reviewers

All comments provided by reviewers are in gray italics, and our responses are in blue. All revisions in the manuscript are marked in red.

REVIEWERS COMMENTS

Reviewer #2

Comment 2-1:

The authors did a great job responding to the questions and the manuscript has been greatly improved.

Response 2-1:

Thank you very much for the positive feedback. We sincerely appreciate your contribution to our study.

Reviewer #3

Comment 3-1:

This is important work, providing new and integrated data, not only analyzing varied taxa to understand the underpinnings of longevity evolution but also allowing further research by making data available.

The authors provided an extensive revision and addressed all my comments, some of which required complete reanalysis. The authors updated the analysis and made the required changes in the manuscript. I am very happy to support the publication of this manuscript.

Response 3-1:

Thank you for your positive feedback. We greatly appreciate your professional comments and insightful suggestions that significantly improved our manuscript. We sincerely appreciate your time and energy devoted to the review process.

Comment 3-2:

I have only a minor suggestion to increase the impact and reusability of the dataset generated - I would like to suggest the authors consider creating an R data object with expression and metadata corresponding to their integrated (batch-corrected) dataset, to make it easy to use by others, for sure increasing the reach of the work. However, this is not required but just a minor suggestion as all raw data is deposited to a public repository (please note that the embargo needs to be lifted with the publication, it is not available at the moment).

Response 3-2:

Thank you for your suggestion. We have uploaded the integrative data in R object to the GitHub and updated the 'Data availability' section in the manuscript (Lines 834-836) [The gene expression matrix and corresponding meta information are available at GitHub (https://github.com/liu-wq/expressionML.git).].

2nd Revision - Editorial Decision 22nd May 2023

Dear Dr. Zhou,

Congratulations on an excellent manuscript, I am pleased to inform you that your manuscript has been accepted for publication in the EMBO Journal. Thank you for your comprehensive response to the referee concerns. It has been a pleasure to work with you to get this to the acceptance stage.

I will begin the final checks on your manuscript before submitting to the publisher next week. Once at the publisher, it will take about 3 weeks for your manuscript to be published online. As a reminder, the entire review process, including referee concerns and your point-by-point response, will be available to readers.

I will be in touch throughout the final editorial process until publication. In the meantime, I hope you find time to celebrate!

Kind regards,

Kelly

Kelly M Anderson, PhD
Editor
The EMBO Journal
k.anderson@embojournal.org

Please note that it is EMBO Journal policy for the transcript of the editorial process (containing referee reports and your response letter) to be published as an online supplement to each paper. If you do NOT want this, you will need to inform the Editorial Office via email immediately. More information is available here:
https://www.embopress.org/page/journal/14602075/authorguide#transparentprocess

Your manuscript will be processed for publication in the journal by EMBO Press. Manuscripts in the PDF and electronic editions of The EMBO Journal will be copy edited, and you will be provided with page proofs prior to publication. Please note that supplementary information is not included in the proofs.

You will be contacted by Wiley Author Services to complete licensing and payment information. The required 'Page Charges Authorization Form' is available here: https://www.embopress.org/pb-assets/embo-site/tej_apc.pdf - please download and complete the form and return to embopressproduction@wiley.com

EMBO Press participates in many Publish and Read agreements that allow authors to publish Open Access with reduced/no publication charges. Check your eligibility: https://authorservices.wiley.com/author-resources/Journal-Authors/open-access/affiliation-policies-payments/index.html

Should you be planning a Press Release on your article, please get in contact with embojournal@wiley.com as early as possible, in order to coordinate publication and release dates.

** Click here to be directed to your login page: https://emboj.msubmit.net

EMBO Press Author Checklist

Corresponding Author Name: Xuming Zhou	
Journal Submitted to: The EMBO Journal	
Manuscript Number: EMBOJ-2022-112740	

USEFUL LINKS FOR COMPLETING THIS FORM
The EMBO Journal - Author Guidelines
EMBO Reports - Author Guidelines
Molecular Systems Biology - Author Guidelines
EMBO Molecular Medicine - Author Guidelines

Reporting Checklist for Life Science Articles (updated January
This checklist is adapted from Materials Design Analysis Reporting (MDAR) Checklist for Authors. MDAR establishes a minimum set of requirements in transparent reporting in the life sciences (see Statement of Task: 10.31222/osf.io/9sm4x). Please follow the journal's guidelines in preparing your
Please note that a copy of this checklist will be published alongside your article.

Abridged guidelines for figures
1. Data
The data shown in figures should satisfy the following conditions:
- the data were obtained and processed according to the field's best practice and are presented to reflect the results of the experiments in an accurate and unbiased manner.
- ideally, figure panels should include only measurements that are directly comparable to each other and obtained with the same assay.
- plots include clearly labeled error bars for independent experiments and sample sizes. Unless justified, error bars should not be shown for technical
- if n<5, the individual data points from each experiment should be plotted. Any statistical test employed should be justified.
- Source Data should be included to report the data underlying figures according to the guidelines set out in the authorship guidelines on Data

2. Captions
Each figure caption should contain the following information, for each panel where they are relevant:
- a specification of the experimental system investigated (eg cell line, species name).
- the assay(s) and method(s) used to carry out the reported observations and measurements.
- an explicit mention of the biological and chemical entity(ies) that are being measured.
- an explicit mention of the biological and chemical entity(ies) that are altered/varied/perturbed in a controlled manner.
- the exact sample size (n) for each experimental group/condition, given as a number, not a range;
- a description of the sample collection allowing the reader to understand whether the samples represent technical or biological replicates (including how many animals, litters, cultures, etc.).
- a statement of how many times the experiment shown was independently replicated in the laboratory.
- definitions of statistical methods and measures:
  - common tests, such as t-test (please specify whether paired vs. unpaired), simple χ2 tests, Wilcoxon and Mann-Whitney tests, can be unambiguously identified by name only, but more complex techniques should be described in the methods section;
  - are tests one-sided or two-sided?
  - are there adjustments for multiple comparisons?
  - exact statistical test results, e.g., P values = x but not P values < x;
  - definition of 'center values' as median or average;
  - definition of error bars as s.d. or s.e.m.

Please complete ALL of the questions below.
Select "Not Applicable" only when the requested information is not relevant for your study.

Materials

Newly Created Materials	Information included in the manuscript?	In which section is the information available? (Reagents and Tools Table, Materials and Methods, Figures, Data Availability Section)
New materials and reagents need to be available; do any restrictions apply?	Not Applicable	

Antibodies	Information included in the manuscript?	In which section is the information available? (Reagents and Tools Table, Materials and Methods, Figures, Data Availability Section)
For **antibodies** provide the following information:<br>- Commercial antibodies: RRID (if possible) or supplier name, catalogue number and or/clone number<br>- Non-commercial: RRID or citation	Not Applicable	

DNA and RNA sequences	Information included in the manuscript?	In which section is the information available? (Reagents and Tools Table, Materials and Methods, Figures, Data Availability Section)
**Short novel DNA or RNA including primers, probes**: provide the sequences.	Not Applicable	

Cell materials	Information included in the manuscript?	In which section is the information available? (Reagents and Tools Table, Materials and Methods, Figures, Data Availability Section)
**Cell lines:** Provide species information, strain. Provide accession number in repository **OR** supplier name, catalog number, clone number, and/**OR** RRID.	Not Applicable	
**Primary cultures:** Provide species, strain, sex of origin, genetic modification status.	Not Applicable	
Report if the cell lines were recently **authenticated** (e.g., by STR profiling) and tested for mycoplasma contamination.	Not Applicable	

Experimental animals	Information included in the manuscript?	In which section is the information available? (Reagents and Tools Table, Materials and Methods, Figures, Data Availability Section)
**Laboratory animals or Model organisms:** Provide species, strain, sex, age, genetic modification status. Provide accession number in repository **OR** supplier name, catalog number, clone number, **OR** RRID.	Not Applicable	
**Animal observed in or captured from the field:** Provide species, sex, and age where possible.	Yes	Table_EV1
Please detail **housing and husbandry conditions**.	Not Applicable	

Plants and microbes	Information included in the manuscript?	In which section is the information available? (Reagents and Tools Table, Materials and Methods, Figures, Data Availability Section)
**Plants:** provide species and strain, ecotype and cultivar where relevant, unique accession number if available, and source (including location for collected wild specimens).	Not Applicable	
**Microbes:** provide species and strain, unique accession number if available, and source.	Not Applicable	

Human research participants	Information included in the manuscript?	In which section is the information available? (Reagents and Tools Table, Materials and Methods, Figures, Data Availability Section)
If collected and within the bounds of privacy constraints report on age, sex and gender or ethnicity for all study participants.	Not Applicable	

Core facilities	Information included in the manuscript?	In which section is the information available? (Reagents and Tools Table, Materials and Methods, Figures, Data Availability Section)
If your work benefited from core facilities, was their service mentioned in the acknowledgments section?	Not Applicable	

Design

Study protocol	Information included in the manuscript?	In which section is the information available? (Reagents and Tools Table, Materials and Methods, Figures, Data Availability Section)
If study protocol has been **pre-registered, provide DOI in the manuscript**. For clinical trials, provide the trial registration number **OR** cite DOI.	Not Applicable	
Report the **clinical trial registration number** (at ClinicalTrials.gov or equivalent), where applicable.	Not Applicable	

Laboratory protocol	Information included in the manuscript?	In which section is the information available? (Reagents and Tools Table, Materials and Methods, Figures, Data Availability Section)
Provide DOI OR other citation details if **external detailed step-by-step protocols** are available.	Not Applicable	

Experimental study design and statistics	Information included in the manuscript?	In which section is the information available? (Reagents and Tools Table, Materials and Methods, Figures, Data Availability Section)
Include a statement about **sample size** estimate even if no statistical methods were used.	Not Applicable	
Were any steps taken to minimize the effects of subjective bias when allocating animals/samples to treatment (e.g. **randomization procedure**)? If yes, have they been described?	Not Applicable	
Include a statement about **blinding** even if no blinding was done.	Not Applicable	
Describe **inclusion/exclusion criteria** if samples or animals were excluded from the analysis. Were the criteria pre-established?<br><br>If sample or data points were omitted from analysis, report if this was due to attrition or intentional exclusion and provide justification.	Yes	Materials and Methods, Table_EV1
For every figure, are **statistical tests** justified as appropriate? Do the data meet the assumptions of the tests (e.g., normal distribution)? Describe any methods used to assess it. Is there an estimate of variation within each group of data? Is the variance similar between the groups that are being statistically compared?	Yes	Materials and Methods

Sample definition and in-laboratory replication	Information included in the manuscript?	In which section is the information available? (Reagents and Tools Table, Materials and Methods, Figures, Data Availability Section)
In the figure legends: state number of times the experiment was **replicated** in laboratory.	Not Applicable	
In the figure legends: define whether data describe **technical or biological replicates**.	Not Applicable	

Ethics

Ethics	Information included in the manuscript?	In which section is the information available? (Reagents and Tools Table, Materials and Methods, Figures, Data Availability Section)
Studies involving **human participants**: State details of **authority granting ethics approval** (IRB or equivalent committee(s), provide reference number for approval.	Not Applicable	
Studies involving **human participants**: Include a statement confirming that **informed consent** was obtained from all subjects and that the experiments conformed to the principles set out in the WMA Declaration of Helsinki and the Department of Health and Human Services Belmont Report.	Not Applicable	
Studies involving **human participants:** For publication of **patient photos**, include a statement confirming that consent to publish was obtained.	Not Applicable	
Studies involving experimental **animals**: State details of **authority granting ethics approval** (IRB or equivalent committee(s), provide reference number for approval. Include a statement of compliance with ethical regulations.	Not Applicable	
Studies involving **specimen and field samples:** State if relevant **permits** obtained, provide details of authority approving study; if none were required, explain why.	Yes	Materials and Methods

Dual Use Research of Concern (DURC)	Information included in the manuscript?	In which section is the information available? (Reagents and Tools Table, Materials and Methods, Figures, Data Availability Section)
Could your study fall under dual use research restrictions? Please check biosecurity documents and list of **select agents and toxins** (CDC): https://www.selectagents.gov/sat/list.htm	Not Applicable	
If you used a select agent, is the security level of the lab appropriate and reported in the manuscript?	Not Applicable	
If a study is subject to dual use research of concern regulations, is the name of the **authority granting approval and reference number** for the regulatory approval provided in the manuscript?	Not Applicable	

Reporting

The MDAR framework recommends adoption of discipline-specific guidelines, established and endorsed through community initiatives. Journals have their own policy about requiring specific guidelines and recommendations to complement MDAR.

Adherence to community standards	Information included in the manuscript?	In which section is the information available? (Reagents and Tools Table, Materials and Methods, Figures, Data Availability Section)
State if relevant guidelines or checklists (e.g., **ICMJE, MIBBI, ARRIVE, PRISMA**) have been followed or provided.	Not Applicable	
For **tumor marker prognostic studies,** we recommend that you follow the **REMARK** reporting guidelines (see link list at top right). See author guidelines, under 'Reporting Guidelines'. Please confirm you have followed these guidelines.	Not Applicable	
For **phase II and III randomized controlled trials**, please refer to the **CONSORT** flow diagram (see link list at top right) and submit the CONSORT checklist (see link list at top right) with your submission. See author guidelines, under 'Reporting Guidelines'. Please confirm you have submitted this list.	Not Applicable	

Data Availability

Data availability	Information included in the manuscript?	In which section is the information available? (Reagents and Tools Table, Materials and Methods, Figures, Data Availability Section)
Have **primary datasets** been deposited according to the journal's guidelines (see 'Data Deposition' section) and the respective accession numbers provided in the Data Availability Section?	Yes	Data Availability
Were **human clinical and genomic datasets** deposited in a public access-controlled repository in accordance to ethical obligations to the patients and to the applicable consent agreement?	Not Applicable	
Are **computational models** that are central and integral to a study available without restrictions in a machine-readable form? Were the relevant accession numbers or links provided?	Not Applicable	
If publicly available data were reused, provide the respective **data citations in the reference list**.	Yes	Results and Discussion, Reference
